# Comparative analysis of common alignment tools for single-cell RNA sequencing

**DOI:** 10.1093/gigascience/giac001

**Published:** 2022-01-27

**Authors:** Ralf Schulze Brüning, Lukas Tombor, Marcel H Schulz, Stefanie Dimmeler, David John

**Affiliations:** Institute of Cardiovascular Regeneration, Theodor-Stern-Kai 7, 60590 Frankfurt, Germany; Cardio-Pulmonary Institute (CPI), Theodor-Stern-Kai 7, 60590 Frankfurt, Germany; Institute of Cardiovascular Regeneration, Theodor-Stern-Kai 7, 60590 Frankfurt, Germany; German Center for Cardiovascular Research (DZHK), Potsdamer Str. 58 10785 Berlin, Germany; Institute of Cardiovascular Regeneration, Theodor-Stern-Kai 7, 60590 Frankfurt, Germany; Cardio-Pulmonary Institute (CPI), Theodor-Stern-Kai 7, 60590 Frankfurt, Germany; German Center for Cardiovascular Research (DZHK), Potsdamer Str. 58 10785 Berlin, Germany; Institute of Cardiovascular Regeneration, Theodor-Stern-Kai 7, 60590 Frankfurt, Germany; Cardio-Pulmonary Institute (CPI), Theodor-Stern-Kai 7, 60590 Frankfurt, Germany; German Center for Cardiovascular Research (DZHK), Potsdamer Str. 58 10785 Berlin, Germany; Institute of Cardiovascular Regeneration, Theodor-Stern-Kai 7, 60590 Frankfurt, Germany; Cardio-Pulmonary Institute (CPI), Theodor-Stern-Kai 7, 60590 Frankfurt, Germany

**Keywords:** benchmarking, single-cell RNA sequencing, mapping-algorithms, aligners, transcriptomics, mappers

## Abstract

**Background:**

With the rise of single-cell RNA sequencing new bioinformatic tools have been developed to handle specific demands, such as quantifying unique molecular identifiers and correcting cell barcodes. Here, we benchmarked several datasets with the most common alignment tools for single-cell RNA sequencing data. We evaluated differences in the whitelisting, gene quantification, overall performance, and potential variations in clustering or detection of differentially expressed genes. We compared the tools Cell Ranger version 6, STARsolo, Kallisto, Alevin, and Alevin-fry on 3 published datasets for human and mouse, sequenced with different versions of the 10X sequencing protocol.

**Results:**

Striking differences were observed in the overall runtime of the mappers. Besides that, Kallisto and Alevin showed variances in the number of valid cells and detected genes per cell. Kallisto reported the highest number of cells; however, we observed an overrepresentation of cells with low gene content and unknown cell type. Conversely, Alevin rarely reported such low-content cells. Further variations were detected in the set of expressed genes. While STARsolo, Cell Ranger 6, Alevin-fry, and Alevin produced similar gene sets, Kallisto detected additional genes from the Vmn and Olfr gene family, which are likely mapping artefacts. We also observed differences in the mitochondrial content of the resulting cells when comparing a prefiltered annotation set to the full annotation set that includes pseudogenes and other biotypes.

**Conclusion:**

Overall, this study provides a detailed comparison of common single-cell RNA sequencing mappers and shows their specific properties on 10X Genomics data.

## Background

Major advances could be achieved in the transcriptomics field by using single-cell RNA sequencing (scRNA-seq) to conduct differential expression analysis, clustering, cell type annotation, and pseudotime analysis on a single-cell level [1]. Analysis of scRNA-seq data helped to reveal new insights into cellular heterogeneity, e.g., the altered phenotypes in circulating immune cells of patients with chronic ischemic heart disease [2] or the transcriptional diversity of aging fibroblasts [3]. However, the analysis of scRNA-seq data is resource intensive and requires deeper knowledge of specific characteristics of each analysis tool. The most resource-intensive step during single-cell next-generation sequencing data analysis is the alignment of reads to a reference genome and/or transcriptome. Therefore, a common question relates to the choice of the best scRNA-seq alignment tool that can be incorporated into a fast, reliable, and reproductive analysis pipeline. Here we evaluated 5 popular alignment tools: Cell Ranger 6 and STARsolo, as well as the pseudo-alignment tools Alevin, Alevin-fry, and Kallisto.

The technological properties of these mappers are summarized in Supplementary Table S1. In general, the Cell Ranger 6 software suite developed for 10X Genomics Chromium platform [4] data uses STAR [5] as the standard alignment tool. STAR, originally designed for bulk-seq data, takes a classical alignment approach by using a maximal mappable seed search; thereby all possible positions of the reads can be determined. In contrast, Kallisto [6], Alevin-fry [7], and Alevin [8] take an alignment-free approach, so-called pseudo-alignment.

The idea of alignment-free RNA-Seq quantification was introduced by Patro et al. [9] with Salfish and promised much faster alignments. Here, *k*-mers of reads and the transcriptome are compared, and no complete alignment between read and reference is computed, which leads to huge speed-ups. Two years later, the Patcher lab introduced Kallisto, a pseudo-alignment algorithm that achieved similar improvements in runtime but with higher alignment accuracy compared to Salfish. In response, Patro et al. published Salmon [10], a reimplementation of their initial Salfish tool that implements a sample-specific bias model that accounts for various biases that prevent high false-positive rates and overall refined expression estimates. With the advent of scRNA-seq, Kallisto introduced the Kalisto-bustools pipeline and Alevin was released as an extension of Salmon to process scRNA-seq data.

Alevin makes use of an improved pseudo-alignment called selective alignment that promises a higher specificity but an increase in runtime compared to its previous implementation. With the release of Alevin-fry, Alevin introduced a custom version of pseudo-alignment that can use a memory-efficient sketch data structure to improve processing speed of large datasets. However, it has been shown that pseudo-alignment tools have limitations in the quantification of genes that have a low level of expression [11].

In contrast to bulk-RNA-seq, preprocessing of scRNA-seq requires specific features. Essential features are cell calling, removing PCR duplicates, and assigning reads to individual genes and cells. These features can be achieved through barcode and UMI sequences, which are sequenced along with the reads. Therefore, the correct handling of barcode and UMI sequences are crucial steps in the processing of scRNA-seq data. Each alignment tool applies different strategies to handle these errors.

The most important step for cell calling is the correction of sequencing errors within the barcodes. Cell Ranger 6, STARsolo, and Kallisto correct barcodes by comparing the sequenced barcodes to a set of all barcodes that are included in the library preparation kit, the so-called whitelist. This whitelist is provided by 10X Genomics. If no exact match for a sequenced barcode can be found in the whitelist, this barcode is replaced with the closest barcode from the whitelist, if the Hamming distance is not >1. Alevin, however, generates a putative whitelist of highly abundant barcodes that exceed a previously defined knee point. Afterwards Alevin assigns error-prone barcodes to the closest barcode from the putative whitelist, while allowing an edit distance of 1.

To remove biases from PCR duplicates (reads with the same mapping position, the same cell barcode) an identical unique molecular identifier (UMI) sequence is required for pooling these PCR duplicates. To correct errors in UMI sequences, Cell Ranger 6 and STARsolo group reads according to their barcode, UMI, and gene annotation, while allowing 1 mismatch (MM) in the UMI sequence. Because error-prone UMIs are rare, they will be replaced by the higher abundant (supposedly correct) UMI. Afterwards a second round is done by grouping the barcode, corrected UMI, and gene annotation. When groups differ only by their gene annotation, the group with the highest read count is kept for UMI counting. The other groups are discarded because these reads originate from the same RNA construct but were mapped to different genes. A detailed description of the whitelisting and UMI correction methods, which are unique for Cell Ranger, can be found on the 10X website [12]. Alevin builds a UMI graph and tries to find a minimal set of transcripts for UMI deduplication [8]. In this process, similar UMIs are corrected. Kallisto applies a naive collapsing method, which removes reads that originate from different molecules but contain the same UMI [6].

The third important preprocessing step of scRNA-seq data is the assignment of reads to individual genes and cells. Here, the alignment tools have striking differences handling these multi-mapped reads. In STARsolo, Cell Ranger 6 and Kallisto multi-mapped reads are discarded when no unique mapping position can be found within the genome/transcriptome. In contrast, Alevin equally divides the counts of a multi-mapped read to all potential mapping positions. The order of necessary steps for quantification, i.e., the alignment and barcode and UMI correction, can vary for each tool. Therefore, Supplementary Table S2 shows this order. Kallisto has the most different order, in which the barcode correction is executed after the alignment and a UMI correction is not performed. The other tools perform the barcode correction before the alignment and the UMI correction afterwards.

Apart from the choice of the mapper, other decisions can influence the mapping results. One aspect is the choice of an appropriate annotation, which was shown to influence gene quantifications [13]. 10X Genomics recommends a filtered gene annotation that contains only a small subset that includes the biotypes protein coding, long non-coding RNA (lncRNA), and immunoglobulin and T-cell receptor genes. Other biotypes, e.g., pseudogenes are not included. Therefore, we were interested in whether a full annotation set affects the gene composition and the results of secondary analysis steps of scRNA-seq. Thus, we compared the mapping statistics of the filtered annotations to the complete (unfiltered) Ensembl annotation.

Specifically for scRNA-seq tools, comprehensive benchmarking articles are sparse [14]. Until now, only a limited number of benchmarking articles for scRNA-seq mappers have been published. Du et al. [15] conducted a benchmark between STAR and Kallisto on different scRNA-seq platforms and showed a higher accuracy and read mapping number with the STAR alignment. However, STAR has ∼4 times higher computation time and a 7-fold increase in memory consumption compared with Kallisto. Chen et al. and Vieth et al. performed a pipeline comparison with human and mouse *in vitro* and simulated datasets with a vast combination of tools concentrating on imputation, normalization, and calculation of differential expressions [16, 17]. Very recently, Booeshaghi and Pachter [18] published a preprint paper comparing Alevin and Kallisto on 10X datasets and stated that Alevin is significantly slower and requires more memory than Kallisto. As a direct answer to this preprint Zakeri et al. [19] showed opposing results by using identical reference genomes and adjusting the parameters to establish an equal configuration of the tools. In their preprint, they showed that Alevin is faster and requires less memory than Kallisto. In a third preprint the group from STARsolo performed a benchmark of STARsolo, Alevin, and Kallisto and claimed that STARsolo is more precise and outperforms the pseudo-alignment tools Alevin and Kallisto with simulated data. With a real dataset STARsolo replicated the results from Cell Ranger significantly faster while consuming much less memory [20].

These contradictory results show that an independent evaluation of all 5 alignment tools is needed. Therefore, we performed an in-depth and combined comparison of the 5 most common alignment tools (Cell Ranger 6, STARsolo, Alevin, Alevin-fry, and Kallisto) on different 10X datasets.

We used different scRNA-seq datasets of mouse and human to highlight specific differences and effects on downstream analysis with a focus on clustering, cell annotation, and differential gene expression analysis as prominent goals of droplet-based sequencing. Hereby, we followed the guidelines for reproducible, transparent, rigorous, and systematic benchmarking studies by Mangul et al. [21].

We are convinced that this benchmark of commonly used mappers is a valuable resource for other researchers to help them to choose the most appropriate mapper in their scRNA-seq analysis.

## Methods

### Datasets and reference genomes

#### 10X Drop-Seq data

We used 4 publicly available datasets.

#### Peripheral blood mononuclear cells

The first dataset is human peripheral blood mononuclear cells (PBMCs) from a healthy donor provided by 10X. It was downloaded from the 10X website [22]. It was sequenced with the v3 chemistry of the Chromium system from 10X.

#### Cardiac

The second dataset consists of 7 samples of mouse heart cells at individual time points (homeostasis and 1, 3, 5, 7, 14, or 28 days) after myocardial infarction [23]. Data were downloaded from the ArrayExpress database under the accession E-MTAB-7895. This dataset was sequenced with the v2 chemistry of the Chromium system from 10X.

#### Endothelial

The third dataset is from the mouse single-cell transcriptome atlas of murine endothelial cells from 11 tissues (n = 1) [24]. Data were downloaded from the ArrayExpress database under the accession E-MTAB-8077. This dataset was sequenced with the v2 chemistry of the Chromium system from 10X. The dataset cannot be mapped with Cell Ranger 4 and higher because the UMI sequence is 1 base shorter than is expected in the v2 chemistry (9 rather than 10 bases). To be able to map this dataset we added an A to all UMI sequences (R1 files) in the fastq file.

#### Heart failure

The fourth dataset contains 5 samples of patients with aortic stenosis. Single-nucleus sequencing was performed on tissue from the septum of the heart. The v3 chemistry from 10x Genomics was applied.

A technical summary of all datasets can be found in Supplementary Table S3 that contains the read composition and quality of each sample.

#### Gene annotation databases

Mouse and human genome and transcriptome sequences, as well as gene annotations, were downloaded from the Ensembl FTP server (Genome assembly GRCm38.p6 release 97 for mouse and GRCh38.p6 release 97 for human) [25]. The annotation for Cell Ranger 6 is the GENCODE version M22 for mouse and version 31 for human that match the Ensembl release 97 [26].

In this study, we compare 2 annotations (filtered and unfiltered). The filtered annotation file was generated applying the mkgtf and mkref function for Cell Ranger 6.0.2 according to the manual from 10X [27]. Therefore, the filtered annotation file contains the following features: protein coding, lncRNA, and the immunoglobulin and thyroid hormone receptor genes. For the unfiltered annotation, the complete Ensembl GTF file was used without any alterations.

### Software

#### Source Code

An index of the reference genome has been built for each tool individually, using the default parameters according to the manual pages of the individual tools. The exact commands for the creation of the indices and the mapping of the data are published at [28].

#### Cell filtering

Cells were filtered with the R package DropletUtils v1.6.1 [29]. All raw gene-count matrices were processed with the emptyDrops method [30]. The emptyDrops function applies the emptyDrops method, and 50,000 iterations of the Monte Carlo simulation were chosen to avoid low-resolution *P*-values due to a limited number of sampling rounds.

#### Downstream clustering analysis

Seurat v3.1.5 (SEURAT, RRID:SCR_007322) [31] was used for the downstream analysis. For all secondary analysis steps, we retained cells with a number of genes between 200 and 2,500 and a mitochondrial content <10%.

To compare the clustering we integrated the expression matrices of the samples from each mapper to remove technical noise and compare all combined samples. This was done for the Cardiac and PBMC datasets. The datasets were first normalized with the SCTransform function. We then ranked the features with the function SelectIntegrationFeatures and controlled the resulting features with the function PrepSCTIntegration. Anchors were determined by FindIntegrationAnchors and afterwards used with the IntegrateData function. The UMAP algorithm was run on the first 20 principal components of a PCA. To determine clusters, the FindClusters function was used with the parameter resolution = 0.15 to receive a number of clusters that is similar to the expected major cell types in the dataset. The Endothelial matrices were only merged and not integrated because the resulting clustering would not yield appropriate tissue clusters owing to the lack of different cell types. Yet, after merging the matrices we could obtain a similar clustering to the original study.

#### SCINA cluster comparison

To evaluate the effects of the different alignment and pseudo-alignment algorithms on clustering analysis, we created an artificial “ground truth,” where we assigned each barcode to a cell type. For this task we choose SCINA v1.2 [32] as an external classification tool. The semi-supervised classification method in SCINA requires a set of known marker genes for each cell type to be classified. Marker gene sets were obtained from Skelly et al. [33] and combined with other marker gene sets, as suggested by Tombor et al. [34] (Supplementary Table S4). An expectation–maximization (EM) algorithm uses the marker genes to obtain a probability for each provided cell type. After the classification each cell will be assigned a cell type that shows the highest probability based on the provided marker genes. Alignments with different mappers might result in different cell classifications for each barcode. Therefore, a consensus scheme is applied to each sample to create a cell type agreement for each barcode. Consensus of a cell classification for each barcode is achieved if ≥2 mappers agree on a cell type.

The remaining barcodes were used as a global barcode set for SCINA. Sankey plots were generated with the R package ggalluvial 0.12.3 [35] to illustrate the representation of cell types in each Seurat cluster (Supplementary Fig. S5). In addition, to convey the differences between SCINA and the Seurat clusters from each mapper, we calculated F1-score for the Cardiac dataset in Fig. 4A, as well as the precision, recall and F1-Score for the other datasets in Supplementary Fig. S6.

#### DEG analysis

For the differentially expressed genes (DEG) analysis each cluster from the integration in Seurat was assigned to a cell type by known marker genes for the PBMC dataset. The marker genes were obtained by the Seurat workflow for a similar 10X dataset [36]. DEGs were then calculated by using the FindAllMarkers function with the Wilcoxon-Rank-Sum test in Seurat and all DEGs above an adjusted *P*-value of 0.05 were removed. Upset plots were then created with the remaining DEGs (Fig. 4).

#### Additional software

The R-package ComplexHeatmap 2.6.2 (ComplexHeatmap, RRID:SCR_017270) [37] was used to create the Upset-plots (Figs 2, 4; Supplementary Fig. S2).

### Hardware

All computations were executed on a workstation with Intel Xeon E5-2667 CPU and 128 GB RAM. The OS was Ubuntu 18.04 LTS.

## Results

For the comparison of the 5 different alignment tools Cell Ranger 6, STARsolo, Alevin, Alevin-fry, and Kallisto, we analysed 4 representative datasets, which are denoted as PBMCs, Endothelial, Cardiac (Endothelial), and HF (see Methods section for a detailed description of the datasets) in the following.

### General statistics

**Figure 1:**
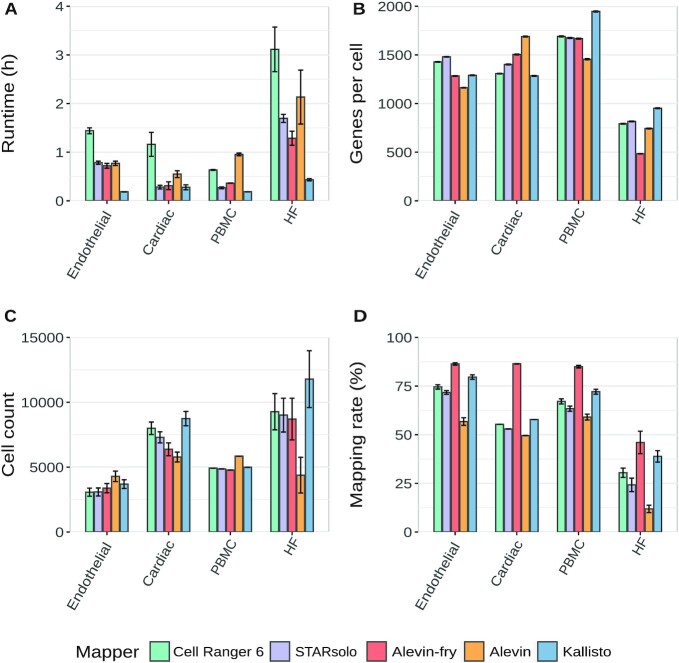
Summary of major measurements including runtime in hours (A), Genes per cell (B), cell count (C), and the mapping rate in percent (D). Bars and error bars indicate mean and SE, respectively

The overall performance and basic parameters such as runtime, genes per cell, cell number, and mapping rate are summarized in Fig. 1. In terms of runtime STARsolo, Alevin and Kallisto clearly outperformed Cell Ranger 6 and were ≥3 times faster. Kallisto showed the shortest runtimes and was on average 4–6 times faster than Cell Ranger 6. Additionally, Kallisto and Alevin-fry showed the highest transcriptome mapping rate whereas Alevin showed a slightly decreased mapping rate across all datasets. The cell count and the mean number of genes per cell were similar for Cell Ranger 6 and STARsolo across all datasets. Overall Cell Ranger and STARsolo had almost identical results regarding the cell count and the genes per cell, which is expected from the similarity of both tools. In contrast, Alevin and Kallisto showed different behavior for the genes per cell across the datasets. Compared to the other tools, Alevin detected more cells with fewer genes per cell in the PBMC and Endothelial datasets. However, it detected fewer cells with more genes per cell in the Cardiac, Endothelial, and HF datasets. This is caused by the initial whitelisting in Alevin. It calculates a knee point in which all barcodes above the knee point are considered as a putative whitelist. Barcodes below the knee point are then considered as erroneous barcodes. To correct these barcodes the algorithm tries to find a barcode in the putative whitelist by a substitution, insertion, or deletion. If this approach fails, the barcode is considered a noisy barcode and will be removed.

The percentage of noisy barcodes for Alevin is especially high for the HF and the Cardiac datasets. One possible explanation for this could be the library preparation protocol because these datasets are single-nucleus RNA-sequencing (snRNA-seq). The single-nucleus isolation protocol requires the extracellular matrix to be broken in order to release the nuclei. This leads to a higher amount of debris, which results in a higher percentage of background RNA contamination [38]. The percentage of barcodes that were discarded as “noisy barcodes” by Alevin are summarized for each sample in Supplementary Table S5.

We think that the knee point is higher than expected in the Cardiac and HF datasets and the correction fails on many barcodes, which, therefore, are removed prior to the mapping. More details with respect to these differences can be found in Supplementary Fig. S1. In the PBMC and the Endothelial datasets, Alevin shows small peaks in the lower left corner of the density plots for UMI counts and genes per cell. These peaks represent cells that have low UMI counts. For the Cardiac dataset Alevin did not detect these cells with low UMI content, which might explain the lower cell count for this dataset. However, in the Cardiac dataset, we observed more low-content cells for Kallisto. This is consistent with the finding that Kallisto detects most cells in the Cardiac dataset.

### Cell and gene identification

**Figure 2:**
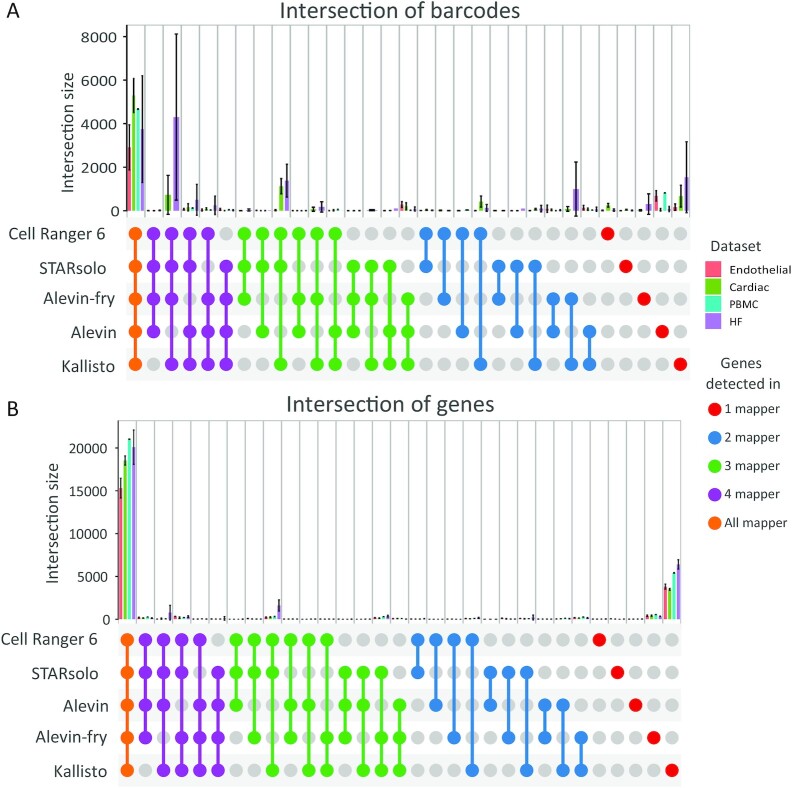
The barcodes (A) or genes (B) that have been detected by a certain number of mappers according to datasets. The number of mappers increases from right to left—frst the barcodes or genes that have only been detected by 1 mapper up to the barcodes or genes that have been detected in all tools.

In 10X droplet-based single-cell sequencing, the individual cells are usually identified via the randomized cell barcodes, which are predefined by the whitelist. To determine whether the different mapping tools detected identical cells, we merged the resulting cells based on their barcodes (Fig. 2A). The majority of barcodes were identified by all alignment tools. However, Cell Ranger 6, STARsolo, and Kallisto detected more barcodes as compared to Alevin and Alevin-fry in the Cardiac and HF datasets. These cells had far fewer reads per cell compared to the cells that were detected in all mappers, as shown in Panels 1 and 2 of Supplementary Fig. S2A and B. Alevin-fry and Kallisto also detected a set of barcodes. Their gene content is lower than the total dataset as can be seen in Panel 3 of Supplementary Fig. S2A and B. Similarly, Alevin detected unique barcodes for the PBMC and Endothelial datasets, which also had less gene content compared to the other cells detected by Alevin (Panel 4 of Supplementary Fig. S2A and B). Additionally, we recognized that the majority of these barcodes are not included in the whitelist from 10X (Supplementary Table S6). Panel 5 of Supplementary Fig. S2B shows the unique barcodes for Kallisto in the HF dataset, which also have less gene content than the other cells. Overall, we saw a reduced number of genes per cell for the barcodes that were only detected by 1 or 2 of the 5 alignment tools.

By comparing the expressed genes, we could show that all alignment tools detect a similar set of genes (Fig. 2B). Only Kallisto detected additional genes leading to a higher number of protein coding and lncRNA genes compared to the other tools (Supplementary Figure 3). In the HF dataset a small number of genes were not detected by Alevin-fry and Alevin.

**Figure 3:**
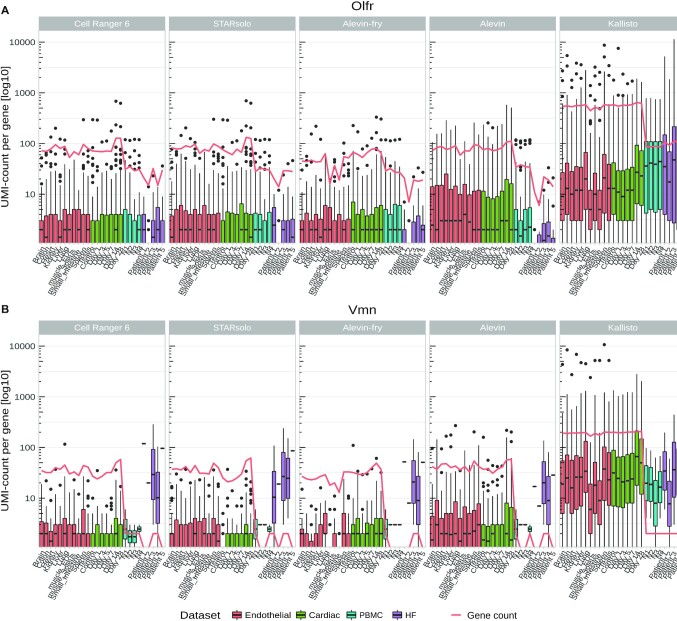
UMI counts of all detected (A) Vmn (Vomeronasal receptor genes) and (B) Olfr (Olfactory receptor genes) genes per mapper in each sample. The red line indicates the total number of expressed genes in the gene families. Boxes indicate the 25th and 75th percentiles and whiskers indicate maximal and minimal values.

One gene family that occurred more frequently in Kallisto is the Olfr (Olfactory receptor) gene family, which exhibits dramatically enriched UMI counts (Fig. 3A). Another Kallisto-enriched gene family is the Vmn (Vomeronasal receptors) family, which is detected with lower UMI counts compared to the Olfr family but is still elevated compared to the other tools (Fig. 3B). This leads to an increase in total gene counts for Kallisto (red line in Fig. 3) and an increase of the respective biotypes (Supplementary Fig. S3). The increased expression of genes from the Olfr gene family is exemplified in Supplementary Fig. 3. The HF dataset shows an increased UMI count of Vmn genes in only 2 or 3 samples. Vomeronasal genes are non-functional in humans because they were deactivated by mutations and therefore should not be expressed in human tissue [39].

### Effects on downstream analysis

To evaluate downstream effects of the different alignment tools, we performed a semi-supervised cell type assignment with SCINA. Therefore, we used all cells that were found by >2 mappers and assigned them to a corresponding cell type on the basis of the marker genes documented in Supplementary Table S2. Thereby, the majority of barcodes could be assigned to a specific cell type. Then we compared the clusters from each alignment tool to the assigned cell types from SCINA. Using the barcodes to identify each cell, we traced the cells from their respective clusters to the assigned cell type.

The fate from the predicted cell types to the clusters for each mapper can be observed in the sankey plots in Supplementary Fig. S5. Supplementary Fig. S6 provides metrics to further evaluate the detection of barcodes in each tool and cell type. Here, we used a greedy assignment of Seurat clusters with the cell type classification from SCINA. The cluster will be assigned with its highest abundance cell type. Then, precision, recall, and F1-scores were calculated.

In general, the clustering was similar when comparing the alignment tools. Minor differences were observed for Kallisto and Alevin. In the PBMC dataset, Kallisto showed a higher number of missing barcodes (M.b.), predominantly from monocytes. Missing barcodes are barcodes that were found in ≥2 of the other mappers but not in the present one, which means that these monocytes were not present or filtered out in Kallisto. This results in a lower recall in Supplementary Fig. S6B.

**Figure 4:**
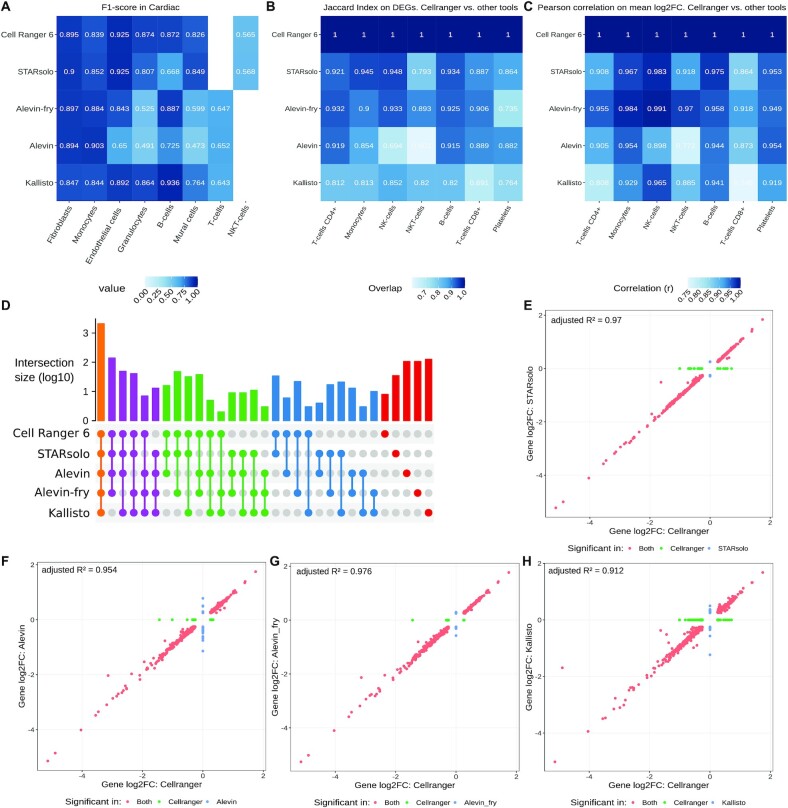
Accuracy of cell annotation in Seurat compared with the barcode consensus scheme from SCINA (A). Differential gene expression (DEGs) between Cell Ranger and the other tools as overlap (B) and correlation (C). Intersection that shows the detection of DEGs by a varying number of tools. The number of tools increases from right (DEGs that were detected by 1 tool) to left (DEGs that were detected by all tools) (D). The log_2_ fold change (log2FC) of DEGs CD4+ T cells between Cell Ranger and each of the other tools (E–H). The adjusted *R*² is the sample correlation of a linear model.

In the Cardiac dataset, the lower cell count found by Alevin leads to more barcodes associated with M.b.s, demonstrating that these cells are not detected in Alevin. The majority of these missing cells were assigned as endothelial cells, which means that in the Cardiac dataset Alevin detected only ∼50% of the endothelial cells that were found with the other tools. Also the number of B cells and granulocytes were decreased owing to the lower cell counts. This decrease is reflected in a lower recall in Supplementary Fig. S6D and a lower F1-score in Fig. 4A. However, the decrease in the latter cell types could not be confirmed in the PBMC dataset.

In summary, Cell Ranger 6 and STARsolo showed the highest agreement with the predicted cell types from SCINA, which is not surprising because they use the same internal algorithm. The overlaps of Alevin and Kallisto were lower owing to varying cell counts.

Analysis of the DEGs for the cell types of the PBMC dataset did show the highest agreement of STARsolo, Alevin-fry, and Cell Ranger. Major differences among the alignment tools are summarized in Fig. 4.

The accuracy of the barcode detection per tool in each cell type can be seen Fig. 4A. The highest accuracy can be seen in Cell Ranger, STARsolo, and Alevin. Lower accuracies are present in Alevin and Alevin-fry. Overall, cell types with a low amount of cells present in the dataset are difficult to detect in all tools. Comparing significant DEGs (*P*< 0.05) in PBMC, we see in Fig. 4A and B that STARsolo or Alevin has the highest overlap and correlation with Cell Ranger, respectively. Overall, Kallisto shows the lowest overlap and Alevin has intermediate overlaps. For the correlation (Fig. 4C) this ranking is not as clear because it highly depends on the cell type. Despite the differences most DEGs were detected by all tools in the PBMC dataset (Fig. 4D). Small groups of DEGs were detected by a single tool or when 1 or 2 tools have not detected DEGs. This is often the case in Alevin, Alevin-fry, and Kallisto. In Fig. 4E–H we compare significant DEGs (*P* < 0.05) from the T-cells CD4+ cell type of Cell Ranger against the other tools, similar to Kaminow et al. [20]. The highest correlation can be observed in STARsolo and Alevin-fry. Kallisto shows the lowest correlation against Cell Ranger and Alevin and intermediate correlation. These results are largely consistent with the results from Kaminow et al. [20]. The uniquely overrepresented genes in Kallisto are likely the OLFR and VMN genes that we showed in Fig. 3.

### Comparing filtered with unfiltered annotations

The default transcriptome annotation dataset, which is recommended for Cell Ranger 6 by 10X Genomics, misses some important biotypes like pseudogenes and TECs (sequences that indicate protein-coding genes that need to be experimentally confirmed). These differences in gene model compositions can have profound effects on the read mapping and the gene quantification as reported by Zhao and Zhang [13]. To evaluate the effects of different annotation sets on 10x scRNA-seq data, we compared the mapping statistics of the filtered annotations to the complete (unfiltered) Ensembl annotation.

Besides the increase of processed pseudogenes (Supplementary Fig. S3), the use of the unfiltered annotation led to a decrease in mitochondrial (MT) content across all alignment tools as shown in Supplementary Fig. S7A. Especially the 2 mouse datasets showed a strong reduction of MT content in the unfiltered annotation. Supplementary Fig. S7B shows the amount of reads per mitochondrial gene, which are not mapped. Further investigation revealed that the unfiltered annotation includes pseudogenes that are identical to MT genes (Supplementary Fig. S7E). A potential explanation for the reduced MT content with the unfiltered annotation is that the mapping algorithms cannot uniquely assign a read to the MT gene because the read can simultaneously map to the MT gene and the identical pseudogene (Supplementary Fig. S7D and E). Therefore, this read is discarded. Because high MT content is a sign of damaged or broken cells, cells with an MT content above a certain threshold are usually filtered out. However owing to the reduced MT content fewer cells surpassed the MT content threshold and we could retrieve more cells. These additional cells clustered along with the other cell types, indicating that the cell quality is good and that these additional cells are not broken or damaged cells as exemplified in Supplementary Fig. S7C. Using the unfiltered annotation yielded up to 10% more cells per sample. However deeper research is required to ensure the quality of these additional cells.

## Discussion

Because handling of scRNA-seq data is a moving target, the constant revision of new tools is important to ensure reliable results. Therefore, independent benchmarking and evaluation of uncertainties of analysis tools is of central importance [40].

Our study of real 10X Genomics datasets demonstrated advantages and disadvantages of 5 popular scRNA-seq mappers for gene quantification in single cells and adds to the growing number of benchmarks. The tools benchmarked in this study are widely used in many laboratories; thus, our results are relevant for many scientists working with scRNA-seq data. All mappers have been evaluated on *in vivo* datasets because these data might reveal unexpected differences or characteristics that probably could not have been found with simulated data as is highlighted by Srivastava et al. [41]. From our perspective, the only advantage of using simulated datasets is that it allows the assessment of read accuracy, which has already been done for the mappers we used in this study [21, 42, 43].

The runtime is one of the most important factors when choosing a tool, but the quality of the results is of equal importance. In our detailed analysis, we show that Cell Ranger 6 could be easily replaced with STARsolo because they show almost identical results but STARsolo is up to 5× faster in comparison with Cell Ranger 6. The low variance in the PBMC dataset for the cell counts and genes per cell for Cell Ranger 6 and STARsolo can be explained by the predefined sample size by 10X. With the option for selective alignment, which was used throughout this article, Alevin-fry had a similar runtime to STARsolo. However Kaminow et al. showed that the runtime decreases when using the pseudo-alignment algorithm (sketch mode) for Alevin-fry, yet this leads to a reduction in accuracy [20] as mapping positions are not validated via alignment scoring [7].

Du et al. 2020 [15] reported that Kallisto was even faster than STARsolo, a finding that is consistent with our results because Kallisto had overall the shortest runtime across all mappers. However, the number of cells and the genes per cell varied across datasets for Alevin and Kallisto.

Additionally, Kallisto seems to detect genes of the Vmn and Olfr family as highly expressed in several single-cell datasets, although these genes are typically not expressed in these tissues. Because these gene families belong to the group of sense and smell receptors, they are expected to be expressed at lower levels or be absent in PBMCs and heart tissue and likely represent artefacts. We consistently show that these genes are overrepresented in the Kallisto results (Fig. 3 and Supplementary Fig. S4). Because Kallisto does not perform quality filtering for UMIs, this might have influenced the reported number of genes per cell as is indicated by Parekh et al. [44].

Another major difference of the tested mapping tools is the handling of errors in the barcodes. We could show that Alevin often detects unique barcodes, which were not identified by the other tools. These barcodes had very low UMI content and were not listed in the 10X whitelist. Therefore, It can be assumed that these barcodes were poorly assigned (Section 4 of Supplementary Fig. S2). A possible explanation might be the use of a putative whitelist in Alevin that was calculated prior to the mapping, instead of using the one provided by 10X. In Alevin-fry the barcode correction seems to be improved because there is no severe enrichment of cells that are unique to Alevin-fry.

While comparing the resulting cell clusters generated by each tool, we recognized only minor differences between the tools. Especially the clusters from Cell Rranger and STARsolo were similar. However, Kallisto detected fewer monocytes in the PBMC dataset and Alevin detected fewer endothelial cells in the Cardiac dataset. Overall, we saw a much higher variance in the clustering in the Cardiac dataset. This could be due to the use of an older version of the library extraction protocol (10X v2), which has short barcode and UMI sequences, or a lower sequencing quality of the Cardiac dataset.

The comparison of the complete annotation from Ensembl and the filtered annotation, as suggested by 10X, revealed that multi-mapped reads play an important role in scRNA-seq analysis. In this study, we showed that using an unfiltered annotation reduces the MT content of cells compared to the filtered annotation. Therefore, the MT content as a way to distinguish valid cells and dead or damaged cells has to be carefully conducted because it depends on the annotation. The recommended annotation from 10X, which only contains genes with the biotypes protein-coding gene and long non-coding gene, might lead to an overestimation of MT gene expression. However, on the other side all of these genomic loci that are identical to MT genes, so-called nuclear mitochondrial DNA (NUMT), are unprocessed pseudogenes and are not yet experimentally validated and could well be artefacts from the genome assembly. For human samples we could not see major differences in the downstream results while using the complete annotation; therefore it might well be used instead of the filtered annotation. However for mouse samples a clear recommendation of whether to use the filtered or the complete annotation cannot be made because more research into this issue is required. These results suggest that there is still a need to improve the handling of multi-mapped reads in scRNA-seq data. In datasets with a high percentage of multi-mapped reads, EM-like algorithms, as suggested by Srivastava et al. [45], can be advantageous and improve gene quantification in scRNA-Seq datasets. Future mapping tools might, e.g., consider the likelihood of a gene to be expressed in a certain cell type. This might enhance the quantification of cell type–specific genes and prevent multi-mapped reads for cell types, where a certain gene is rarely expressed. Inclusion of mapping uncertainties may be another fruitful direction.

Srivastava et al. [41] observed that there are significant differences between methods that align against the transcriptome with quasi-mapping (e.g., Alevin) and methods that do full spliced alignments against the genome (e.g., STAR) [41]. The observed discrepancies, when using the filtered annotation in our experiments, often result from genes that share the same sequences, and therefore, the true alignment origin cannot be determined. The reported positions of reads contained annotated transcripts, e.g., from the mitochondria and a few unprocessed pseudogenes.

**Figure 5:**
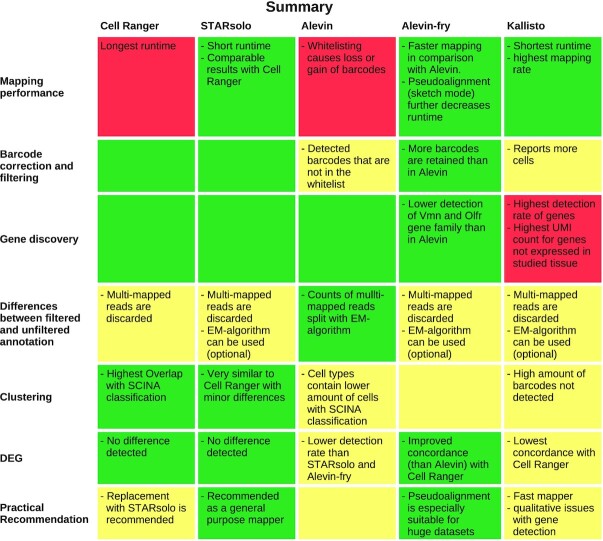
Summary of the results for each evaluated section of interest and mapper. Good results are coloured in green, intermediate in yellow, and poor results in red.

In conclusion, our analysis shows that Alevin, Kallisto, and STARsolo are fast and reliable alternatives to Cell Ranger 6. They also scale to large datasets. A summary of advantages and disadvantages of each individual tool is provided in Fig. 5.

In general, we could show that STARsolo is an ideal substitute for Cell Ranger 6 because it is faster but otherwise performs similarly. If high-quality cell counts need to be obtained, Alevin-fry appears to be the most suitable method because mean gene counts are high and poor-quality barcodes are seldom reported. Kallisto, while reporting the highest number of barcodes, also contains many barcodes that could not be assigned to cells expected in the heart on the basis of known marker genes. Therefore, we generally recommend STARsolo or Alevin-fry for most end-users as an alternative to Cell Ranger because these tools' performance was very stable over all datasets. For very large projects with a high number of samples, pseudo-alignment tools such as Kallisto can be advantageous in terms of runtime and storage efficiency, at the cost of a slight reduction in accuracy.

## Data Availability

All supporting data and materials are available in the *GigaScience* GigaDB database [46].

## Availability of Source Code and Requirements

Project name: Comparative Analysis of common alignment tools for single-cell RNA sequencing

Project home page: https://github.com/rahmsen/BenchmarkAlignment

Operating system: x86_64-pc-linux-gnu (64-bit)

Programming language: R (version 3.6.2)

Other requirements: Cell Ranger 6.0, STARsolo 2.7.4a, Salmon 1.5.1, Alevin 1.1.0, Alevin-fry 0.4.0, Kallisto 0.46.1, Seurat 4.0.3, DropletUtils 1.6.1, SCINA v1.2, ggalluvial 0.12.3, ComplexHeatmap 2.6.2, reshape2 1.4.4, ggplot 3.3.5, ggpubr 0.4.0, dplyr 1.0.7, svglite 2.0.0, jsonlite 1.7.2, egg 0.4.5

License: MIT

## Supplementary Material

giac001_GIGA-D-21-00129_Original_Submission

giac001_GIGA-D-21-00129_Revision_1

giac001_GIGA-D-21-00129_Revision_2

giac001_GIGA-D-21-00129_Revision_3

giac001_Response_to_Reviewer_Comments_Revision_2

giac001_Reviewer_1_Report_Original_Submission Bo Li, Ph.D. -- 6/7/2021 Reviewed

giac001_Reviewer_1_Report_Revision_1 Bo Li, Ph.D. -- 10/25/2021 Reviewed

giac001_Reviewer_2_Report_Original_Submission Serghei Mangul -- 6/12/2021 Reviewed

giac001_Reviewer_2_Report_Revision_1 Serghei Mangul -- 10/26/2021 Reviewed

giac001_Reviewer_3_Report_Original_Submission Hirak Sarkar -- 7/7/2021 Reviewed

giac001_Reviewer_3_Report_Revision_1 Hirak Sarkar -- 10/27/2021 Reviewed

giac001_Reviewer_3_Report_Revision_2 Hirak Sarkar -- 11/29/2021 Reviewed

giac001_Supplemental_Files

## Additional Files

**Supplementary Figure S1**: Distribution of UMI-counts and genes per cell for the individual datasets. Distribution is a kernel density estimate with a Gaussian kernel of all samples for the PBMC, Endothelial, and Cardiac datasets. The left column displays the UMI counts per cell, and the right column, the number of genes per cell.

**Supplementary Figure S2**: (A) Amount of common and unique barcodes (mean ± s.e.m.) detected by the individual alignment tools. Intersections of interest are marked by numbers. (B) Gaussian distribution of genes per cells for the interesting intersection and dataset from A. The distributions of the tools from the intersection (non-transparent) are compared with all detected barcodes of each tool (transparent lines [in the background]; denoted with asterisk in the legend).

**Supplementary Figure S3**: Number (mean + s.e.m.) of biotypes per dataset with ≥1 UMI count after mapping with a filtered (solid dots) or unfiltered annotation (triangles in squares). IG = Immunoglobulin genes, TR = T-cell receptor genes, TEC = sequences that need to be experimentally confirmed.

**Supplementary Figure S4**: Expression of the OLFR gene family per cell in the PBMC dataset for (A) Cell Ranger, (B) Cell Ranger 6, (C) STARsolo, (D) Alevin, and (E) Kallisto. Cells are sorted by clusters that are denoted by the colour code above each heat map.

**Supplementary Figure S5**: Sankey plots demonstrating the fate of each cell from SCINA cell types to the clusters obtained by Seurat. Cells were kept only if >2 mappers detected a barcode. (A) PBMC dataset; (B) Cardiac dataset. M.b.: missing barcodes. These are barcodes that were found in ≥2 of the other mappers but not in the present one.

**Supplementary Figure S6**: Consistency of cells detected by each mapper (“ground truth”) by greedy assignment of the barcodes to the SCINA classification. (A) F1-Score, (B) recall, and (C) precision for the PBMC dataset. The recall (D) and precision (E) for the Cardiac dataset.

**Supplementary Figure S7**: Difference in mitochondrial content (MT content) of cells due to use of a filtered and unfiltered annotation. (A) MT content of cells separated by filtered and unfiltered annotation. (B) Reads mapped to the mitochondrial genes for the PBMC and Rosenthal dataset with unfiltered annotation. Orange indicates the amount of reads that are removed due to multimapping when an unfiltered annotation is used. (C) UMAP showing cells in green that are retained because the MT content is below the filtering threshold when the unfiltered annotation was used in the mapping. (D) Mitochondrial genes and its closest pseudogene when the mappers reported the secondary mapping position along with the sequence similarity to the MT gene. (E) Example of the mapping process of a read from an MT gene with a filtered/unfiltered annotation. Because the filtered annotation does not include potential NUMTs, the read is uniquely mapped to the MT gene. Whereas the complete set contains NUMTs and therefore the read cannot be uniquely mapped to the MT genes (multi-mapped) and therefore is discarded.

**Supplementary Table S1**: Distribution of UMI-counts and genes.

**Supplementary Table S2**: Common and unique barcodes detected per mapper.

**Supplementary Table S3**: Expressed biotypes per mapper.

**Supplementary Table S4**: Heatmap of OLFR genes.

**Supplementary Table S5**: Sankey plot comparisson for each mapper to SCINA annotation.

**Supplementary Table S6**: Heatmap of recall and precission rate.

## Abbreviations

CPU: central processing unit; DEG: differentially expressed genes; GTF: General Feature Format; lncRNA: long non-coding RNA; M.b.: missing barcodes; MM: mismatch; MT: mitochondrial; NUMT: nuclear mitochondrial DNA; Olfr: Olfactory receptor; PBMC: peripheral blood mononuclear cell; PCA: principal component analysis; SCINA: Semi-Supervised Subtyping Algorithm; scRNA-seq: single-cell RNA sequencing; STAR: Spliced Transcripts Alignment to a Reference; UMAP: Uniform Manifold Approximation and Projection; UMI: unique molecular identifier; Vmn: vomeronasal receptor.
