# Supplementary material for: Comparative analysis of common alignment tools for single-cell RNA sequencing
Source: Gigascience. 2022 Jan 27;11:giac001. doi: 10.1093/gigascience/giac001 (PMC8848315; doi:10.1093/gigascience/giac001)
Supplement: giac001_GIGA-D-21-00129_Revision_3 [file giac001_giga-d-21-00129_revision_3.pdf]

GigaScience

Comparative Analysis of common alignment tools for single cell RNA sequencing

--Manuscript Draft--

**Manuscript Number:**	GIGA-D-21-00129R3
**Full Title:**	Comparative Analysis of common alignment tools for single cell RNA sequencing
**Article Type:**	Research
**Funding Information:**	German Center for Cardiovascular Research (DZHK) — Prof. Dr. Stefanie Dimmeler
**Abstract:**	Background : With the rise of single cell RNA sequencing new bioinformatic tools have been developed to handle specific demands, such as quantifying unique molecular identifiers and correcting cell barcodes. Here, we benchmarked several datasets with the most common alignment tools for scRNA-seq data. We evaluated differences in the whitelisting, gene quantification, overall performance and potential variations in clustering or detection of differentially expressed genes.<br>We compared the tools Cell Ranger 6, STARsolo, Kallisto and Alevin on three published datasets for human and mouse, sequenced with different versions of the 10X sequencing protocol.<br>Results : Striking differences have been observed in the overall runtime of the mappers. Besides that Kallisto and Alevin showed variances in the number of valid cells and detected genes per cell. Kallisto reported the highest number of cells, however, we observed an overrepresentation of cells with low gene content and unknown cell type. Conversely, Alevin rarely reported such low content cells.<br>Further variations were detected in the set of expressed genes. While STARsolo, Cell Ranger 6, Alevin-fry and Alevin released similar gene sets, Kallisto detected additional genes from the Vmn and Olfr gene family, which are likely mapping artifacts. We also observed differences in the mitochondrial content of the resulting cells when comparing a prefiltered annotation set to the full annotation set that includes pseudogenes and other biotypes.<br>Conclusion : Overall, this study provides a detailed comparison of common scRNA-seq mappers and shows their specific properties on 10X Genomics data.
**Corresponding Author:**	David John, Ph-D<br>Goethe-Universitat Frankfurt am Main<br>Frankfurt am Main, Hessen GERMANY
**Corresponding Author Secondary Information:**	
**Corresponding Author's Institution:**	Goethe-Universitat Frankfurt am Main
**Corresponding Author's Secondary Institution:**	
**First Author:**	Ralf Schulze-Bruening, M.Sc
**First Author Secondary Information:**	
**Order of Authors:**	Ralf Schulze-Bruening, M.Sc
	Lukas S. Tombor, M.Sc.
	Marcel H. Schulz, Professor
	Stefanie Dimmeler, Professor
	David John, Ph-D
**Order of Authors Secondary Information:**	
**Response to Reviewers:**	Responds on Reviewer3<br><br>Reviewer 3<br><br>1.) I have one more comment about this version of the manuscript. I think that since

	alevin exposes multiple ways to run the alignment phase upstream of alevin-fry, including the sketch mode, a more apt performance comparison is either to include the alevin-fry pipeline in sketch mode, or if this isn't done, to make clear in the manuscript that this configuration is not included.<br><br>I would like to refer authors to Figure 6A of STAR-solo manuscript (https://www.biorxiv.org/content/10.1101/2021.05.05.442755v1.full.pdf) which exposes such a comparison, and which finds alevin-fry with sketch based alignment to be the fastest configuration overall. As such, drawing conclusion about the speed of the tools presented, without mentioning that certain configurations that optimize for speed have not been included, might be misleading to general readers who are not otherwise aware of the configurability of these tools.<br><br>We thank the reviewer for this valuable comment. As suggested we stated in the discussion section that the Alevin sketch mode was not used for the mapping processes performed with Alevin-fry. We changed Figure 5 accordingly and added the following sentence to the discussion section of the manuscript:<br><br>"With the option for selective alignment, which was used throughout this paper, Alevin-fry had a similar runtime than STARsolo. However Kaminov et. al. showed that the runtime decreases when using the pseudo-alignment algorithm (sketch mode) for Alevin-fry, yet this leads to a reduction in accuracy [https://www.biorxiv.org/content/10.1101/2021.05.05.442755v1.full.pdf] as mapping positions are not validated via alignment scoring [https://www.biorxiv.org/content/biorxiv/early/2021/08/26/2021.06.29.450377.full.pdf]."
**Additional Information:**	
**Question**	**Response**
Are you submitting this manuscript to a special series or article collection?	No
**Experimental design and statistics**<br><br>Full details of the experimental design and statistical methods used should be given in the Methods section, as detailed in our Minimum Standards Reporting Checklist. Information essential to interpreting the data presented should be made available in the figure legends.<br><br>Have you included all the information requested in your manuscript?	Yes
**Resources**<br><br>A description of all resources used, including antibodies, cell lines, animals and software tools, with enough information to allow them to be uniquely identified, should be included in the Methods section. Authors are strongly encouraged to cite Research Resource	Yes

Powered by Editorial Manager® and ProduXion Manager® from Aries Systems Corporation

Identifiers (RRIDs) for antibodies, model organisms and tools, where possible.<br><br>Have you included the information requested as detailed in our Minimum Standards Reporting Checklist?	
**Availability of data and materials**<br><br>All datasets and code on which the conclusions of the paper rely must be either included in your submission or deposited in publicly available repositories (where available and ethically appropriate), referencing such data using a unique identifier in the references and in the "Availability of Data and Materials" section of your manuscript.<br><br>Have you have met the above requirement as detailed in our Minimum Standards Reporting Checklist?	Yes

Manuscript Click here to access/download;Manuscript;Manuscript_DJ.docx

Comparative Analysis of common alignment tools for single cell RNA sequencing

Ralf Schulze Brüning[1,3], Lukas Tombor[1,2], Marcel H. Schulz[1,2,3], Stefanie Dimmeler[1,2,3], David John[1,3]

1.) Institute of Cardiovascular Regeneration, Theodor-Stern-Kai 7, 60590 Frankfurt

2.) German Center for Cardiovascular Research (DZHK); Frankfurt; Germany

3.) Cardio-Pulmonary Institute (CPI), Frankfurt; Germany

Word counts: 8307

Corresponding author:

David John

Institute for Cardiovascular Regeneration

Centre of Molecular Medicine

Goethe University Frankfurt

Theodor-Stern-Kai 7

60590 Frankfurt; Germany

john@med.uni-frankfurt.de

ORCIDs:

Lukas S. Tombor [0000-0002-5867-576X]

Marcel H. Schulz [0000-0002-1252-3656]

Stefanie Dimmeler [0000-0002-1045-2436]

David John [0000-0003-3217-5449]

Keywords: Benchmarking, scRNA-seq, Mapping-Algorithms, Aligners, Transcriptomics, Mappers

Abstract

Background: With the rise of single cell RNA sequencing new bioinformatic tools have been developed to handle specific demands, such as quantifying unique molecular identifiers and correcting cell barcodes. Here, we benchmarked several datasets with the most common alignment tools for scRNA-seq data. We evaluated differences in the whitelisting, gene quantification, overall performance and potential variations in clustering or detection of differentially expressed genes.

We compared the tools Cell Ranger version 6, STARsolo, Kallisto, Alevin and Alevin-fry on three published datasets for human and mouse, sequenced with different versions of the 10X sequencing protocol.

Results: Striking differences have been observed in the overall runtime of the mappers. Besides that Kallisto and Alevin showed variances in the number of valid cells and detected genes per cell. Kallisto reported the highest number of cells, however, we observed an overrepresentation of cells with low gene content and unknown cell type. Conversely, Alevin rarely reported such low content cells. Further variations were detected in the set of expressed genes. While STARsolo, Cell Ranger 6, Alevin-fry and Alevin produced similar gene sets, Kallisto detected additional genes from the Vmn and Olfr gene family, which are likely mapping artifacts. We also observed differences in the mitochondrial content of the resulting cells when comparing a prefiltered annotation set to the full annotation set that includes pseudogenes and other biotypes.

Conclusion: Overall, this study provides a detailed comparison of common scRNA-seq mappers and shows their specific properties on 10X Genomics data.

Background

Major advances could be achieved in the transcriptomics field by using single cell RNA sequencing (scRNA-seq) to conduct differential expression analysis, clustering, cell type annotation and pseudotime analysis on a single cell level [1]. Analysis of scRNA-seq data helped to reveal new insights into cellular heterogeneity, e.g. the altered phenotypes in circulating immune cells of patients with chronic ischemic heart diseases [2] or the transcriptional diversity of aging fibroblasts [3]. However, the analysis of scRNA-seq data is resource intensive and requires deeper knowledge of specific characteristics of each analysis tool. The most resource intensive step during single cell NGS data analysis is the alignment of reads to a reference genome and/or transcriptome. Therefore, a common question relates to the choice of the best scRNA-seq alignment tool that can be incorporated into a fast, reliable and reproductive analysis pipeline. Here we evaluated five popular alignment tools Cell Ranger 6, STARsolo as well as the pseudo-alignment tools Alevin, Alevin-fry and Kallisto.

Technological properties of these mappers are summarized in Supplementary table 1. In general, the Cell Ranger 6 software suite developed for 10X Genomics Chromium platform [4] data uses STAR [5] as the standard alignment tool. STAR, originally designed for bulk-seq data, performs a classical alignment approach by utilizing a maximal mappable seed search, thereby all possible positions of the reads

can be determined. In contrast, Kallisto [6], Alevin-fry [7] and Alevin [8] perform an alignment-free approach, so called pseudo-alignment.

The idea of alignment-free RNA-Seq quantification was introduced by Patro et al. [9] with Salfish and promised much faster alignments. Here, k-mers of reads and the transcriptome are compared, and no complete alignment between read and reference is computed, which leads to huge speed-ups. Two years later, the Patcher lab introduced Kallisto, a pseudo-alignment algorithm that achieved similar improvements in runtime but with higher alignment accuracy compared to Salfish. In response, Patro et. al. published Salmon [10], a reimplementation of their initial Salfish tool which implements a samples-specific bias model that accounts for various biases that prevent high false positive rates and overall refined expression estimates. With the advent of scRNA-seq, Kallisto introduced the Kalisto-bustools pipeline and Alevin was released as an extension of Salmon to process scRNA-seq data.

Alevin makes use of an improved pseudo-alignment called selective alignment that promises a higher specificity but an increase in runtime compared to its previous implementation. With the release of Alevin-fry, Alevin introduced a custom version of pseudo-alignment that can use a memory-efficient sketch data structure to improve processing speed of large datasets. However, it has been shown that pseudo-alignment tools have limitations in the quantification of lowly expressed genes [11].

In contrast to bulk-RNA-seq, preprocessing of scRNA-seq requires specific features. Essential features are cell calling, removing PCR duplicates and assigning reads to individual genes and cells. These features can be achieved through barcode and UMI sequences, which are sequenced along with the reads. Therefore, the correct

handling of barcode and UMI sequences are crucial steps while processing scRNA-seq data. Each alignment tool applies different strategies to handle these errors.
The most important step for cell calling is the correction of sequencing errors within the barcodes. Cell Ranger 6, STARsolo and Kallisto correct barcodes by comparing the sequenced barcodes to a set of all barcodes that are included in the library preparation kit, the so-called whitelist. This whitelist is provided by 10X Genomics. If no exact match of a sequenced barcode can be found in the whitelist, this barcode is replaced with the closest barcode from the whitelist, if the Hamming distance is not bigger than 1. Alevin, however, generates a putative whitelist of highly abundant barcodes that exceed a previously defined knee point. Afterwards Alevin assigns error prone barcodes to the closest barcode from the putative whitelist, while allowing an edit distance of 1.
In order to remove biases from PCR duplicates (reads with the same mapping position, the same cell barcode) an identical unique molecular identifier (UMI) sequence is required for pooling these PCR duplicates. To correct errors in UMI sequences, Cell Ranger 6 and STARsolo group reads according to their barcode, UMI and gene annotation, while allowing 1 mismatch (MM) in the UMI sequence. As error prone UMIs are rare, they will be replaced by the higher abundant (supposedly correct) UMI. Afterwards a second round is done by grouping the barcode, corrected UMI and gene annotation. When groups differ only by their gene annotation, the group with the highest read count is kept for UMI counting. The other groups are discarded, as these reads origin from the same RNA construct but were mapped to different genes. A detailed description of the whitelisting and UMI correction methods, which are unique for Cell Ranger, can be found on the 10X website [12].
Alevin builds a UMI graph and tries to find a minimal set of transcripts for UMI

deduplication [8]. In this process, similar UMIs are corrected. Kallisto applies a naive collapsing method which removes reads that originate from different molecules but contain the same UMI [6].
The third important preprocessing step of scRNA-seq data is the assignment of reads to individual genes and cells. Here, the alignment tools have striking differences handling these multi mapped reads. In STARsolo, Cell Ranger 6 and Kallisto multi-mapped reads are discarded when no unique mapping position can be found within the genome/transcriptome. Whereas Alevin equally divides the counts of a multi mapped read to all potential mapping positions. The order of necessary steps for quantification i.e. the alignment and barcode and UMI correction can vary for each tool. Therefore, Suppl Table 2 shows this order. Kallisto has the most different order where the barcode correction is executed after the alignment and a UMI correction is not performed. The other tools perform the barcode correction before the alignment and the UMI correction afterwards.

Apart from the choice of the mapper, other decisions can influence the mapping results. One aspect is the choice of an appropriate annotation, which was shown to influence gene quantifications [13]. 10X Genomics recommends a filtered gene annotation that contains only a small subset that includes the biotypes protein coding, lncRNA and Immunoglobulin and T-cell receptor genes. Other biotypes e.g. pseudogenes are not included. Therefore, we were interested if a full annotation set affects the gene composition and the results of secondary analysis steps of scRNA-seq. Thus, we compared the mapping statistics of the filtered annotations to the complete (unfiltered) Ensembl annotation.

Specifically for scRNA-Seq tools, comprehensive benchmarking papers are sparse [14]. Until now, only a limited number of benchmarking papers for scRNA-seq mappers were published. Du et al. [15] conducted a benchmark between STAR and Kallisto on different scRNA-seq platforms and showed a higher accuracy and read mapping number with the STAR alignment. However, STAR has about 4 times higher computation time and 7 fold increase in memory consumption than Kallisto. Chen et al. and Vieth et al. performed a pipeline comparison with human and mouse in vitro and simulated datasets with a vast combination of tools concentrating on imputation, normalization and calculation of differential expression [16,17]. Very recently, Booeshaghi and Pachter [18] published a preprint paper comparing Alevin and Kallisto on 10X datasets and stated that Alevin is significantly slower and requires more memory than Kallisto. As a direct answer to this preprint Zakeri and Patro [19] showed opposing results by using identical reference genomes and adjusting the parameters to establish an equal configuration of the tools. In their preprint, they showed that Alevin is faster and requires less memory than Kallisto. In a third preprint the group from STARsolo performed a benchmark of STARsolo, Alevin and Kallisto and claimed that STARsolo is more precise and outperforms the pseudo-alignment tools Alevin and Kallisto with simulated data. With a real dataset STARsolo replicated the results from Cell Ranger significantly faster, while consuming much less memory [20].

These contradictory results show that an independent evaluation of all five alignment tools is needed urgently. Therefore, we performed an in-depth and combined comparison of the five most common alignment tools (Cell Ranger 6, STARsolo, Alevin, Alevin-fry and Kallisto) on different 10X datasets.

We used different scRNA-seq data sets of mouse and human to highlight specific differences and effects on downstream analysis with a focus on clustering, cell annotation, differentially gene expression analysis as prominent goals of droplet-based sequencing. Hereby, we followed the guidelines for reproducible, transparent, rigorous and systematic benchmarking studies by Mangul et.al [21] .

We are convinced that this benchmark of commonly used mappers is a valuable resource for other researchers to help them to choose the most appropriate mapper in their scRNA-seq analysis.

Methods

Datasets and Reference Genomes

10X Drop-Seq Data

We used four publicly available data sets.

PBMC

The first data set is human Peripheral blood mononuclear cells (PBMCs) from a healthy donor provided by 10X. It was downloaded from the 10X website [22]. It was sequenced with the v3 chemistry of the Chromium system from 10X.

Cardiac

The second data set consists of 7 samples of mouse heart cells at individual timepoints (Homeostasis, 1 day, 3 days, 5 days, 7, days, 14 days, 28 days) after myocardial infarction [23]. Data was downloaded from the ArrayExpress database

under the accession E-MTAB-7895. This dataset was sequenced with the v2 chemistry of the Chromium system from 10X.

Endothelial

The third dataset is from the mouse single cell transcriptome atlas of murine endothelial cells from 11 tissues (n=1) [24]. Data was downloaded from the ArrayExpress database under the accession E-MTAB-8077. It was sequenced with the v2 chemistry of the Chromium system from 10X. The dataset can not be mapped with Cell Ranger 4 and higher because the UMI sequence is one base shorter than is expected in the v2 chemistry (9 than 10 bases). To be able to map this dataset we added an A to all UMI sequences (R1 files) in the fastq file.

Heart Failure (HF)

The fourth dataset contains five samples of patients with aortic stenosis. Single nuclei sequencing was performed on tissue from the septum of the heart. The v3 chemistry from 10x Genomics was applied.

A technical summary of all datasets can be found in Suppl. Table 3 that contains the read composition and quality of each sample.

Gene annotation databases

Mouse and human genome and transcriptome sequences as well as gene annotations were downloaded from the Ensembl FTP server (Genome assembly GRCm38.p6 release 97 for mouse and GRCh38.p6 release 97 for human) [25]. The

annotation for Cell Ranger 6 is the GENCODE version M22 for mouse and version 31 for human that match the Ensembl release 97 [26].

In this study, we compare two annotations (filtered and unfiltered). The filtered annotation file was generated applying the *mkgtf* and *mkref* function for Cell Ranger 6.0.2 according to the manual from 10X [27]. Therefore, the filtered annotation file contains the following features: protein coding, lncRNA and the immunoglobulin and thyroid hormone receptor genes. For the unfiltered annotation, the complete Ensembl GTF file was used without any alterations.

Software

Source Code

An index of the reference genome has been built for each tool individually, using the default parameters according to the manual pages of the individual tools. The exact commands for the creation of the indices and the mapping of the data are published at [28].

Cell filtering

Cells were filtered with the R package DropletUtils v1.6.1 [29]. All raw gene-count matrices were processed with the emptyDrops method [30]. The *emptyDrops* function applies the emptyDrops method and 50000 iterations of the Monte Carlo simulation were chosen, to avoid low resolution p-values due to a limited number of sampling rounds.

Downstream clustering analysis

Seurat v3.1.5 (SEURAT, RRID:SCR_007322) [31] was used for the downstream analysis. For all secondary analysis steps, we retained cells with a number of genes between 200 and 2500 and a mitochondrial content < 10%.

To compare the clustering we integrated the expression matrices of the samples from each mapper to remove technical noise and compare all combined samples. This was done for the Cardiac and PBMC data set. The data sets were first normalized with the *SCTransform* function. We then ranked the features with the function *SelectIntegrationFeatures* and controlled the resulting features with the function *PrepSCTIntegration*. Anchors were determined by *FindIntegrationAnchors* and afterwards used with the *IntegrateData* function. The UMAP algorithm was run on the first 20 principal components of a PCA. To determine clusters, the *FindClusters* function was utilized with the parameter *resolution*=0.15 to receive a number of clusters that is similar to the expected major cell types in the data set. The Endothelial matrices were only merged and not integrated because the resulting clustering would not yield appropriate tissue clusters due to the lack of different cell types. Yet, after merging the matrices we could obtain a similar clustering to the original study.

SCINA cluster comparison

To evaluate the effects of the different alignment and pseudo-alignment algorithms on clustering analysis, we created an artificial “ground truth”, where we assigned each barcode to a cell type. For this task we choose SCINA v1.2 [32] as an external classification tool. The semi-supervised classification method in SCINA requires a set of known marker genes for each cell type to be classified. Marker gene sets were obtained from Skelly et. al. [33] and combined with other marker gene sets, as

suggested by Tombor et.al. [34] (Suppl. Table 4). An expectation–maximization (EM) algorithm uses the marker genes to obtain a probability for each provided cell type. After the classification each cell will be assigned a cell type that shows the highest probability based on the provided marker genes. Alignments with different mappers might result in different cell classifications for each barcode. Therefore, a consensus scheme is applied to each sample to create a cell type agreement for each barcode. Consensus of a cell classification for each barcode is achieved if two or more mappers agree on a cell type.

The remaining barcodes were used as a global barcode set for SCINA. Sankey plots were generated with the R-package ggalluvial 0.12.3 [35] to illustrate the representation of cell types in each Seurat cluster (Suppl. Figure 5). In addition, to convey the differences between SCINA and the seurat clusters from each mapper, metrics were calculated. We show the precision, recall and F1-score in Suppl Figure 6. The F1-score of the Cardiac dataset is in Figure 4A.

DEG analysis

For the differential gene expression (DEG) analysis each cluster from the integration in Seurat was assigned to a cell type by known marker genes for the PBMC dataset. The marker genes were obtained by the Seurat workflow for a similar 10X dataset [36]. DEGs were then calculated by using the *FindAllMarkers* function with the Wilcoxon-Rank-Sum test in Seurat and all DEGs above an adjusted p-value of 0.05 were removed. Upset plots were then created with the remaining DEGs (Figure 4).

Additional Software

The R-package ComplexHeatmap 2.6.2 (ComplexHeatmap, RRID:SCR_017270) [37] was used to create the Upset-plots (Figures 2, 4; Suppl. Figure 2).

Hardware

All computations were executed on a workstation with Intel Xeon E5-2667 CPU and 128 GB RAM. The OS was Ubuntu 18.04 LTS.

Results

For the comparison of the five different alignment tools Cell Ranger 6, STARsolo, Alevin, Alevin-fry and Kallisto, we analysed four representative datasets which are denoted as *PBMCs*, *Endothelial, Cardiac (Endothelial)* and *HF* (see method section for a detailed description of the datasets) in the following.

General statistics

The overall performance and basic parameters like runtime, genes per cell, cell number and mapping rate are summarized in Figure 1. In terms of runtime STARsolo, Alevin and Kallisto clearly outperformed Cell Ranger 6 and were at least three times faster. Kallisto showed the shortest runtimes and was on average 4 to 6 times faster than Cell Ranger 6. Additionally, Kallisto and Alevin-fry showed the highest transcriptome mapping rate whereas Alevin showed a slightly decreased mapping rate across all datasets. The cell count and the average genes per cell were similar for Cell Ranger 6 and STARsolo across all datasets. Overall Cell Ranger and STARsolo had almost identical results regarding the cell count and the

genes per cell which is expected from the similarity of both tools. In contrast, Alevin and Kallisto showed different behavior for the genes per cell across the datasets. Compared to the other tools, Alevin detected more cells with fewer genes per cell in the PBMC and Endothelial dataset. However, it detected less cells with more genes per cell in the Cardiac Endothelial and HF dataset. This is caused by the initial whitelisting in Alevin. It calculates a knee point in which all barcodes above the knee point are considered as a putative whitelist. Barcodes below the knee point are then considered as erroneous barcodes. In order to correct these barcodes the algorithm tries to find a barcode in the putative whitelist by a substitution, insertion or deletion. If this approach fails the barcode is considered a noisy barcode and will be removed.

The percentage of noisy barcodes for Alevin is especially high for the HF and the Cardiac dataset. One possible explanation for this could be the library preparation protocol, as these datasets are single nuclei RNA-SEQ (snRNA-SEQ). The single nuclei isolation protocol requires to break the extracellular matrix in order to release the nuclei. This leads to a higher amount of debris which results in a higher percentage of background RNA contamination [38]. The percentage of barcodes which were discarded as *“noisy barcodes”* by Alevin are summarized for each sample in Suppl. Table 5.

We think that the knee point is higher than expected in the Cardiac and HF datasets and the correction fails on many barcodes and, therefore, are removed prior to the mapping. More details with respect to these differences can be found in Suppl. Figure 1. In the PBMC and the Endothelial datasets, Alevin shows small peaks in the lower left corner of the density plots for UMI counts and genes per cell. These peaks represent cells, which have low UMI counts. For the Cardiac dataset Alevin did not

detect these cells with low UMI content, which might explain the lower cell count for this dataset. However, in the Cardiac dataset, we observed more low content cells for Kallisto. This is consistent with the finding that Kallisto detects most cells in the Cardiac dataset.

Cell and gene identification

In 10X droplet based single cell sequencing, the individual cells are usually identified via the randomized cell barcodes, which are predefined by the whitelist. In order to determine if the different mapping tools detected identical cells, we merged the resulting cells based on their barcodes (Figure 2A). The majority of barcodes were identified by all alignment tools. However, Cell Ranger 6, STARsolo and Kallisto detected more barcodes as compared to Alevin and Alevin-fry in the Cardiac and HF dataset. These cells had far less reads per cell compared to the cells that were detected in all mappers, as shown in the panel 1 and 2 of Suppl. Figure 2 A&B. Alevin-fry and Kallisto also detected a set of barcodes. Their gene content is lower than the total dataset as can be seen in panel 3 of Suppl. Figure 2 A&B. Similarly, Alevin detected unique barcodes for the PBMC and Endothelial datasets, which also had less gene content compared to the other cells detected by Alevin (panel 4 of Suppl. Figure 2 A&B). Additionally, we recognised that the majority of these barcodes are not included in the whitelist from 10X (Suppl. Table 6). Panel 5 of Suppl. Figure 2 B shows the unique barcodes for Kallisto in the HF dataset, which also have less gene content than the other cells. Overall, we saw a reduced number of genes per cell for the barcodes that were only detected by one or two of the five alignment tools.

By comparing the expressed genes, we could show that all alignment tools detect a similar set of genes (Figure 2B). Only Kallisto detected additional genes leading to a higher number of protein coding and lncRNA genes compared to the other tools (Suppl. Fig. 3). In the HF dataset a small number of genes were not detected by Alevin-fry and Alevin.

One gene family that occurred more frequently in Kallisto is the Olfr (Olfactory receptor) gene family, which exhibits dramatically enriched UMI counts (Figure 3A). Another Kallisto-enriched gene family is the Vmn (Vomeronasal receptors) family, which is detected with lower UMI counts compared to the Olfr family, but is still elevated compared to the other tools (Figure 3B). This leads to an increase in total gene counts for Kallisto (red line in Figure 3) and an increase of the respective biotypes (Suppl. Figure 3). The increased expression of genes from the Olfr gene family is exemplified in Suppl. Figure 3. The HF dataset shows an increased UMI count of Vmn genes in only 2 or 3 samples. Vomeronasal genes are non-functional in humans because they were deactivated by mutations and therefore should not be expressed in human tissue [39].

Effects on downstream analysis

In order to evaluate downstream effects of the different alignment tools, we performed a semi-supervised cell type assignment with SCINA. Therefore, we used all cells that were found by more than two mappers and assigned them to a corresponding cell type based on the marker genes documented in Suppl. Table 2. Thereby, the majority of barcodes could be assigned to a specific cell type. Then we compared the clusters from each alignment tool to the assigned cell types from

SCINA. Using the barcodes to identify each cell, we traced the cells from their respective clusters to the assigned cell type.

The fate from the predicted cell types to the clusters for each mapper can be observed in the sankey plots in Suppl. Figure 5. Suppl Figure 6 provides metrics in order to further evaluate the detection of barcodes in each tool and cell type. Here, we used a greedy assignment of Seurat clusters with the cell type classification from SCINA. The cluster will be assigned with its highest abundance cell type. Then, precision, recall and F1-scores were calculated.

In general, the clustering was similar when comparing the alignment tools. Minor differences were observed for Kallisto and Alevin. In the PBMC dataset, Kallisto showed a higher number of missing barcodes (M.b.), predominantly from monocytes. Missing barcodes are barcodes that were found in at least two of the other mappers, but not in the present one. Which means that these monocytes were not present or filtered out in Kallisto. This results in a lower recall in Suppl. Figure 6B.

In the Cardiac data set, the lower cell count found by Alevin leads to more barcodes associated with missing barcodes demonstrating that these cells are not detected in Alevin. The majority of these missing cells were assigned as endothelial cells. Which means that in the Cardiac dataset Alevin detected only around 50% of the endothelial cells that were found with the other tools. Also the number of B-cells and granulocytes were decreased due to the lower cell counts. This decrease is reflected in a lower recall in Suppl. Figure 6D and a lower F1-score in Figure 4A. However, the decrease in the latter cell types could not be confirmed in the PBMC dataset.

In summary, Cell Ranger 6 and STARsolo showed the highest agreement with the predicted cell types from SCINA, which is not surprising as they use the same

internal algorithm. The overlaps of Alevin and Kallisto were lower due to varying cell counts.

Analysis of the differential expressed genes for the cell types of the PBMC dataset did show the highest agreement of STARsolo, Alevin-fry and Cell Ranger. Major differences among the alignment tools are summarized in Figure 4.

The accuracy of the barcode detection per tool in each cell type can be seen Figure 4A. The highest accuracy can be seen in Cell Ranger, STARsolo and Alevin. Lower accuracies are present in Alevin and Alevin-fry. Overall, cell types with a low amount of cells present in the dataset are difficult to detect in all tools. Comparing significant DEGs ($p<0.05$) in PBMC, we see in Figure 4A and B that STARsolo or Alevin has the highest overlap and correlation with Cell Ranger, respectively. Overall, Kallisto shows the lowest overlap and Alevin has intermediate overlaps. For the correlation (Figure 4C) this ranking is not as clear as it highly depends on the cell type. Despite the differences most of DEGs were detected by all tools in the PBMC dataset (Figure 4D). Small groups of DEGs were detected by a single tool or when one or two tools have not detected DEGs. This is often the case in Alevin, Alevin-fry and Kallisto. In Figure 4E-H we compare significant DEGs ($p<0.05$) from the T-cells CD4+ cell type of Cell Ranger against the other tools, similar to Kaminov et.al. [20]. The highest correlation can be observed in STARsolo and Alevin-fry. Kallisto shows the lowest correlation against Cell Ranger and Alevin and intermediate correlation. These results are largely consistent with the results from Kaminow et.al. [20]. The uniquely overrepresented genes in Kallisto are likely the OLFR and VMN genes we showed in Figure 3.

Comparing filtered to unfiltered annotations

The default transcriptome annotation dataset, which is recommended for Cell Ranger 6 by 10X Genomics, misses some important biotypes like pseudogenes and TEC’s, sequences that indicate protein coding genes that need to be experimentally confirmed. These differences in gene model compositions can have profound effects on the read mapping and the gene quantification as reported by Zhao et al. [13]. In order to evaluate the effects of different annotation sets on 10x scRNA-seq data, we compared the mapping statistics of the filtered annotations to the complete (unfiltered) Ensembl annotation.

Besides the increase of processed pseudogenes (Suppl. Fig. 3), the usage of the unfiltered annotation led to a decrease in mitochondrial (MT) content across all alignment tools as shown in Suppl. Fig 7A. Especially the two mouse datasets showed a strong reduction of MT content in the unfiltered annotation. Suppl. Fig. 7B shows the amount of reads per mitochondrial gene which are not mapped. Further investigation revealed that the unfiltered annotation includes pseudogenes which are identical to MT genes (Suppl. Fig. 7E). A potential explanation for the reduced MT-content with the unfiltered annotation is that the mapping algorithms cannot uniquely assign a read to the MT-gene, as the read can simultaneously map to the MT-gene and the identical pseudogene (Suppl. Fig. 7D&E). Therefore, this read is discarded. As high MT-content is a sign for damaged or broken cells, cells with an MT-content above a certain threshold are usually filtered out. However due to the reduced MT content less cells surpassed the MT content threshold and we could retrieve more cells. These additional cells clustered along with the other cell types, indicating that the cell quality is good and that these additional cells are not broken or damaged

cells as exemplified in Suppl. Fig. 7C. Using the unfiltered annotation yielded up to 10% more cells per sample. However deeper research is required to ensure the quality of these additional cells.

Discussion

Since handling of scRNA-seq data is a moving target, the constant revision of new tools is important to ensure reliable results. Therefore, independent benchmarking and evaluation of uncertainties of analysis tools is of central importance [40].

Our study of real 10X Genomics data sets demonstrated advantages and disadvantages of five popular scRNA-seq mappers for gene quantification in single cells and adds to the growing number of benchmarks. The tools benchmarked in this study are widely used in many labs, thus, our results are relevant for many scientists working with scRNA-seq data. All mappers have been evaluated on in vivo datasets as these data might reveal unexpected differences or characteristics that probably could not have been found with simulated data as is highlighted by Srivastava et al [41]. From our perspective, the only advantage of simulated datasets is that it allows the assessment of read accuracy, which has already been done for the mappers we used in this study [21,42,43].

The runtime is one of the most important factors when choosing a tool, but the quality of the results is of equal importance. In our detailed analysis, we show that Cell Ranger 6 could be easily replaced with STARsolo, as they show almost identical results but STARsolo is up to 5x faster in comparison with Cell Ranger 6. The low variance in the PBMC dataset for the cell counts and genes per cell for Cell Ranger 6 and STARsolo can be explained by the predefined sample size by 10X. With the

option for selective alignment, which was used throughout this paper, Alevin-fry had a similar runtime than STARsolo. However Kaminov et. al. showed that the runtime decreases when using the pseudo-alignment algorithm (sketch mode) for Alevin-fry, yet this leads to a reduction in accuracy [20] as mapping positions are not validated via alignment scoring [7].

Du et al. 2020 [15] reported that Kallisto was even faster than STARsolo; a finding which is consistent with our results as Kallisto had overall the shortest runtime across all mappers. However, the number of cells and the genes per cell varied across datasets for Alevin and Kallisto.
Additionally, Kallisto seems to detect genes of the Vmn and Olfr family as highly expressed in several single cell data sets, although these genes are typically not expressed in these tissues. As these gene families belong to the group of sense and smell receptors, they are expected to be expressed at lower levels or be absent in PBMCs and heart tissue and likely represent artefacts. We consistently show that these genes are overrepresented in the Kallisto results (Figure 3 and Suppl. Figure 4). As Kallisto does not perform quality filtering for UMIs this might have influenced the reported number of genes per cell as is indicated by Parekh et al [44].

Another major difference of the tested mapping tools is the handling of errors in the barcodes. We could show that Alevin often detects unique barcodes, which were not identified by the other tools. These barcodes had very low UMI content and were not listed in the 10X whitelist. Therefore, It can be assumed that these barcodes were poorly assigned (Suppl. Figure 2, Section 4). A possible explanation might be the usage of a putative whitelist in Alevin that was calculated prior to the mapping,

instead of using the one provided by 10X. In Alevin-fry the barcode correction seems to be improved as there is no severe enrichment of cells that are unique to Alevin-fry.

While comparing the resulting cell clusters generated by each tool, we recognised only minor differences between the tools. Especially the clusters from Cell Rranger and STARsolo were similar. However, Kallisto detected fewer monocytes in the PBMC dataset and Alevin detected fewer endothelial cells in the cardiac dataset. Overall, we saw a much higher variance in the clustering in the cardiac dataset. This could be due to the use of an older version of the library extraction protocol (10X v2), which has short barcode and UMI sequences, or a lower sequencing quality of the Cardiac dataset.

The comparison of the complete annotation from Ensembl and the filtered annotation, as suggested by 10X, revealed that multi-mapped reads play an important role in scRNA-seq analysis. In this study, we showed that using an unfiltered annotation reduces the MT-content of cells compared to the filtered annotation. Therefore, the mitochondrial content as a way to distinguish valid cells and dead or damaged cells has to be carefully conducted as it depends on the annotation. The recommended annotation from 10X, which only contains genes with the biotypes protein coding gene and long non-coding gene, might lead to an overestimation of mitochondrial gene expression. However, on the other side all of these genomic loci that are identical to MT genes, so called nuclear mitochondrial DNA (NUMT), are unprocessed pseudogenes and are not yet experimentally validated and could well be artifacts from the genome assembly. For human samples we could not see major differences in the downstream results while using the complete annotation, therefore it might well be used instead of the filtered

annotation. However for mouse samples a clear recommendation of whether to use the filtered or the complete annotation cannot be made, as more research into this issue is required. These results suggest that there is still a need to improve the handling of multi-mapped reads in scRNA-seq data. In datasets with a high percentage of multi-mapped reads EM-like algorithms, as suggested by Srivastava et.al [45] can be advantageous and improve gene quantification in scRNA-SEQ datasets. Future mapping tools might for example consider the likelihood of a gene to be expressed in a certain cell type. This might enhance the quantification of cell type-specific genes and prevent multi-mapped reads for cell types, where a certain gene is rarely expressed. Inclusion of mapping uncertainties may be another fruitful direction.

Srivastava et al. [41] observed that there are significant differences between methods that align against the transcriptome with quasi-mapping (e.g. Alevin) and methods that do full spliced alignments against the genome (e.g. STAR) [41]. The observed discrepancies, when using the filtered annotation in our experiments, often result from genes that share the same sequences, and therefore, the true alignment origin cannot be determined. The reported positions of reads contained annotated transcripts e.g. from the mitochondria and a few unprocessed pseudogenes.

In conclusion, our analysis shows that Alevin, Kallisto and STARsolo are very fast and reliable alternatives to Cell Ranger 6. They also scale to large datasets. A summary of advantages and disadvantages of each individual tool is provided in Figure 5.

In general, we could show that STARsolo is an ideal substitute for Cell Ranger 6, as it is faster but otherwise performs similarly. If high-quality cell counts need to be obtained, Alevin-fry appears to be the most suitable method, as average gene

counts are high and poor-quality barcodes are seldom reported. Kallisto, while reporting the highest number of barcodes, also contains many barcodes that could not be assigned to cells expected in the heart based on known marker genes. Therefore, we generally recommend STARsolo or Alevin-fry for most end-users as an alternative to Cell Ranger as these tools perform very stable over all datasets. For very large projects with a high number of samples, pseudo-alignment tools such as Kallisto can be advantageous in terms of runtime and storage efficiency, at the cost of a slight reduction in accuracy.

Availability of Source Code and Requirements

- Project name: Comparative Analysis of common alignment tools for single cell RNA sequencing
- Project home page: https://github.com/rahmsen/BenchmarkAlignment
- Operating system(s): x86_64-pc-linux-gnu (64-bit)
- Programming language: R (version 3.6.2)
- Other requirements: Cell Ranger 6.0, STARsolo 2.7.4a, Salmon 1.5.1, Alevin 1.1.0, Alevin-fry 0.4.0, Kallisto 0.46.1, Seurat 4.0.3, DropletUtils 1.6.1, SCINA v1.2, ggalluvial 0.12.3, ComplexHeatmap 2.6.2, reshape2 1.4.4, ggplot 3.3.5, ggpubr 0.4.0, dplyr 1.0.7, svglite 2.0.0, jsonlite 1.7.2, egg 0.4.5
- License: MIT

Data Availability

All supporting data and materials are available in the *GigaScience* GigaDB database [46].

Abbreviations

scRNA-seq: single cell RNA sequencing; NGS: next generation sequencing; UMI: unique molecular identifier; PCR: Polymerase chain reaction; PBMC: Peripheral blood mononuclear cell; lncRNA: long non-coding RNA; MM: mismatch; GTF: General Feature Format; DEG: Differentially expressed genes; UMAP: Uniform Manifold Approximation and Projection; SCINA: Semi-Supervised Subtyping Algorithm; Vmn: Vomeronasal receptor; Olfr: Olfactory receptor; PCA: Principal component analysis; M.b.: Missing barcodes; MT: mitochondrial; NUMT: nuclear mitochondrial DNA

Competing Interests

The authors declare that they have no competing interests

Funding

This study is supported by the Dr. Robert Schwiete Foundation, the Cardio-Pulmonary Institute Frankfurt and the German Center for Cardiovascular Research (DZHK).

Authors' contributions

M.H.S., SD and DJ. designed research and conceptualization; R.S.B., L.S.T, M.H.S, D.J. performed research and data curation; R.S.B., L.S.T and D.J. analyzed data and implemented software; R.S.G., M.H.S., S.D. and D.J. wrote the paper; R.S.B., L.S.T. and D.J. accomplished visualization; M.H.S., S.D. and D.J. performed supervision of the project.

Acknowledgements

We thank Alexander Dobin, the Patro-Lab, 10X as wel as Patcher-Lab for supportive information and detailed answers for all our questions.

Figure Descriptions

Figure 1: Summary of major measurements including runtime in hours (A), Genes per cell (B), cell count (C) and the mapping rate in percent (D). All bar plots show the mean of all samples with the standard error.

Figure 2: The chart shows the barcodes (A) or genes (B) that have been detected by a certain number of mappers according to datasets. The number of mappers increases from right to left. First the barcodes or genes that have only been detected by one mapper up to the barcodes or genes that have been detected in all tools.

Figure 3: UMI counts of all detected (A) Vmn (Vomeronasal receptor genes) and (B) Olfr (Olfactory receptor genes) genes per mapper in each sample. The red line indicates the total number of expressed genes in the gene families.

Figure 4: Accuracy of cell annotation in Seurat compared with the barcode consensus scheme from SCINA (A). Differential gene expression (DEGs) between Cell Ranger and the other tools as overlap (B) and correlation (C). Intersection that shows the detection of DEGs by a varying number of tools. The number of tools increases from right (DEGs that were detected by one tool) to left (DEGs that were detected by all tools) (D). Log2FC of DEGs CD4+ T-cells between Cell Ranger and each of the other tools (E-H). The adjusted R^2 is the sample correlation of a linear model.

Figure 5: Summary of the results for each evaluated section of interest and mapper. Good results are colored in green, intermediate in yellow and poor results in red.

Suppl. Figure 1 Distribution of UMI-counts and genes per cell for the individual data sets. Distribution is a kernel density estimate with a gaussian kernel of all samples for the PBMC, Endothelial and Cardiac data set. The left column displays the UMI counts per cell and on the right column the number of genes per cell.

Suppl. Figure 2 (A) Amount of common and unique barcodes (mean± s.e.m.) detected by the individual alignment tools. Intersections of interest are marked by numbers. (B) Gaussian distribution of genes per cells the interesting intersection and dataset from A. The distributions of the tools from the intersection (non-transparent) are compared with all detected barcodes of each tool (transparent lines (in the background); denoted with '*' in the legend)

Suppl. Figure 3 Number (mean+s.e.m) of biotypes per dataset with at least 1 UMI count after mapping with a filtered (solid dots) or unfiltered annotation (square-triangles). IG = Immunoglobulin genes, TR = T-cell receptor genes, TEC = Sequences that need To be Experimentally Confirmed.

Suppl. Figure 4 Expression of the OLFR gene family per cell in the PBMC data set for (A) Cell Ranger, (B) Cell Ranger 6, (C) STARsolo, (D) Alevin and (E) Kallisto. Cells are sorted by clusters that are denoted by the color code above each heatmap.

Suppl. Figure 5 Sankey plots demonstrating the fate of each cell from SCINA cell types to the clusters obtained by Seurat. Only cells were kept if more than two mappers detected a barcode. (A) represent the PBMC data set and (B) the Cardiac data set. M.b. stands for

missing barcodes. These are barcodes that were found in at least two of the other mappers, but not in the present one.

Suppl. Fig. 6 Consistency of cells detected by each mapper (“ground truth”) by greedy assignment of the barcodes to the SCINA classification. (A) F1-Score, (B) Recall and (C) precision for the PBMC dataset. The recall (D)and precision (E) for the Cardiac dataset.

Suppl. Figure 7 Difference in mitochondrial content (mt-content) of cells due to usage of a filtered and unfiltered annotation. A) MT-content of cells separated by filtered and unfiltered annotation. B) Reads mapped to the mitochondrial genes for the PBMC and Rosenthal data set with unfiltered annotation. Orange indicating the amount of reads that are removed due to multimapping when an unfiltered annotation is used. C) UMAP showing cells in green that are retained because the MT-content is below the filtering threshold when the unfiltered annotation was used in the mapping. D) Mitochondrial genes and its closest pseudogene when the mappers reported the secondary mapping position along with the sequence similarity to the MT gene. E) Example of the mapping process of a read from a MT gene with a filtered/unfiltered annotation. As the filtered annotation does not include potential NUMT’s, the read is uniquely mapped to the MT gene. Whereas the complete set contains NUMT’s and therefore the read cannot be uniquely mapped to the MT genes (multi-mapped) and therefore is discarded from counting.

References

1. Wagner A, Regev A, Yosef N. Revealing the vectors of cellular identity with single-cell genomics. *Nat Biotechnol*. doi: 10.1038/nbt.3711.

2. Abplanalp WT, John D, Cremer S, Assmus B, Dorsheimer L, Hoffmann J, et al.. Single-cell RNA-sequencing reveals profound changes in circulating immune cells in patients with heart failure. *Cardiovasc Res*. 2021; doi: 10.1093/cvr/cvaa101.

3. Vidal R, Wagner JUG, Braeuning C, Fischer C, Patrick R, Tombor L, et al.. Transcriptional heterogeneity of fibroblasts is a hallmark of the aging heart. *JCI Insight*. 2019; doi:

10.1172/jci.insight.131092.

4. Zheng GXY, Terry JM, Belgrader P, Ryvkin P, Bent ZW, Wilson R, et al.. Massively parallel digital transcriptional profiling of single cells. *Nat Commun*. 2017. doi: 10.1038/ncomms14049.

5. Dobin A, Davis CA, Schlesinger F, Drenkow J, Zaleski C, Jha S, et al.. STAR: ultrafast universal RNA-seq aligner. *Bioinformatics*. 2013; doi: 10.1093/bioinformatics/bts635.

6. Melsted P, Booeshaghi AS, Liu L, Gao F, Lu L, Min KHJ, et al.. Modular, efficient and constant-memory single-cell RNA-seq preprocessing. *Nat Biotechnol*. 2021; doi: 10.1038/s41587-021-00870-2.

7. He D, Zakeri M, Sarkar H, Soneson C, Srivastava A. Alevin-fry unlocks rapid, accurate, and memory-frugal quantification of single-cell RNA-seq data. *bioRxiv*. biorxiv.org; 2021; https://doi.org/10.1101/2021.06.29.450377

8. Srivastava A, Malik L, Smith T, Sudbery I, Patro R. Alevin efficiently estimates accurate gene abundances from dscRNA-seq data. *Genome Biol*. 2019; doi: 10.1186/s13059-019-1670-y.

9. Patro R, Mount SM, Kingsford C. Sailfish enables alignment-free isoform quantification from RNA-seq reads using lightweight algorithms. *Nat Biotechnol*. 32:4622014;

10. Patro R, Duggal G, Love MI, Irizarry RA, Kingsford C. Salmon provides fast and bias-aware quantification of transcript expression. *Nat Methods*. Nature Publishing Group; 2017; doi: 10.1038/nmeth.4197.

11. Wu DC, Yao J, Ho KS, Lambowitz AM, Wilke CO. Limitations of alignment-free tools in total RNA-seq quantification. *BMC Genomics*. doi: 10.1186/s12864-018-4869-5.

12. 10x Genomics: Gene Expression Algorithm Overview. https://support.10xgenomics.com/single-cell-gene-expression/software/pipelines/3.1/algorithms/overview

13. Zhao S, Zhang B. A comprehensive evaluation of ensembl, RefSeq, and UCSC annotations in the context of RNA-seq read mapping and gene quantification. *BMC Genomics*. 2015; doi: 10.1186/s12864-015-1308-8.

14. Lähnemann D, Köster J, Szczurek E, McCarthy DJ, Hicks SC, Robinson MD, et al.. Eleven grand challenges in single-cell data science. *Genome Biol*. 2020; doi: 10.1186/s13059-020-1926-6.

15. Du Y, Huang Q, Arisdakessian C, Garmire LX. Evaluation of STAR and Kallisto on Single Cell RNA-Seq Data Alignment. G3 Genes|Genomes|Genetics. 2020. doi: 10.1534/g3.120.401160.

16. Chen W, Zhao Y, Chen X, Yang Z, Xu X, Bi Y, et al.. A multicenter study benchmarking single-cell RNA sequencing technologies using reference samples. *Nat Biotechnol*. 2020; doi: 10.1038/s41587-020-00748-9.

17. Vieth B, Parekh S, Ziegenhain C, Enard W, Hellmann I. A systematic evaluation of single cell RNA-seq analysis pipelines. *Nat Commun*. 2019; doi: 10.1038/s41467-019-12266-7.

18. Booeshaghi AS, Pachter L. Benchmarking of lightweight-mapping based single-cell RNA-seq pre-processing. *bioRxiv*. 2021; doi: 10.1101/2021.01.25.428188.

19. Zakeri M, Srivastava A, Sarkar H, Patro R. A like-for-like comparison of lightweight-mapping pipelines for single-cell RNA-seq data pre-processing. *bioRxiv*. biorxiv.org; 2021; https://doi.org/10.1101/2021.02.10.430656

20. Kaminow B, Yunusov D, Dobin A. STARsolo: accurate, fast and versatile mapping/quantification of single-cell and single-nucleus RNA-seq data. *bioRxiv*. biorxiv.org; 2021; https://doi.org/10.1101/2021.05.05.442755

21. Mangul S, Martin LS, Hill BL, Lam AK-M, Distler MG, Zelikovsky A, et al.. Systematic benchmarking of omics computational tools. *Nat Commun*. 2019; doi: 10.1038/s41467-019-09406-4.

22. 10x Genomics: 5k Peripheral blood mononuclear cells (PBMCs) from a healthy donor (v3 chemistry). https://support.10xgenomics.com/single-cell-gene-expression/datasets/3.0.2/5k_pbmc_v3 (2019).

23. Forte E, Skelly DA, Chen M, Daigle S, Morelli KA, Hon O, et al.. Dynamic Interstitial Cell Response during Myocardial Infarction Predicts Resilience to Rupture in Genetically Diverse Mice. *Cell Rep*. doi: 10.1016/j.celrep.2020.02.008.

24. Kalucka J, de Rooij LPMH, Goveia J, Rohlenova K, Dumas SJ, Meta E, et al.. Single-Cell Transcriptome Atlas of Murine Endothelial Cells (complete with methods). *Cell*. doi: 10.1016/j.cell.2020.01.015.

25. Yates AD, Achuthan P, Akanni W, Allen J, Allen J, Alvarez-Jarreta J, et al.. Ensembl 2020. *Nucleic Acids Res*. 2020; doi: 10.1093/nar/gkz966.

26. Frankish A, Diekhans M, Ferreira A-M, Johnson R, Jungreis I, Loveland J, et al.. GENCODE reference annotation for the human and mouse genomes. *Nucleic Acids Res*. 2019; doi: 10.1093/nar/gky955.

27. 10x Genomics: Build Notes for Reference Packages. https://support.10xgenomics.com/single-cell-gene-expression/software/release-notes/build

28. Schulze Brüning R: Comparative Analysis of common alignment tools for single cell RNA sequencing. https://github.com/rahmsen/BenchmarkAlignment (2021).

29. Griffiths JA, Richard AC, Bach K, Lun ATL, Marioni JC. Detection and removal of barcode swapping in single-cell RNA-seq data. *Nat Commun*. 2018; doi: 10.1038/s41467-018-05083-x.

30. Lun ATL, Riesenfeld S, Andrews T, Dao TP, Gomes T, Marioni JC, et al.. EmptyDrops: distinguishing cells from empty droplets in droplet-based single-cell RNA sequencing data. *Genome Biol*. 2019; doi: 10.1186/s13059-019-1662-y.

31. Stuart T, Butler A, Hoffman P, Hafemeister C, Papalexi E, Mauck WM, et al.. Comprehensive Integration of Single-Cell Data. *Cell*. 2019; doi: 10.1016/j.cell.2019.05.031.

32. Zhang Z, Luo D, Zhong X, Choi JH, Ma Y, Wang S, et al.. SCINA: A Semi-Supervised Subtyping Algorithm of Single Cells and Bulk Samples. *Genes* . doi: 10.3390/genes10070531.

33. Skelly DA, Squiers GT, McLellan MA, Bolisetty MT, Robson P, Rosenthal NA, et al.. Single-Cell Transcriptional Profiling Reveals Cellular Diversity and Intercommunication in the Mouse Heart. *Cell Rep*. 2018; doi: 10.1016/j.celrep.2017.12.072.

34. Tombor LS, John D, Glaser SF, Luxán G, Forte E, Furtado M, et al.. Single cell

sequencing reveals endothelial plasticity with transient mesenchymal activation after myocardial infarction. *Nat Commun*. 2021; doi: 10.1038/s41467-021-20905-1.

35. Brunson JC. ggalluvial: Alluvial Plots in “ggplot2”. R package version 0.12.3. *Journal of Open Source Software*. 5:20172020;

36. Seurat: Guided Clustering Tutorial. https://satijalab.org/seurat/v3.2/pbmc3k_tutorial.html (2020).

37. Gu Z, Eils R, Schlesner M. Complex heatmaps reveal patterns and correlations in multidimensional genomic data. *Bioinformatics*. 2016; doi: 10.1093/bioinformatics/btw313.

38. Nguyen QH, Pervolarakis N, Nee K, Kessenbrock K. Experimental Considerations for Single-Cell RNA Sequencing Approaches. *Front Cell Dev Biol*. 2018; doi: 10.3389/fcell.2018.00108.

39. Trotier D. Vomeronasal organ and human pheromones. *Eur Ann Otorhinolaryngol Head Neck Dis*. 2011; doi: 10.1016/j.anorl.2010.11.008.

40. Weber LM, Saelens W, Cannoodt R, Soneson C, Hapfelmeier A, Gardner PP, et al.. Essential guidelines for computational method benchmarking. *Genome Biol*. doi: 10.1186/s13059-019-1738-8.

41. Srivastava A, Malik L, Sarkar H, Zakeri M, Almodaresi F, Soneson C, et al.. Alignment and mapping methodology influence transcript abundance estimation. *Genome Biol*. 2020; doi: 10.1186/s13059-020-02151-8.

42. Zhang C, Zhang B, Lin L-L, Zhao S. Evaluation and comparison of computational tools for RNA-seq isoform quantification. *BMC Genomics*. 2017; doi: 10.1186/s12864-017-4002-1.

43. Teissandier A, Servant N, Barillot E, Bourc’his D. Tools and best practices for retrotransposon analysis using high-throughput sequencing data. *Mob DNA*. 2019; doi: 10.1186/s13100-019-0192-1.

44. Parekh S, Ziegenhain C, Vieth B, Enard W, Hellmann I. zUMIs - A fast and flexible pipeline to process RNA sequencing data with UMIs. *Gigascience*. 2018; doi: 10.1093/gigascience/giy059.

45. Srivastava A, Malik L, Sarkar H, Patro R. A Bayesian framework for inter-cellular information sharing improves dscRNA-seq quantification. *Bioinformatics*. 2020; doi: 10.1093/bioinformatics/btaa450.

46. Brüning RS; Tombor LS; Schulz MH; Dimmeler S; John D. Supporting data for

"Comparative Analysis of common alignment tools for single cell RNA sequencing"

GigaScience Database 2021. http://doi.org/10.5524/100966

Figure_1

Click here to access/download;Figure;Figure_1.pdf

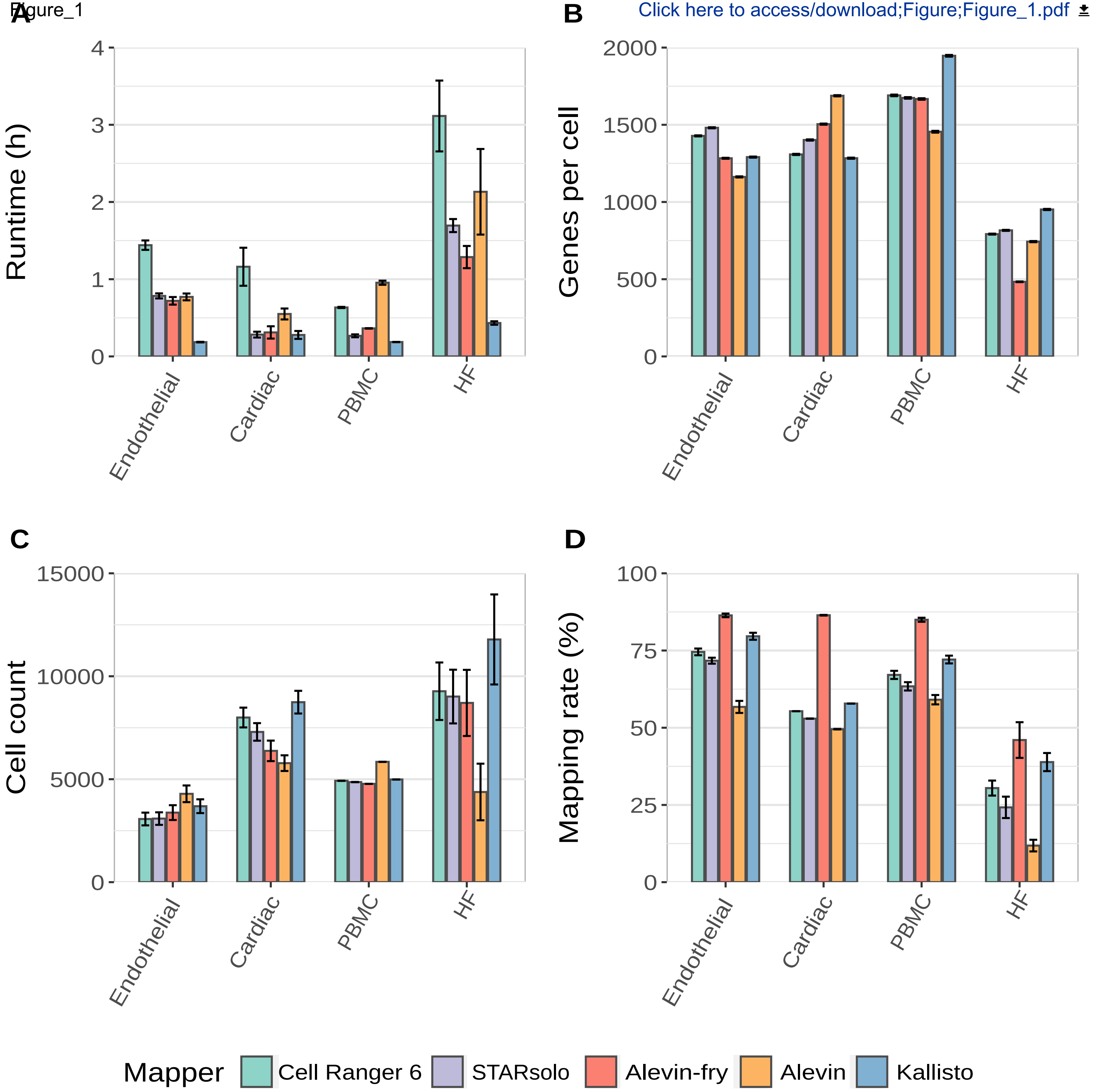

Click here to access/download;Figure;Figure_2.pdf

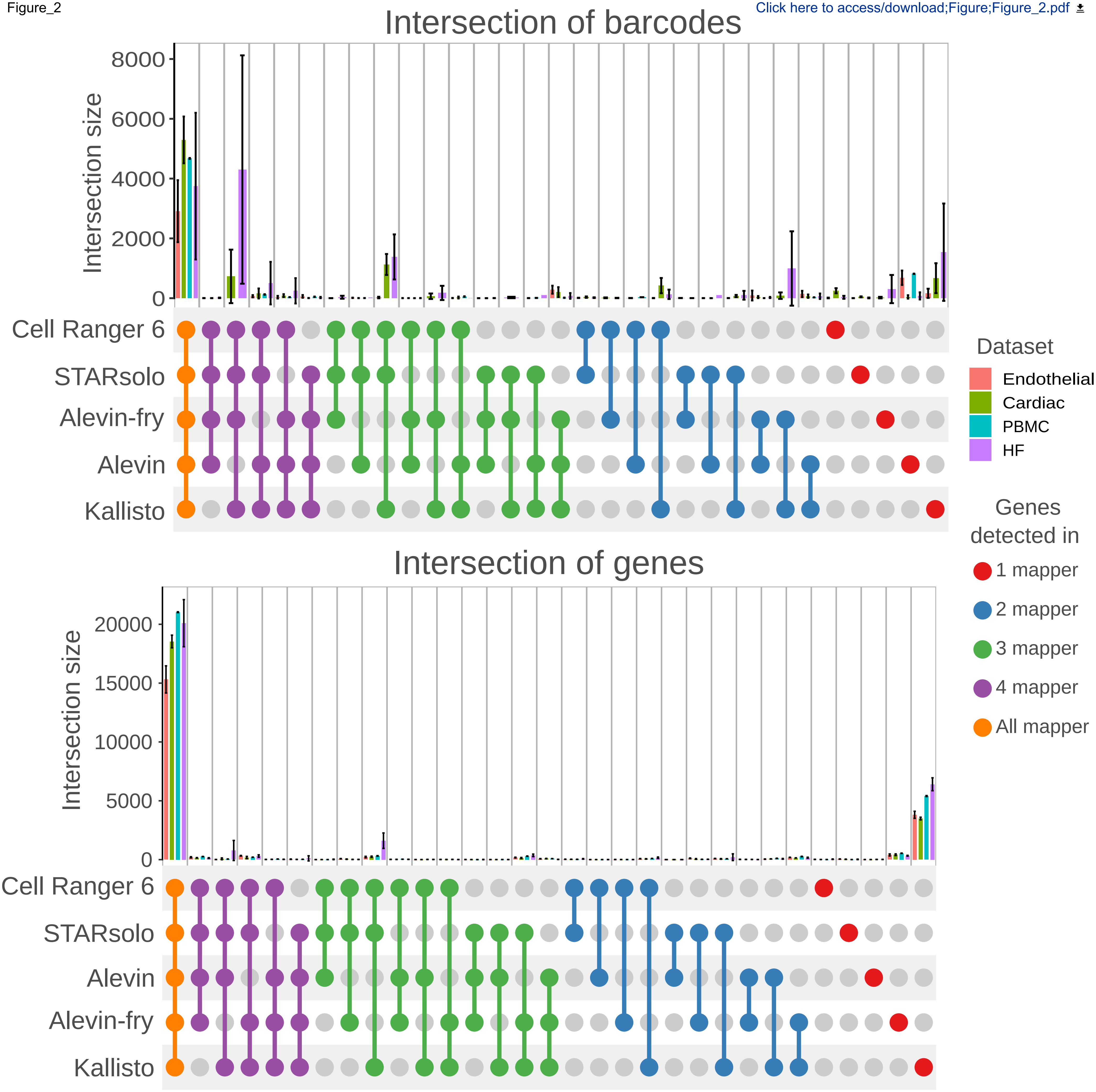

Figure_3

Click here to access/download;Figure;Figure_3.pdf

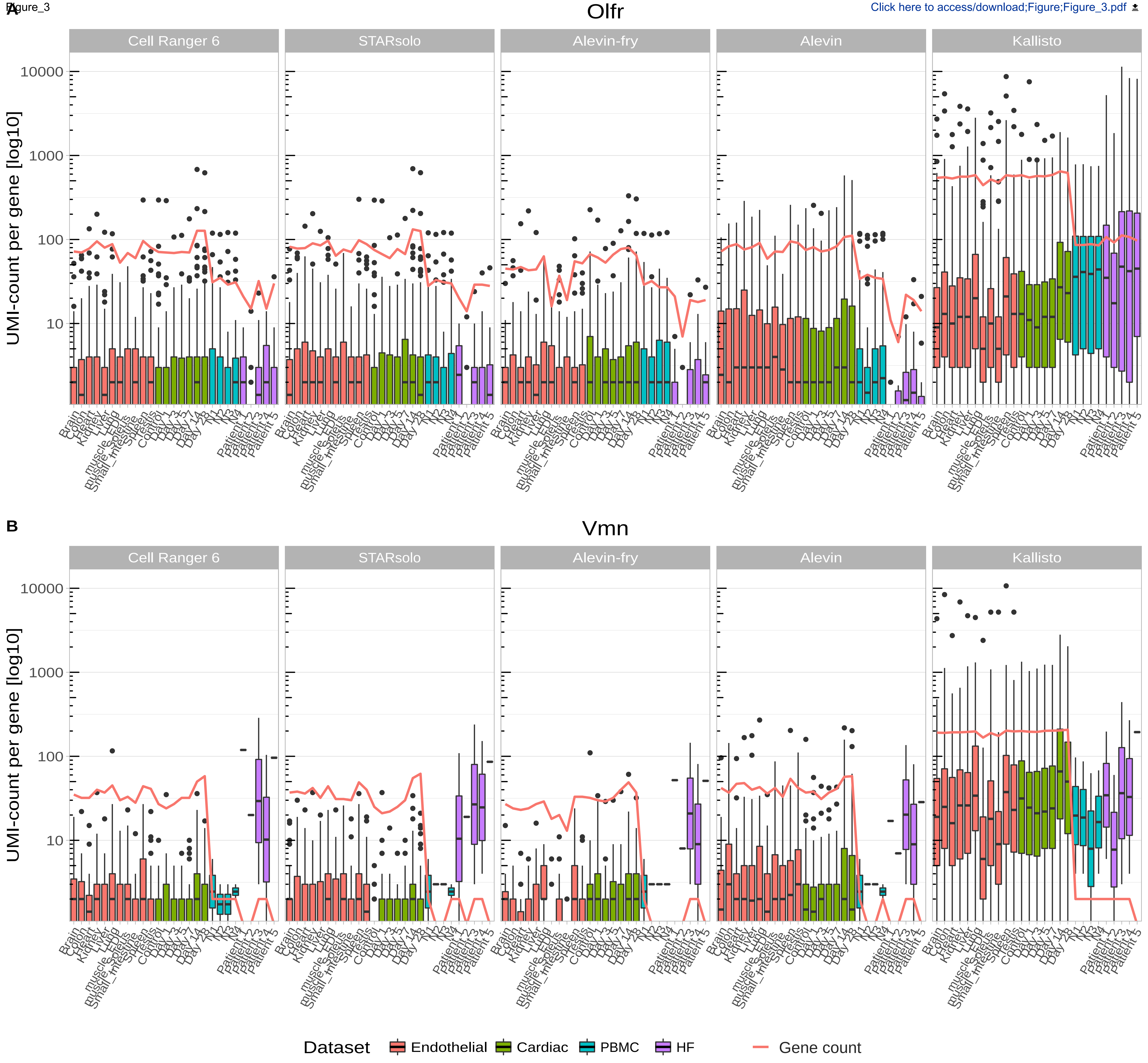

Figure_4

Click here to access/download;Figure;Figure_4.pdf

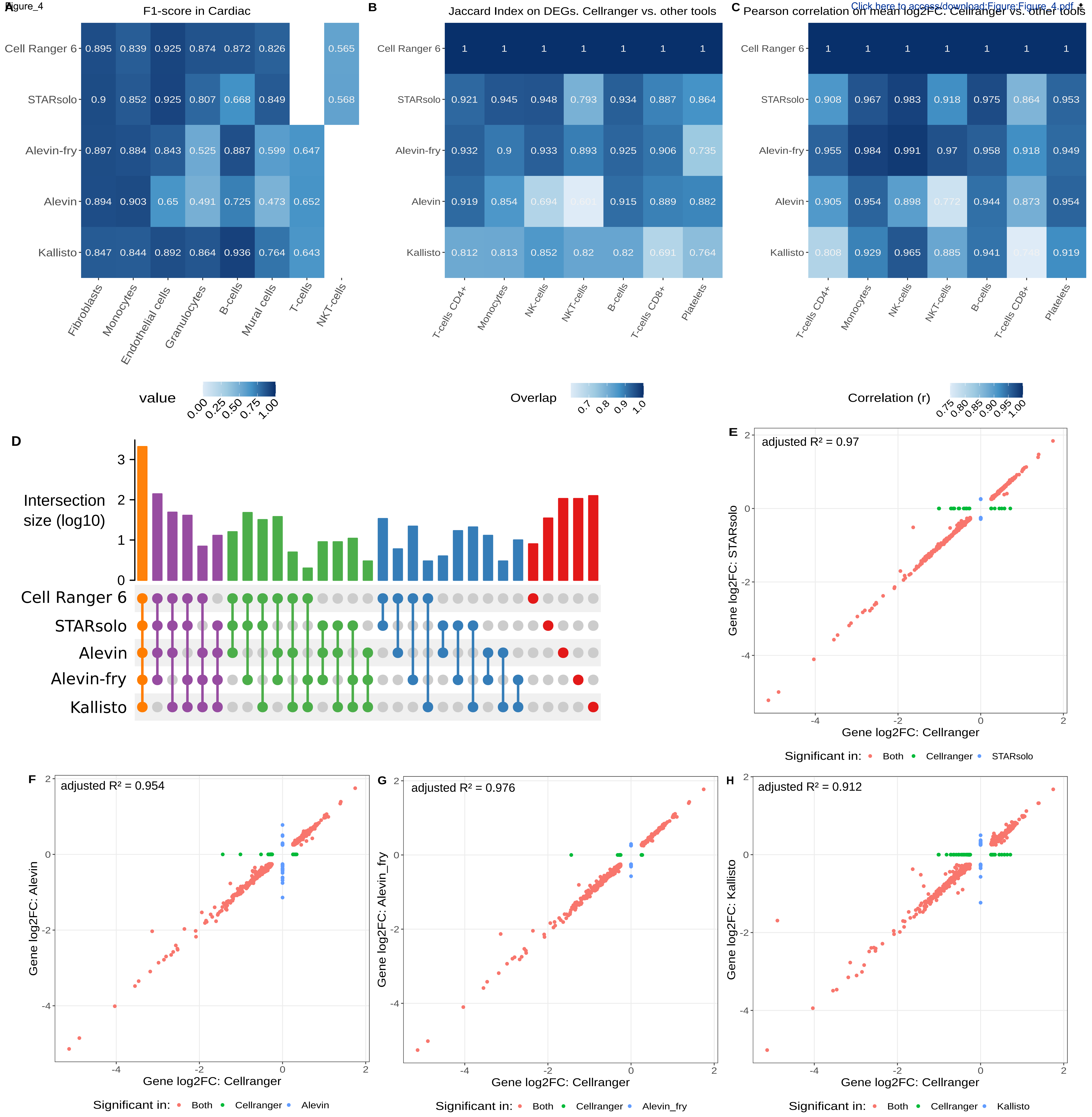

 Click here to access/download;Figure;Figure_5.pdf

Summary

	Cell Ranger	STARsolo	Alevin	Alevin-fry	Kallisto
**Mapping performance**	Longest runtime	- Short runtime<br>- Comparable results with Cell Ranger	- Whitelisting causes loss or gain of barcodes	- Faster mapping in comparison with Alevin.<br>- Pseudoalignment (sketch mode) further decreases runtime	- Shortest runtime<br>- highest mapping rate
**Barcode correction and filtering**			- Detected barcodes that are not in the whitelist	- More barcodes are retained than in Alevin	- Reports more cells
**Gene discovery**				- Lower detection of Vmn and Olfr gene family than in Alevin	- Highest detection rate of genes<br>- Highest UMI count for genes not expressed in studied tissue
**Differences between filtered and unfiltered annotation**	- Multi-mapped reads are discarded	- Multi-mapped reads are discarded<br>- EM-algorithm can be used (optional)	- Counts of mullti-mapped reads split with EM-algorithm	- Multi-mapped reads are discarded<br>- EM-algorithm can be used (optional)	- Multi-mapped reads are discarded<br>- EM-algorithm can be used (optional)
**Clustering**	- Highest Overlap with SCINA classification	- Very similar to Cell Ranger with minor differences	- Cell types contain lower amount of cells with SCINA classification		- High amount of barcodes not detected
**DEG**	- No difference detected	- No difference detected	- Lower detection rate than STARsolo and Alevin-fry	- Improved concordance (than Alevin) with Cell Ranger	- Lowest concordance with Cell Ranger
**Practical Recommendation**	- Replacement with STARsolo is recommended	- Recommended as a general purpose mapper		- Pseudoalignment is especially suitable for huge datasets	- Fast mapper<br>- qualitative issues with gene detection

Click here to access/download
Supplementary Material
Suppl_Figure_1_supplementary_material.pdf

Click here to access/download
Supplementary Material
Suppl_Figure_2_supplementary_material.pdf

Click here to access/download
Supplementary Material
Suppl_figure_3_supplementary_material.pdf

Supplementary_Figure_4_Supplementary Material

Click here to access/download
Supplementary Material
Suppl_figure_4_supplementary_material.png

Click here to access/download
Supplementary Material
Suppl_figure_5_supplementary_material.pdf

Click here to access/download
Supplementary Material
Suppl_figure_6_supplementary_material.pdf

Click here to access/download
Supplementary Material
Suppl_figure_7_supplementary_material.pdf

Supplementary_Table_1_Supplementary Material

Click here to access/download
Supplementary Material
Suppl_Table_1_supplementary_material.pdf

Click here to access/download
Supplementary Material
Suppl_Table_2_supplementary_material.pdf

Click here to access/download
Supplementary Material
Suppl_Table_3_supplementary_material.pdf

Click here to access/download
Supplementary Material
Suppl_table_4_supplementary_material.pdf

Click here to access/download
Supplementary Material
Suppl_table_5_supplementary_material.pdf

Click here to access/download
Supplementary Material
Suppl_Table_6_supplementary_material.pdf
