# Supplementary material for: Comparative analysis of common alignment tools for single-cell RNA sequencing
Source: Gigascience. 2022 Jan 27;11:giac001. doi: 10.1093/gigascience/giac001 (PMC8848315; doi:10.1093/gigascience/giac001)
Supplement: giac001_Supplemental_Files [file giac001_supplemental_files.zip › Suppl_Figure_1_supplementary_material.pdf]

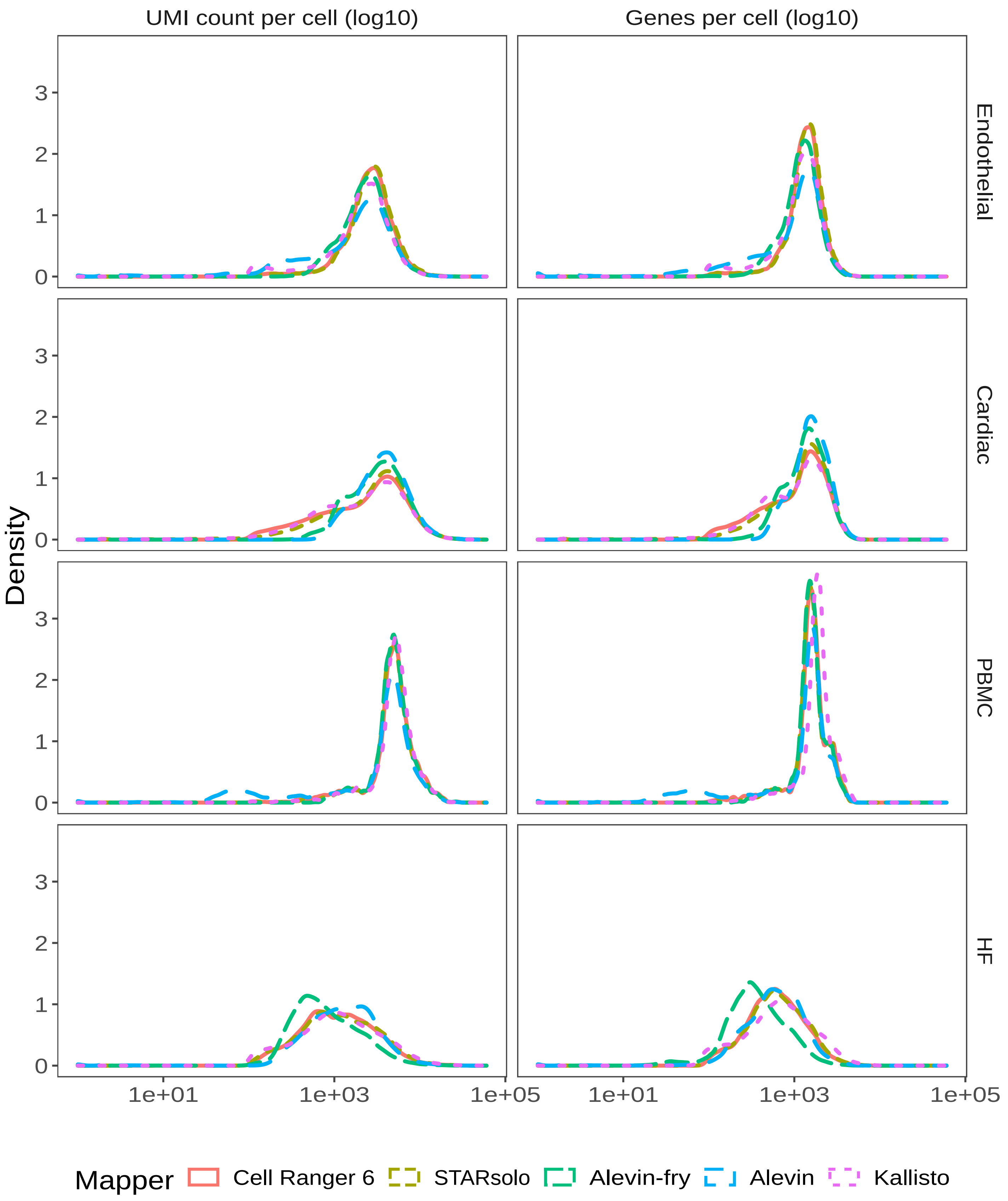
UMI count per cell (log10)
Genes per cell (log10)
Density
Endothelial
Cardiac
PBMC
HF
0
1
2
3
1e+01
1e+03
1e+05
Mapper
Cell Ranger 6
STARsolo
Alevin-fry
Alevin
Kallisto
