# Supplementary material for: Comparative analysis of common alignment tools for single-cell RNA sequencing
Source: Gigascience. 2022 Jan 27;11:giac001. doi: 10.1093/gigascience/giac001 (PMC8848315; doi:10.1093/gigascience/giac001)

A

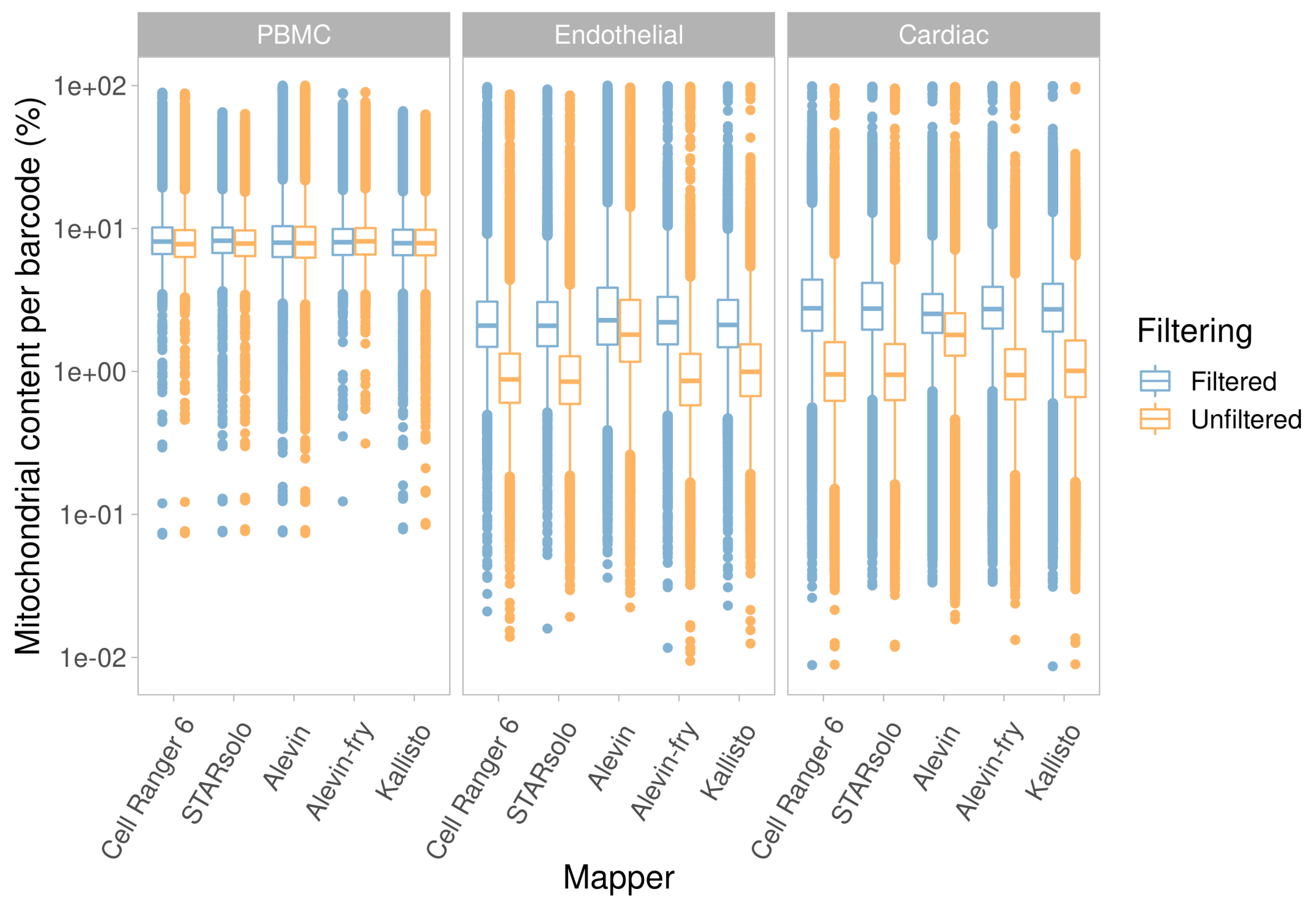

B

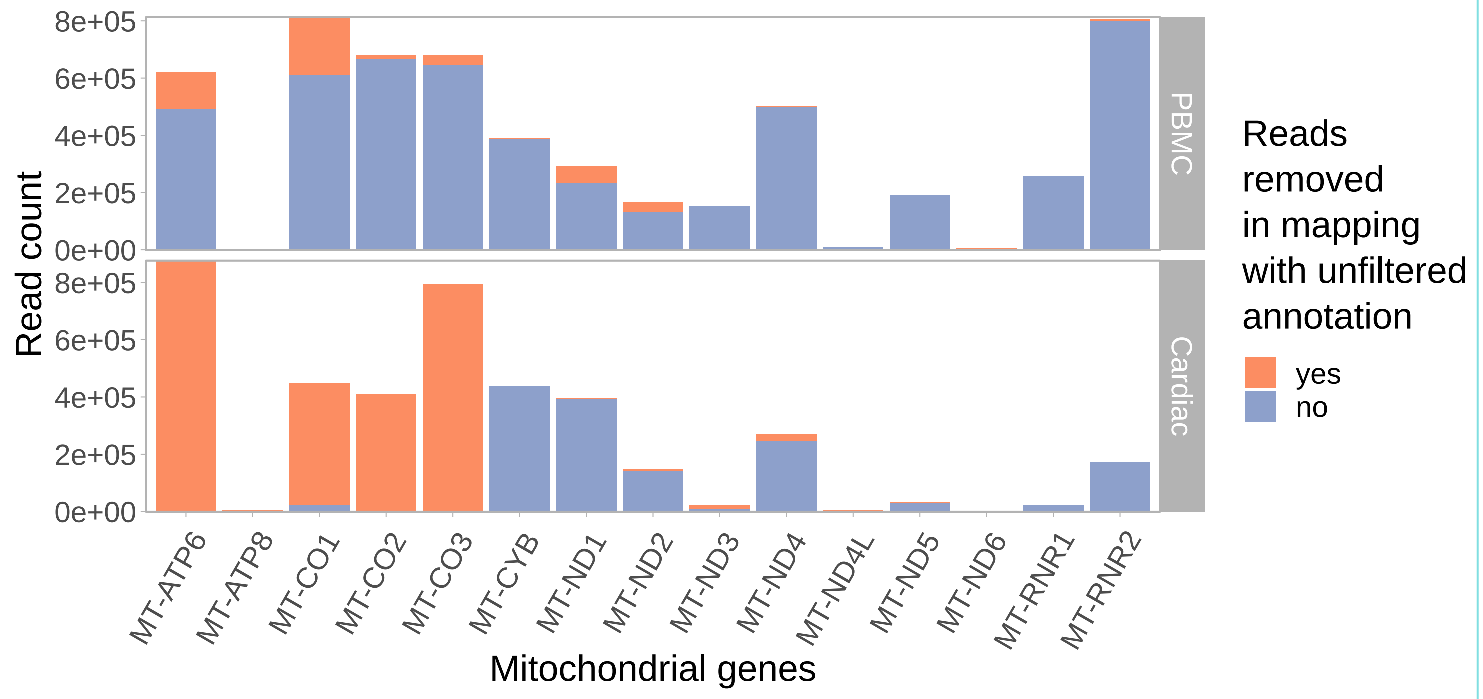

C

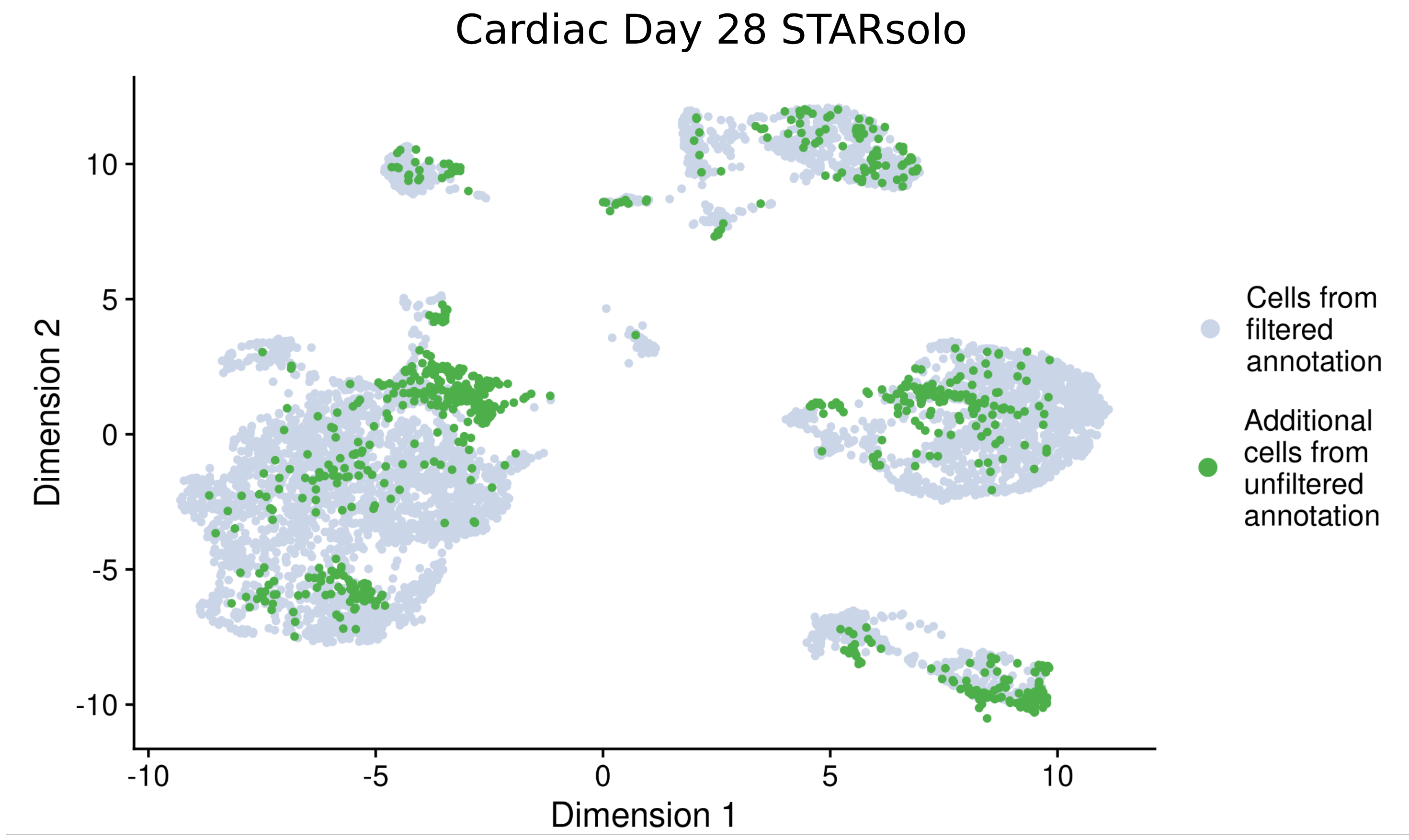

D

Mouse

Gene	Secondary mapping	Secondary mapping gene type	chr	Sequence similarity to MT gene (%)
Mt-Atp6	GM10925	unprocessed pseudogene	1	100
Mt-Co1	Gm29216	unprocessed pseudogene	1	100
Mt-Co2	Gm28661	unprocessed pseudogene	1	100
Mt-Co3	Gm28437	unprocessed pseudogene	1	100
Mt-Cytb	GM11410	processed pseudogene	4	92.91
Mt-Nd2	Gm13339	unprocessed pseudogene	2	96.67
Mt-Nd3	Gm28438	unprocessed pseudogene	1	99.71
Mt-Nd4	Gm28439	unprocessed pseudogene	1	100
Mt-Nd4l	Gm10222	unprocessed pseudogene	1	100
Mt-Nd5	Gm11407	processed pseudogene	4	92.4

Human

Gene	Secondary mapping	Secondary mapping gene type	chr	Sequence similarity to MT gene (%)
MT-ATP6	MTATP6P1	unprocessed pseudogene	1	98.53
MT-CO1	MTCO1P12	unprocessed pseudogene	1	98.64
MT-CO2	MTCO2P12	unprocessed pseudogene	1	97.95
MT-CO3	MTCO3P12	unprocessed pseudogene	1	98.18
MT-ND1	MTND1P23	unprocessed pseudogene	1	99.43
MT-ND2	MTND2P28	unprocessed pseudogene	1	98.37
MT-ND4	MTND4P12	processed pseudogene	5	95.72

E

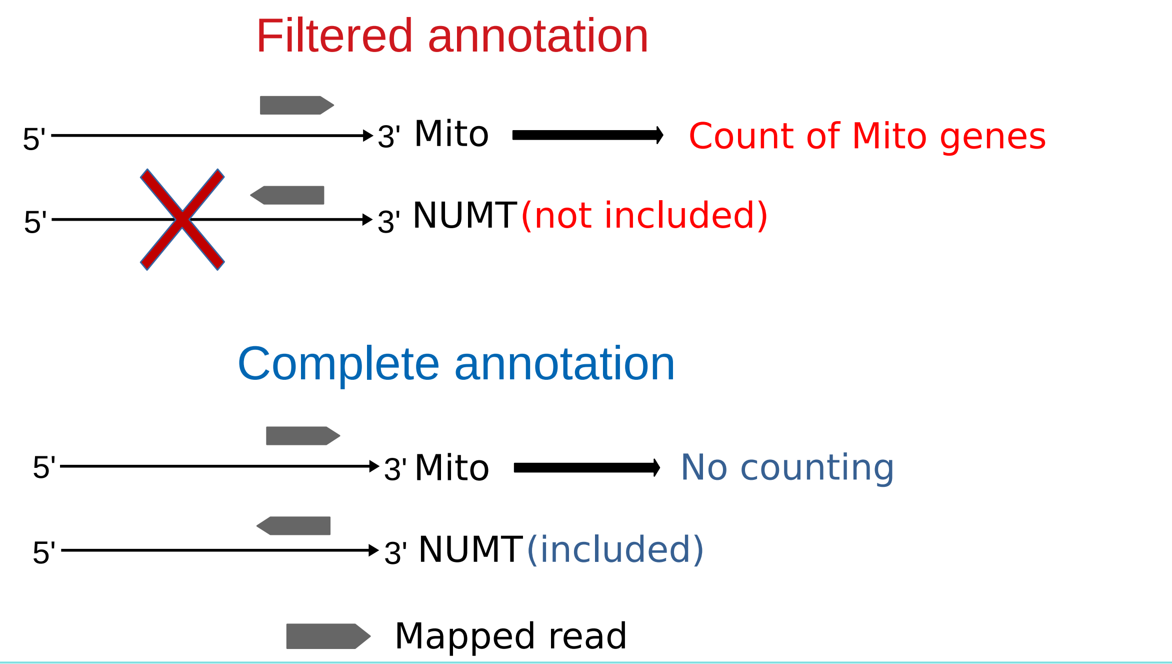

Supplement: giac001_Supplemental_Files [file giac001_supplemental_files.zip › Suppl_figure_7_supplementary_material.pdf]
