# Supplementary material for: Comparative analysis of common alignment tools for single-cell RNA sequencing
Source: Gigascience. 2022 Jan 27;11:giac001. doi: 10.1093/gigascience/giac001 (PMC8848315; doi:10.1093/gigascience/giac001)
Supplement: giac001_Supplemental_Files [file giac001_supplemental_files.zip › Suppl_Table_1_supplementary_material.pdf]

Technical Overview mapper

	Cell Ranger	STARsolo	Alevin	Kallisto
**Mapping scheme**	Exact alignment	Exact alignment	Pseudo mapping	Pseudo mapping
**Internal Mapper**	Star	Star	Salmon	Kallisto
**Reference**	Genome	Genome	Transcriptome + Genome	Transcriptome
**Supported sequence technology**	10X Chromium v1 – v3	10X Chromium v2;v3, Smart-seq, Drop-seq, inDrop	10x Chromium v2;v3, Drop-seq, Cel-seq, Cel-seq2, Quartz-seq2	10x Chromium v1 – v3, Cel-seq, Cel-seq2, Drop-seq, inDrops v1-v3, SCRB-Seq, SureCell
**Barcode correction**	1-Hamming distance based	1-Hamming distance based	Edit distance calculation	1-Hamming distance based
**Whitelisting**	Whitelist based	Whitelist based	Frequency based, no whitelist needed	Whitelist based
**Alternative Splicing detection**	no	yes	no	no
**UMI correction**	Two round correction by barcode, read count and annotation	Two round correction by barcode, read count and annotation	graph based correction	NA
**Index**	Suffix array	Suffix array	Colored De-Bruijn Graph	Colored De-Bruijn Graph
**Handling of multimapped reads**	discarded	discarded	Distributing read count between genes by EM-algorithm	discarded
**Output**	Matrix + Bam-File and summary file as html-file with primary results as well as clustering and DEG analysis	Gene count matrix and primary results summary	Gene count matrix ready for analysis	External software required to create gene count matrix
