# Supplementary material for: Comparative analysis of common alignment tools for single-cell RNA sequencing
Source: Gigascience. 2022 Jan 27;11:giac001. doi: 10.1093/gigascience/giac001 (PMC8848315; doi:10.1093/gigascience/giac001)
Supplement: giac001_Supplemental_Files [file giac001_supplemental_files.zip › Suppl_Table_2_supplementary_material.pdf]

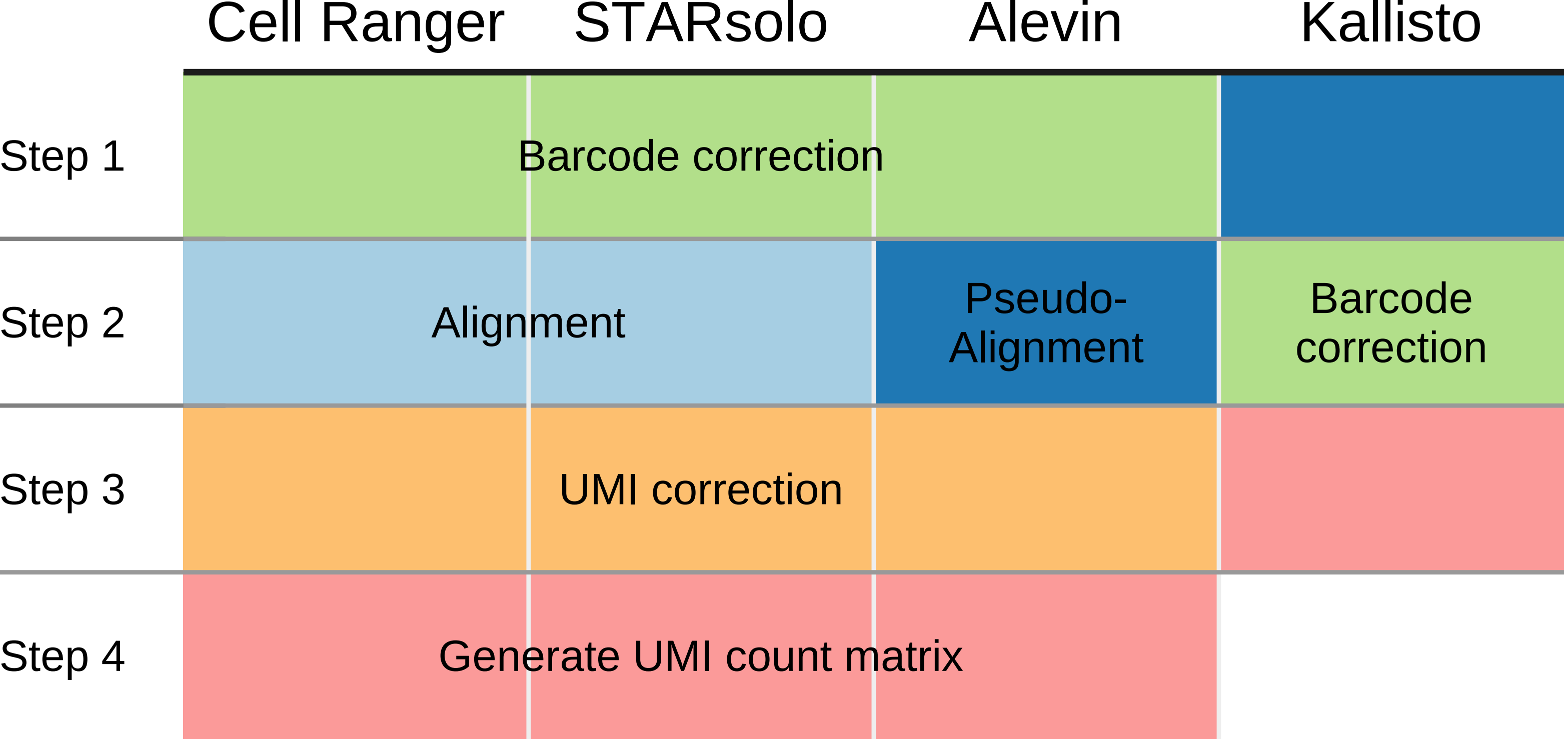
Cell Ranger
STARsolo
Alevin
Kallisto
Step 1
Barcode correction
Step 2
Alignment
Pseudo-Alignment
Barcode correction
Step 3
UMI correction
Step 4
Generate UMI count matrix
