# Supplementary material for: Comparative analysis of common alignment tools for single-cell RNA sequencing
Source: Gigascience. 2022 Jan 27;11:giac001. doi: 10.1093/gigascience/giac001 (PMC8848315; doi:10.1093/gigascience/giac001)
Supplement: giac001_Supplemental_Files [file giac001_supplemental_files.zip › Suppl_Table_3_supplementary_material.pdf]

Dataset	Sample name	10x version	Read count	Read length	Read Q30
**PBMC**	sample1	v3	96,285,389	91	95.5
	sample2		96,883,919		95.5
	sample3		95,767,327		95.3
	sample4		95,004,972		59.2
**Endothelial**	Brain	v2	238,554,858	97	85.90
	Heart		267,400,744		85.80
	Liver		237,695,938		85.20
	Muscle EDL		269,159,432		88.60
	Small Intestine		237,622,115		87.50
	Testis		268,113,285		86.70
	Colon		253,731,975		98.40
	Kidney		305,752,641		86.20
	Lung		263,905,964		86.90
	Muscle Soleus		269,848,594		87.60
	Spleen		269,739,092		88.50
**Cardiac**	MF17010	v2	121,537,000	98	82.7
	MF17013		138,238,205		83.5
	MF17014		143,726,852		78.5
	MF17015		148,925,993		78.4
	MF17016		169,668,778		81.1
	MF17017		383,452,065		84.2
	MF17018		378,754,335		81.9
**HF**	103837-001-001	v3	263,889,279	151	90.3
	103837-001-002		236,654,898		90.2
	103837-001-003		310,113,740		90.5
	103837-001-004		97,751,102		90.2
	103837-001-005		245,394,685		89.5
