# Supplementary material for: Comparative analysis of common alignment tools for single-cell RNA sequencing
Source: Gigascience. 2022 Jan 27;11:giac001. doi: 10.1093/gigascience/giac001 (PMC8848315; doi:10.1093/gigascience/giac001)
Supplement: giac001_Supplemental_Files [file giac001_supplemental_files.zip › Suppl_table_4_supplementary_material.pdf]

Cardiac

Fibroblasts	Endothelial-cells	Granulocytes	Monocytes	NKT-cells	B-cells	T-cells	Mural-cells
Lamc1	Ly6c1	S100a8	Dab2	Ccl5	Cd79a	Cd3g	Kcnj8
Pcsk6	Egfl7	S100a9	Adgre1	Nkg7	Ly6d	Lat	Vtn
Pdgfra	Gpihbp1	Slpi	Mgl2	Klrk1	Cd79b	Cd3e	Colec11
Entpd2	Cdh5	Csf3r	Mrc1	Klre1	H2-DMb2	Il7r	Steap4
Dpep1	Mgll	Hcar2	Hpgd	Klrd1	Ms4a1	Cd247	Abcc9
Adamts5	Slc9a3r2	Lmnb1	P2ry6	Ncr1	H2-Ob	Itk	Myo1b
Medag	Emcn	Retnlg	C3ar1	Ctsw	Fcmr	Icos	Cog7
Ms4a4d	Kdr	Clec4d	F13a1	Klrb1c	Ccr7	Trdc	P2ry14
Lamb1	Pecam1	Hp	Maf	Gzma	Bank1		Heyl
Tcf21	Rgcc	Hdc	Ms4a7	Gzmb	Cd55		Gnb4
Col1a1	Tie1		Cd14	Cd3d	Iglc2		Tagln
Dkk3	Cldn5		Fcgr1	Skap1	Iglc3		Mustn1
Tbx20	Eng		Ptprc	Lef1	Cd19		Myh11
Wif1			Lgals3	Tcf7	Pax5		Mylk
Frzb			Napsa		Iglc1		Pcp4l1
Meox1			Plbd1				Sncg
Prg4			Ccr2				Lmod1
Abi3bp			Rnase6				Des
Pdgfrl			Plac8				Pln
Mdk			Ifitm6				Nrip2
Gstm5			Naaa				
			Ear2				
			H2afy				

PBMC

T-cell 1 (CD4+)	Monocytes	NK-cells	B-cells	T-cell (CD8+)	Platelets	Dendritic cells
CD3G	MS4A7	GNLY	CD79A	CD8A	PPBP	FCER1A
LAT	CD14	CCL5	LY6D	CD8B	ITGA2B	CST3
CD3E	PTPRC	NKG7	CD79B	GZMK	PF4	IL3RA
IL7R	LYZ	KLRK1	MS4A1	CCL5	GP1BA	LILRA4
CD247	FCGR3A	KLRE1	FCMR	CD27	SELP	DNASE1L3
ITK		KLRD1	BANK1	THEMIS	TUBB1	RNASE6
ICOS		NCR1	CD55		ITGB3	
TRDC		CTSW	IGLC1		NCOA4	
CCR7		KLRB1C	IGLC2		RGS18	
		GZMA	IGLC3		TREML1	
		GZMB	CD19		CD9	
		PRF1	PAX5		NRGN	
					MYL9	
