# Supplementary material for: Comparative analysis of common alignment tools for single-cell RNA sequencing
Source: Gigascience. 2022 Jan 27;11:giac001. doi: 10.1093/gigascience/giac001 (PMC8848315; doi:10.1093/gigascience/giac001)
Supplement: giac001_Supplemental_Files [file giac001_supplemental_files.zip › Suppl_table_5_supplementary_material.pdf]

Cardiac		HF	
Control	29.92%	103837-001-001	75.00%
Day 1	17.71%	103837-001-002	52.57%
Day 3	16.61%	103837-001-003	24.86%
Day 5	21.57%	103837-001-004	17.15%
Day 7	23.59%	103837-001-005	51.94%
Day 14	21.31%		
Day 28	24.30%		
