# Supplementary material for: Comparative analysis of common alignment tools for single-cell RNA sequencing
Source: Gigascience. 2022 Jan 27;11:giac001. doi: 10.1093/gigascience/giac001 (PMC8848315; doi:10.1093/gigascience/giac001)
Supplement: giac001_Supplemental_Files [file giac001_supplemental_files.zip › Suppl_Table_6_supplementary_material.pdf]

Endothelial

Sample	% unique bc not in whitelist
Heart	1.49
Kidney	39.01
Liver	9.73
Brain	59.05
Colon	21.92
Lung	18.03
EDL	2.9
Soleus	5.2
Small intestine	3.97
Spleen	11.12
Testis	6.15

PBMC

Sample	% unique bc not in whitelist
N1	51.6
N2	51.9
N3	52.9
N4	54.7
