# Supplementary figures and images for: Comparative analysis of common alignment tools for single-cell RNA sequencing
Source: Gigascience. 2022 Jan 27;11:giac001. doi: 10.1093/gigascience/giac001 (PMC8848315; doi:10.1093/gigascience/giac001)

A

## Intersection of barcodes

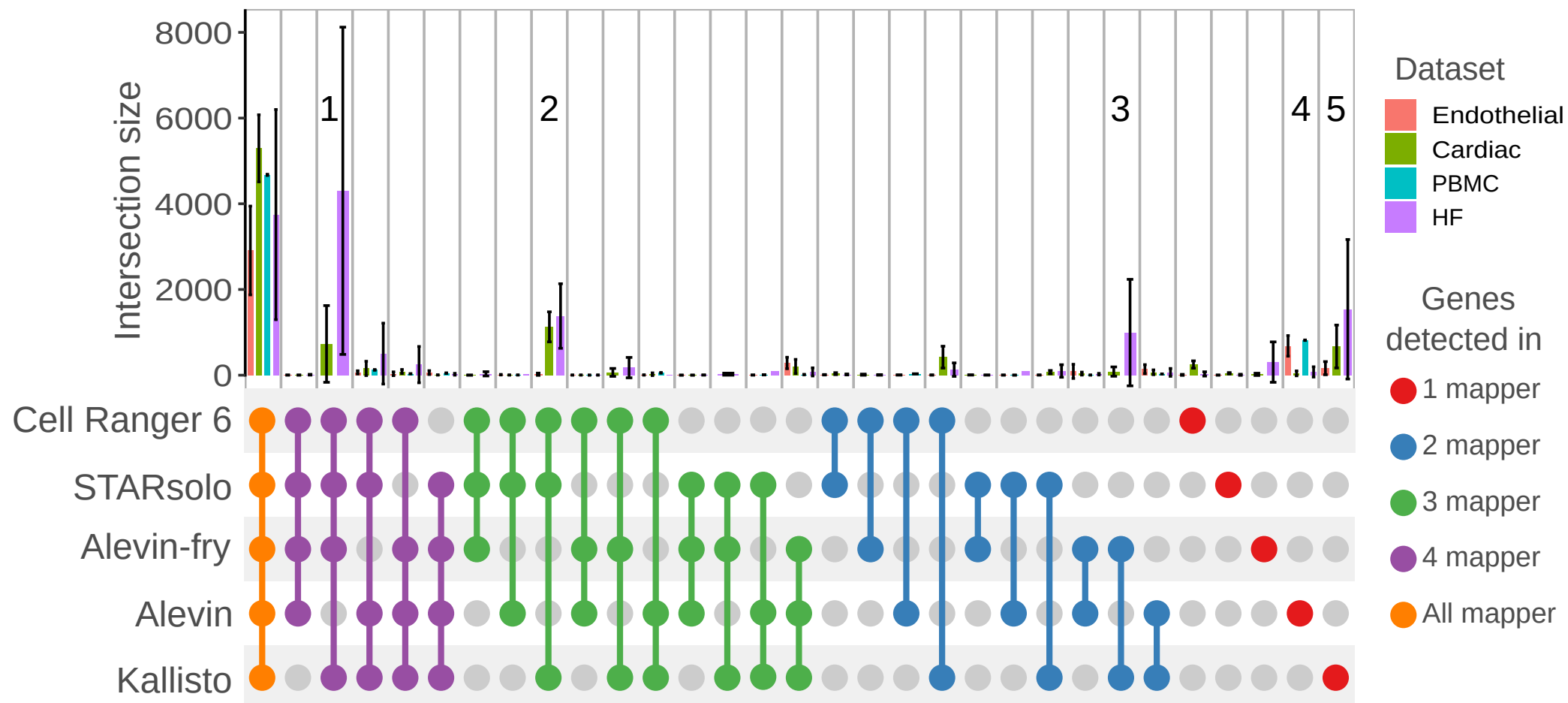

B

## Distribution of selected intersections

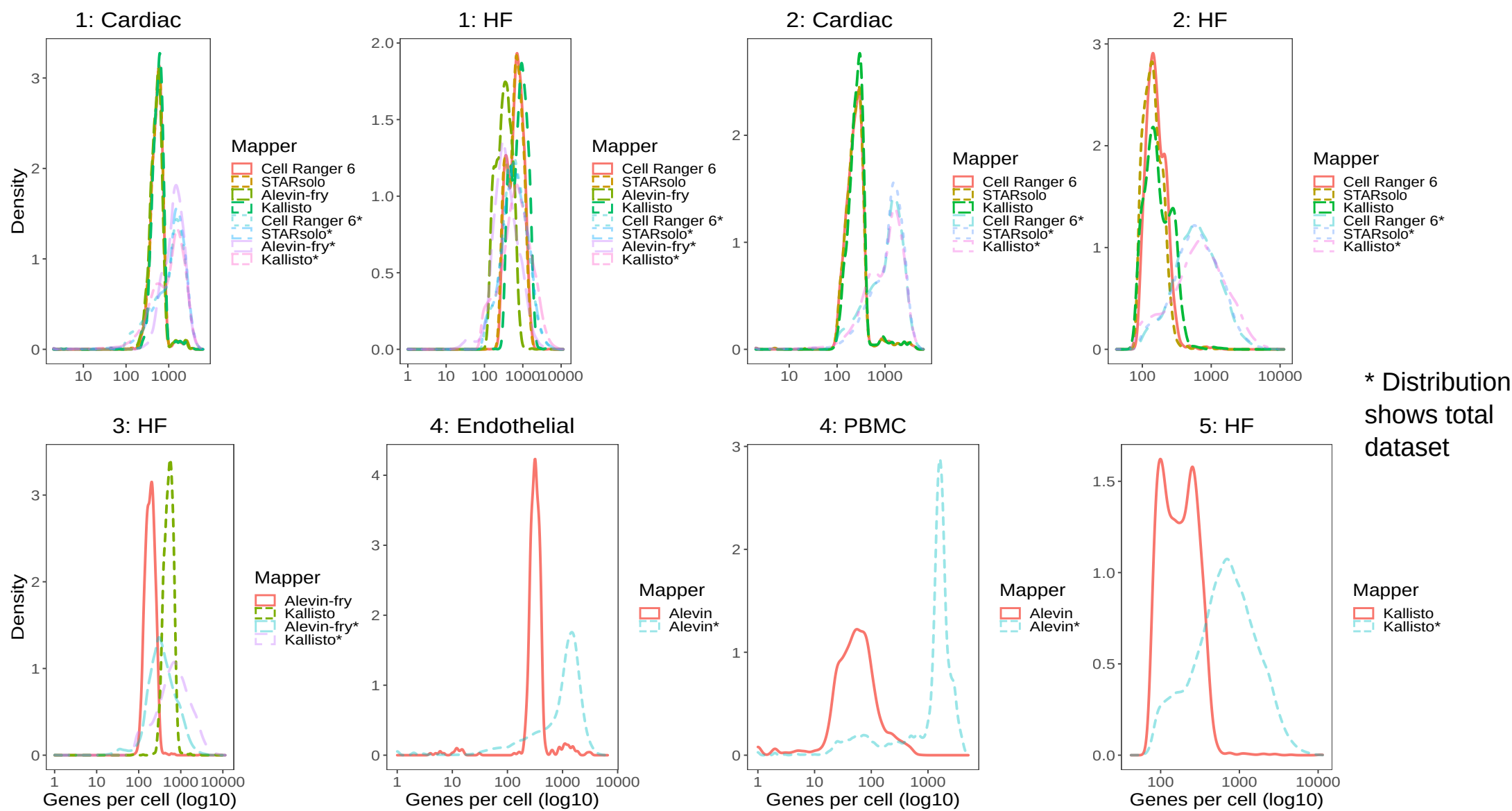

Supplement: giac001_Supplemental_Files [file giac001_supplemental_files.zip › Suppl_Figure_2_supplementary_material.pdf]

Number of biotypes (log10)

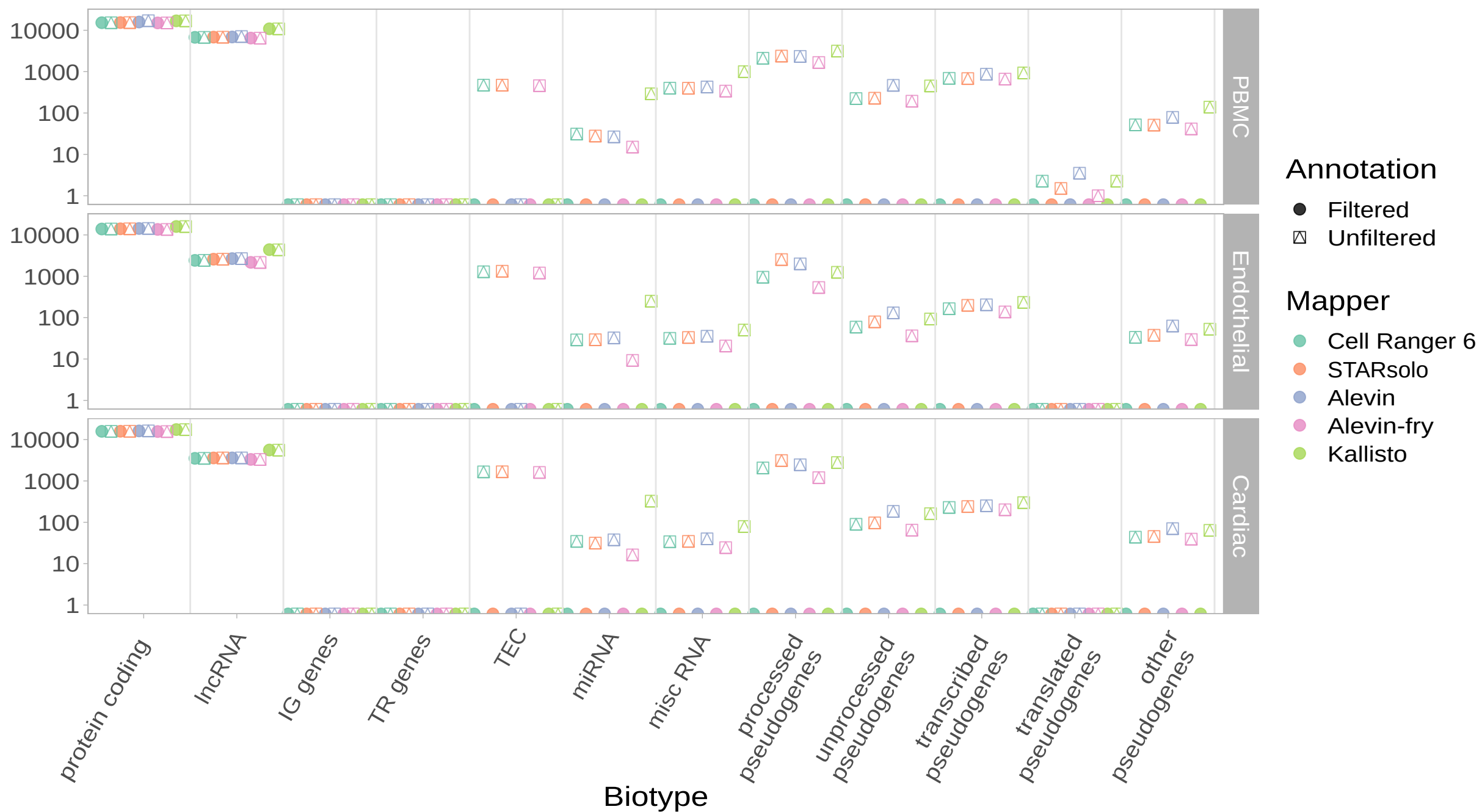

Supplement: giac001_Supplemental_Files [file giac001_supplemental_files.zip › Suppl_figure_3_supplementary_material.pdf]

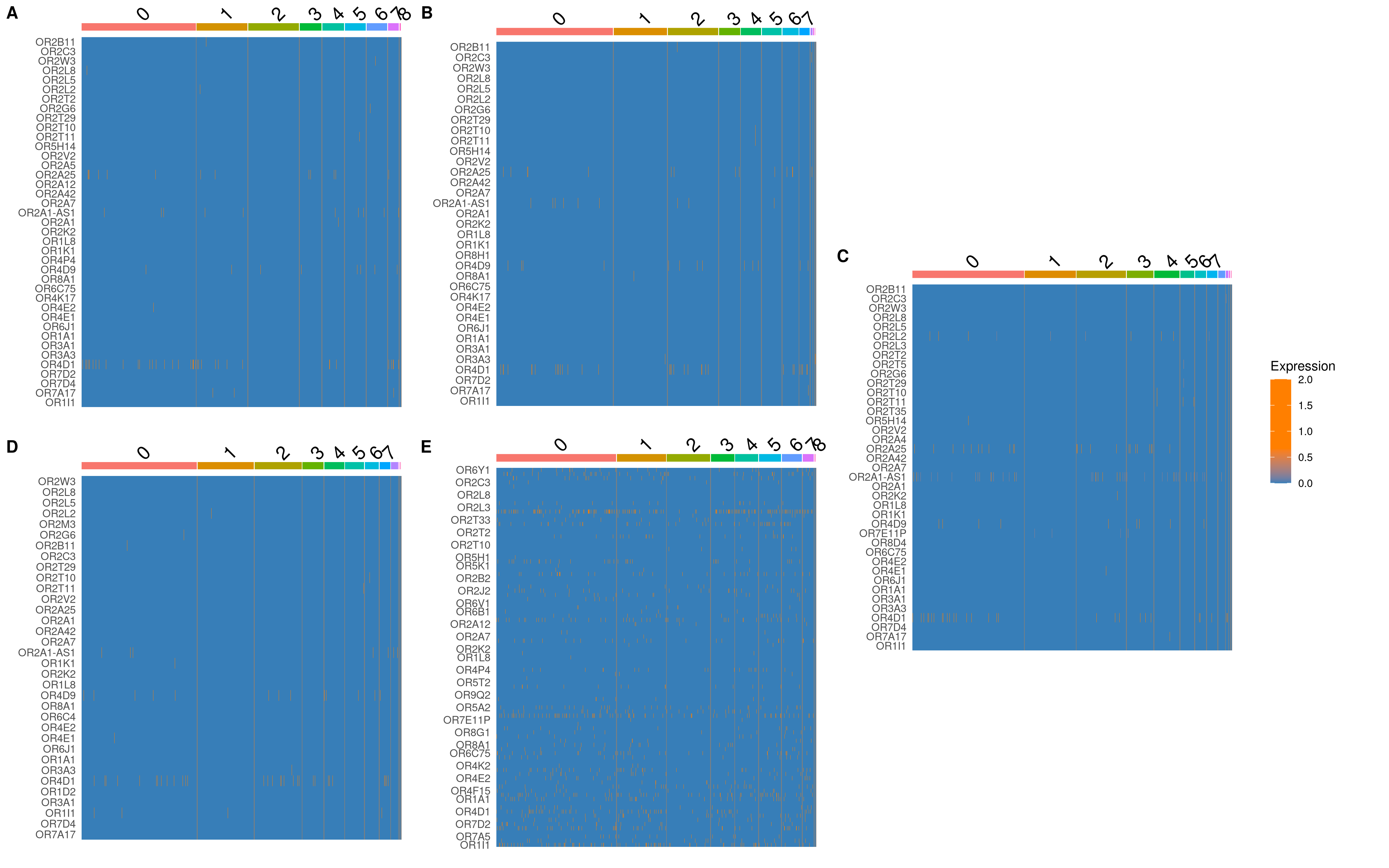

Supplement: giac001_Supplemental_Files [file giac001_supplemental_files.zip › Suppl_figure_4_supplementary_material.png]

# PBMC

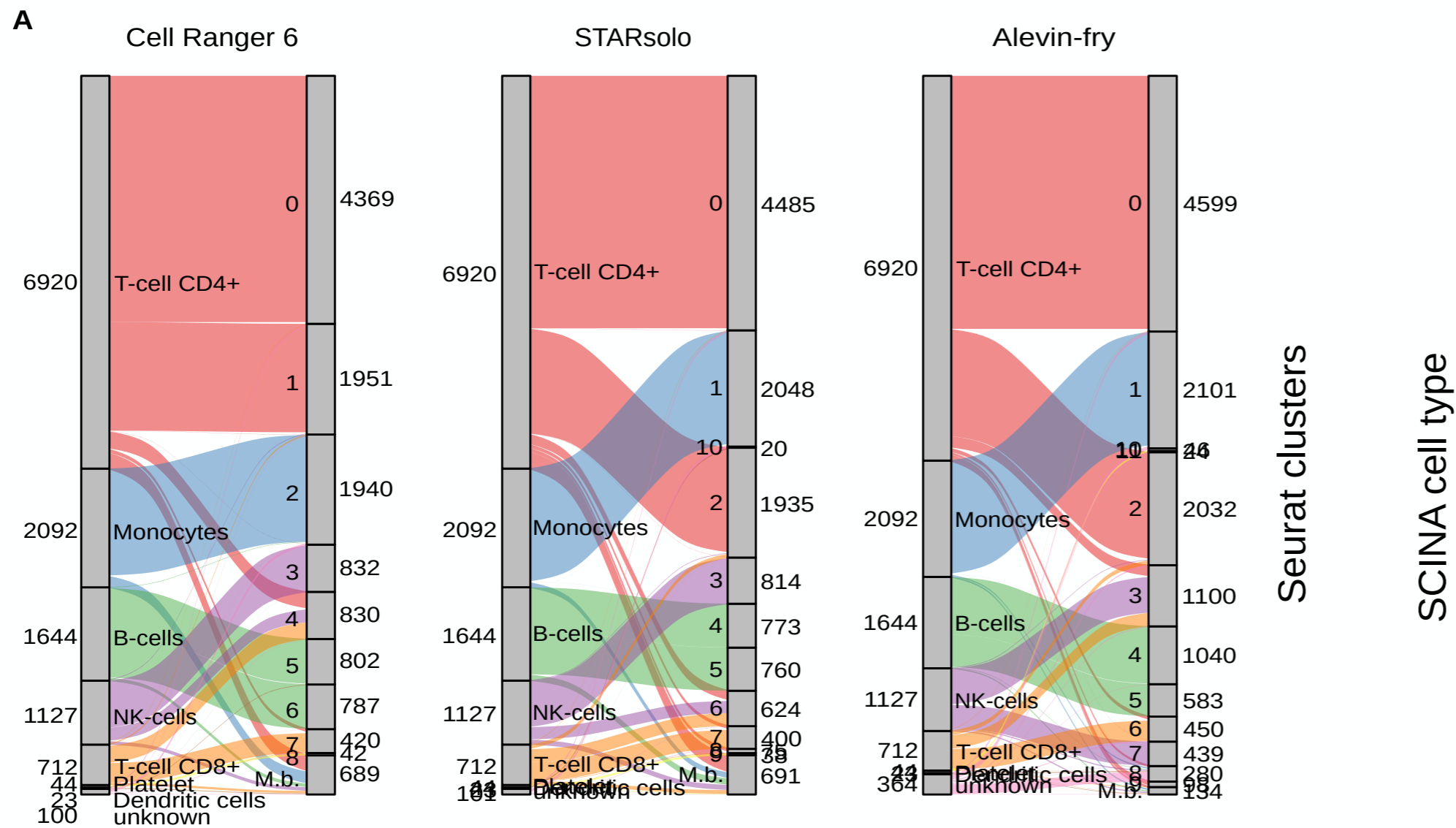

# Cardiac

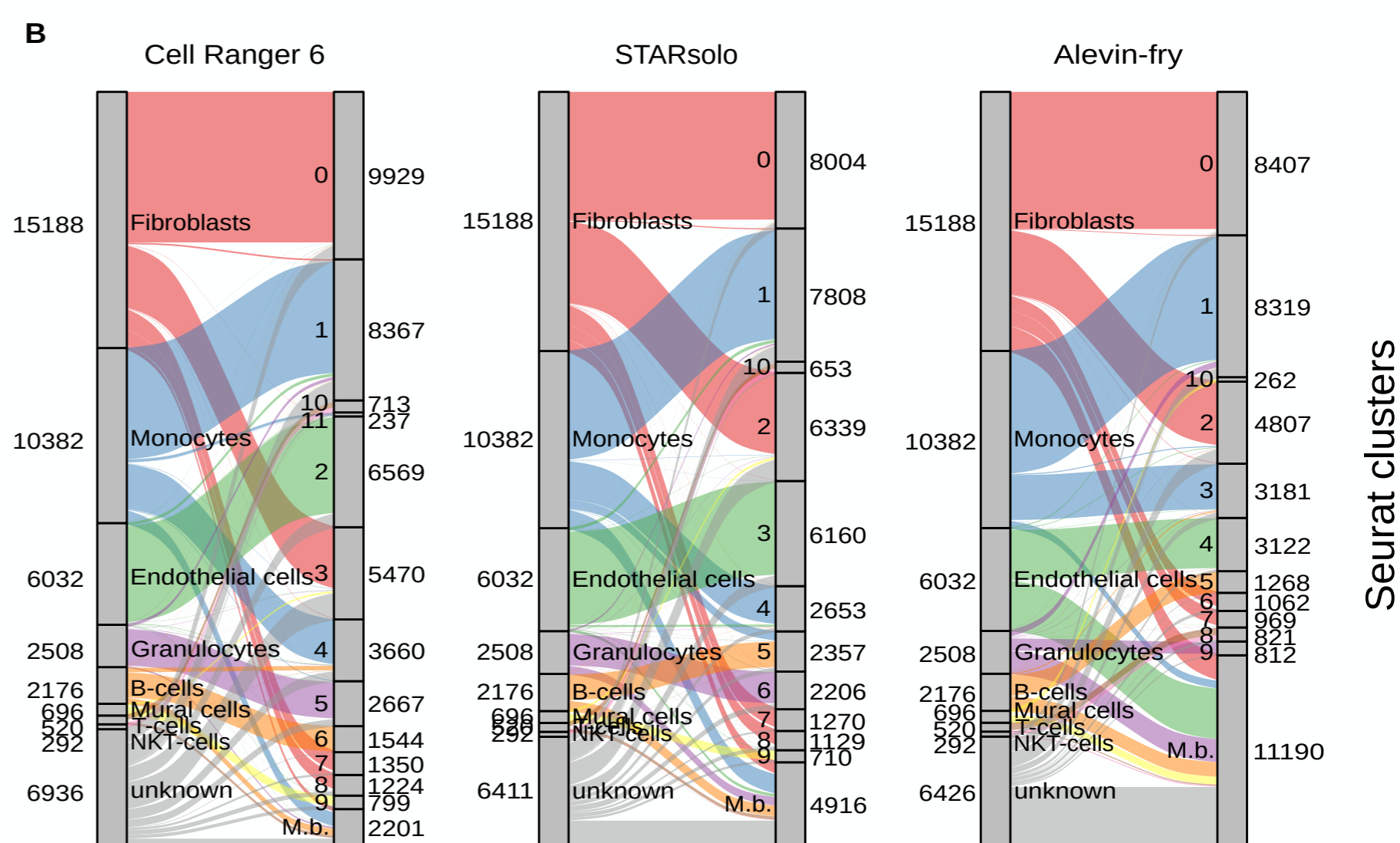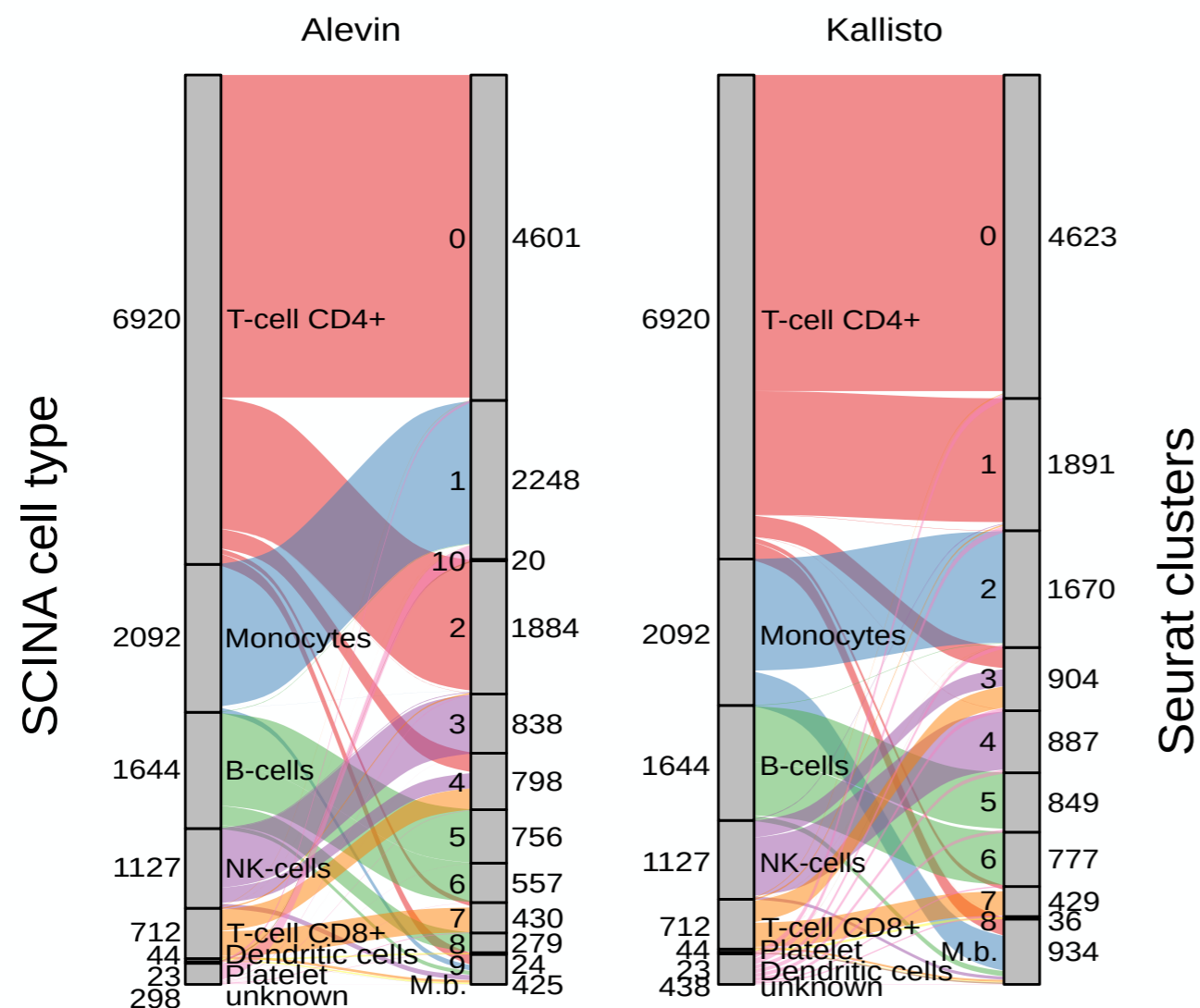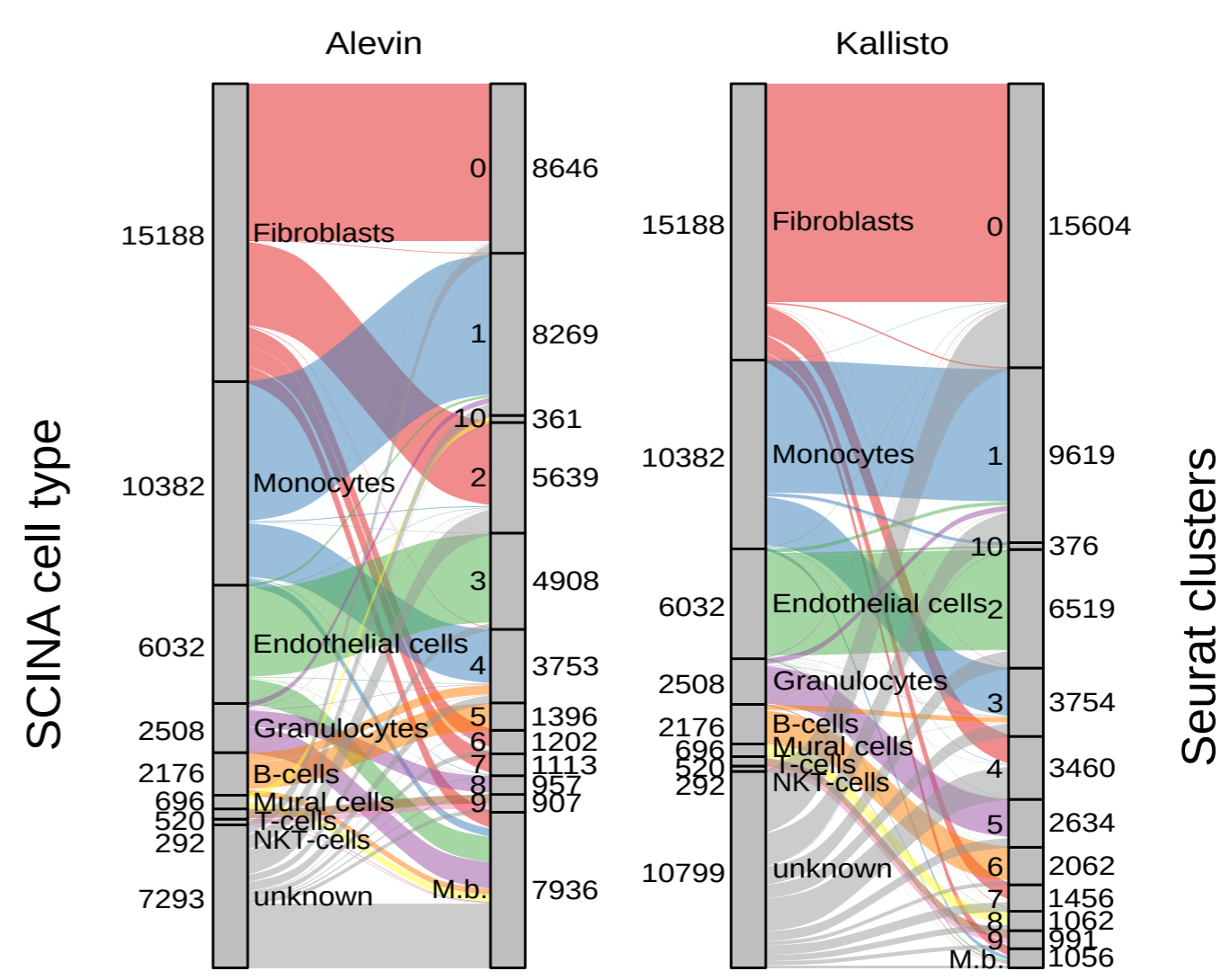

Supplement: giac001_Supplemental_Files [file giac001_supplemental_files.zip › Suppl_figure_5_supplementary_material.pdf]

**A****F1-score in PBMC**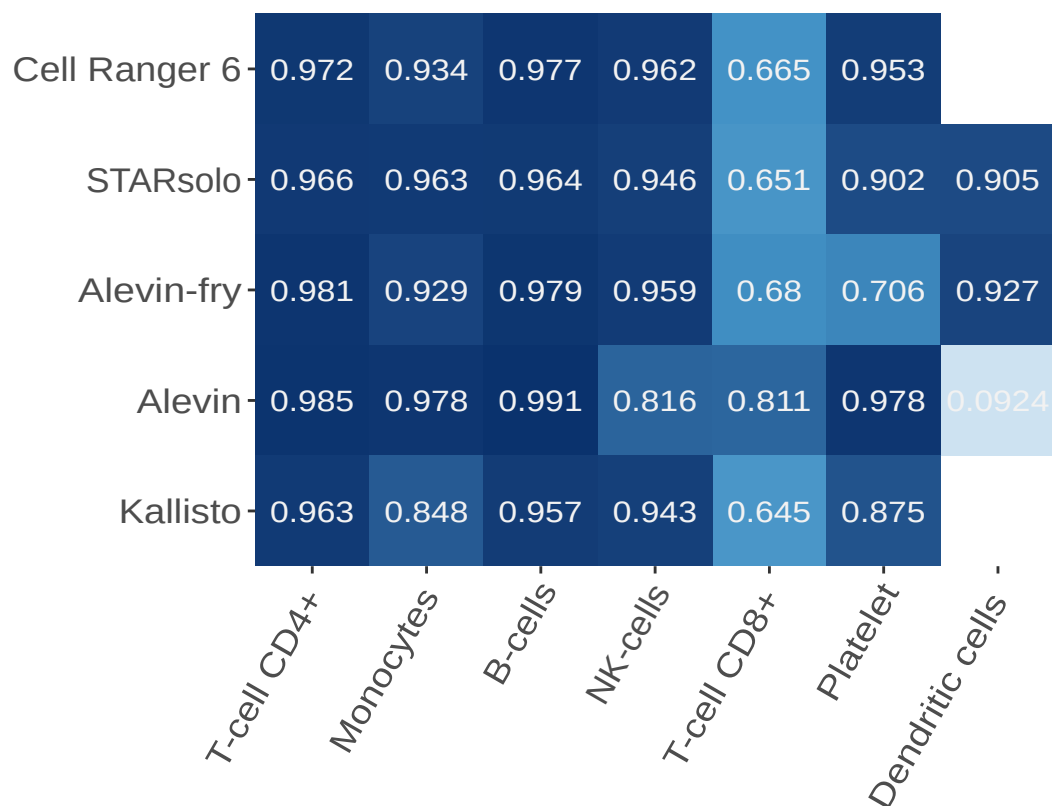**B****recall in PBMC**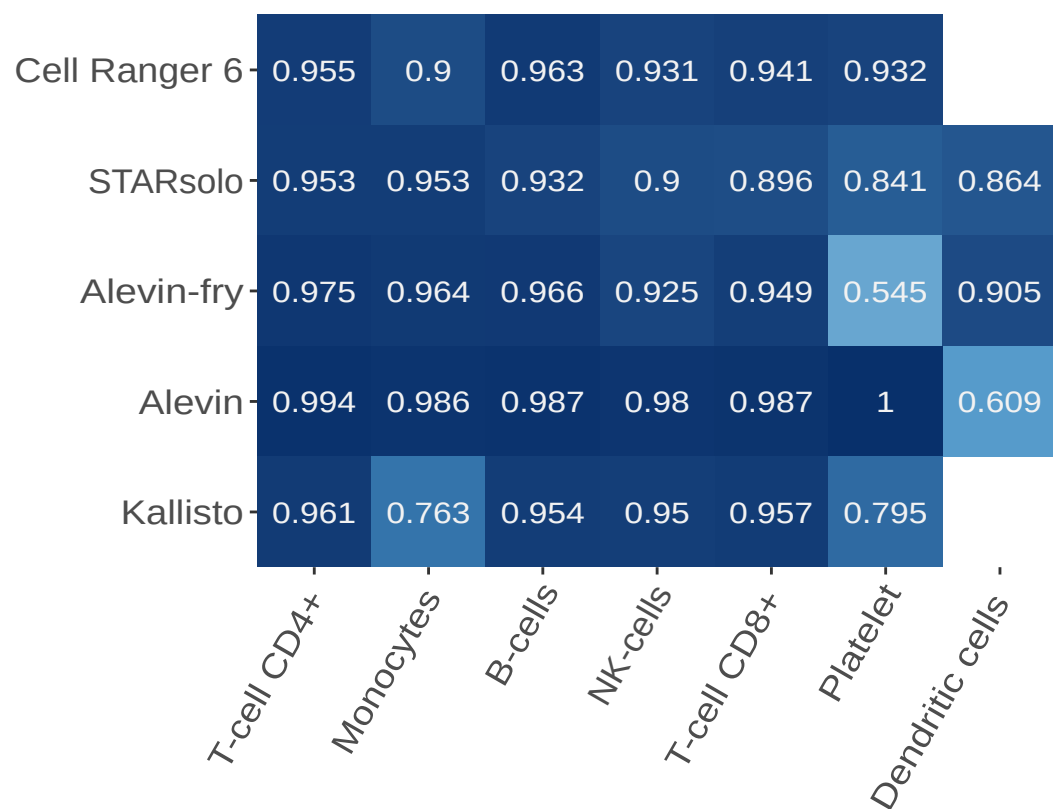**C****precision in PBMC**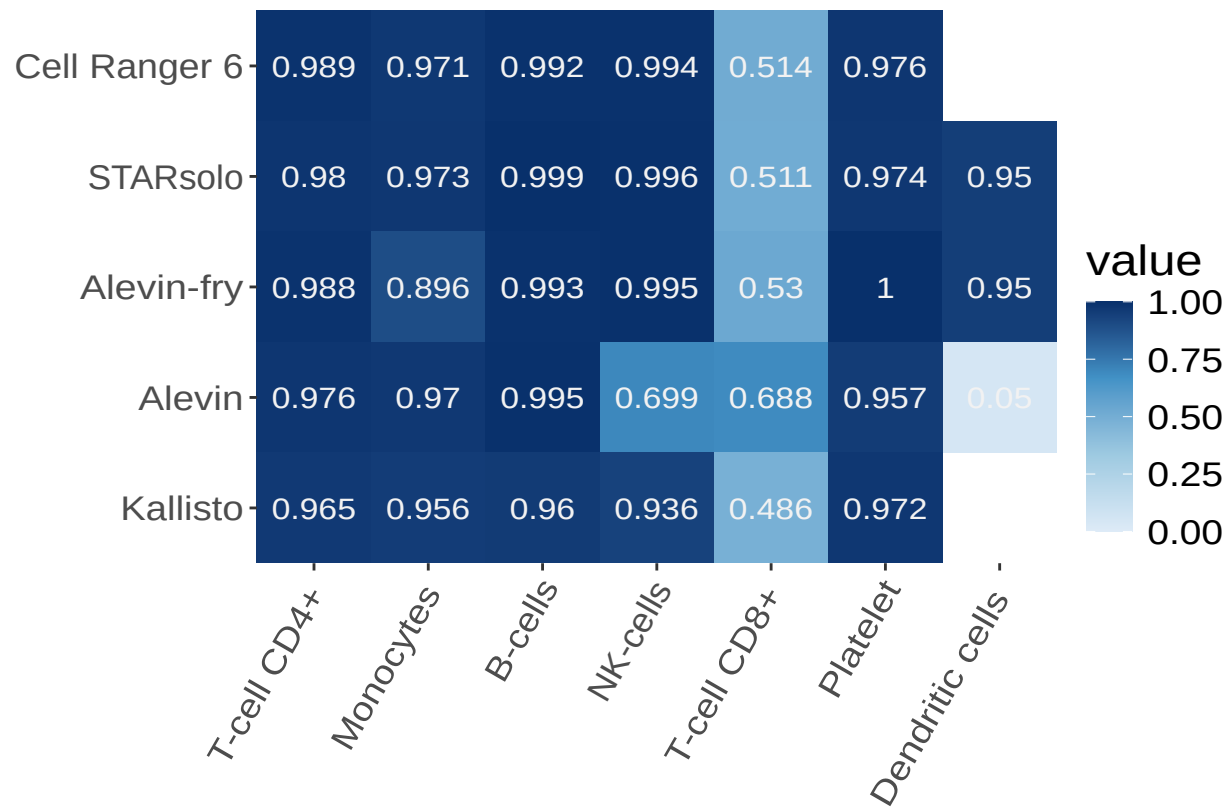**D****Recall in Cardiac**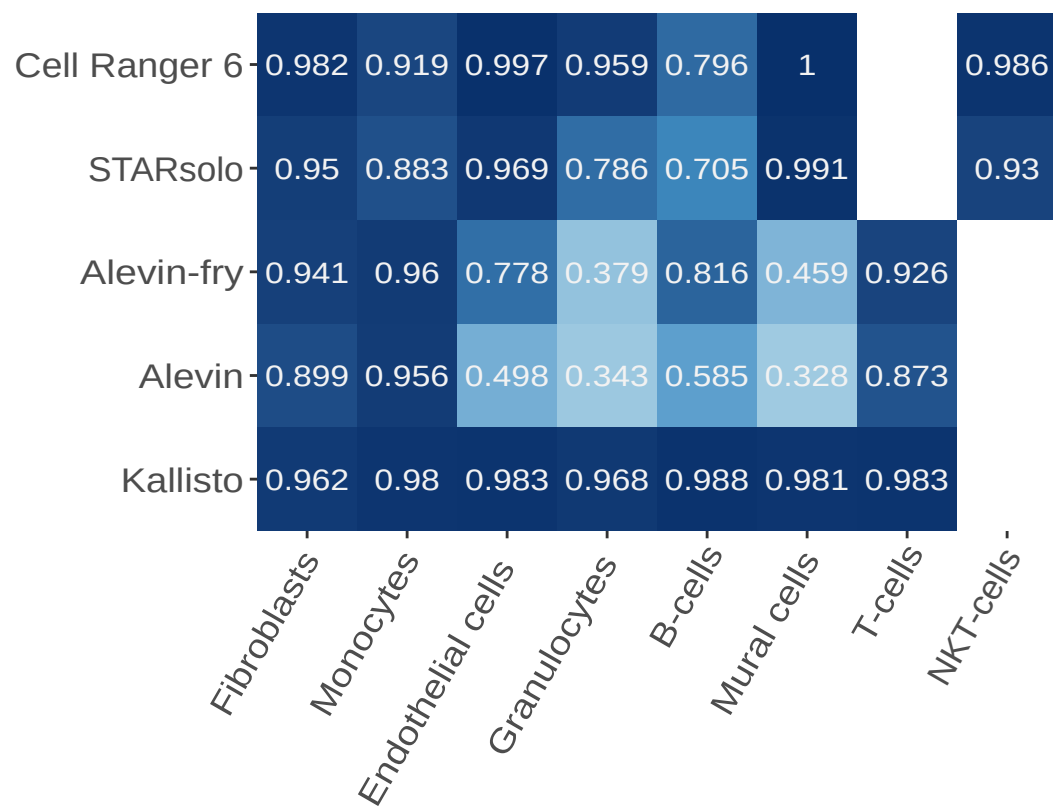**E****Precision in Cardiac**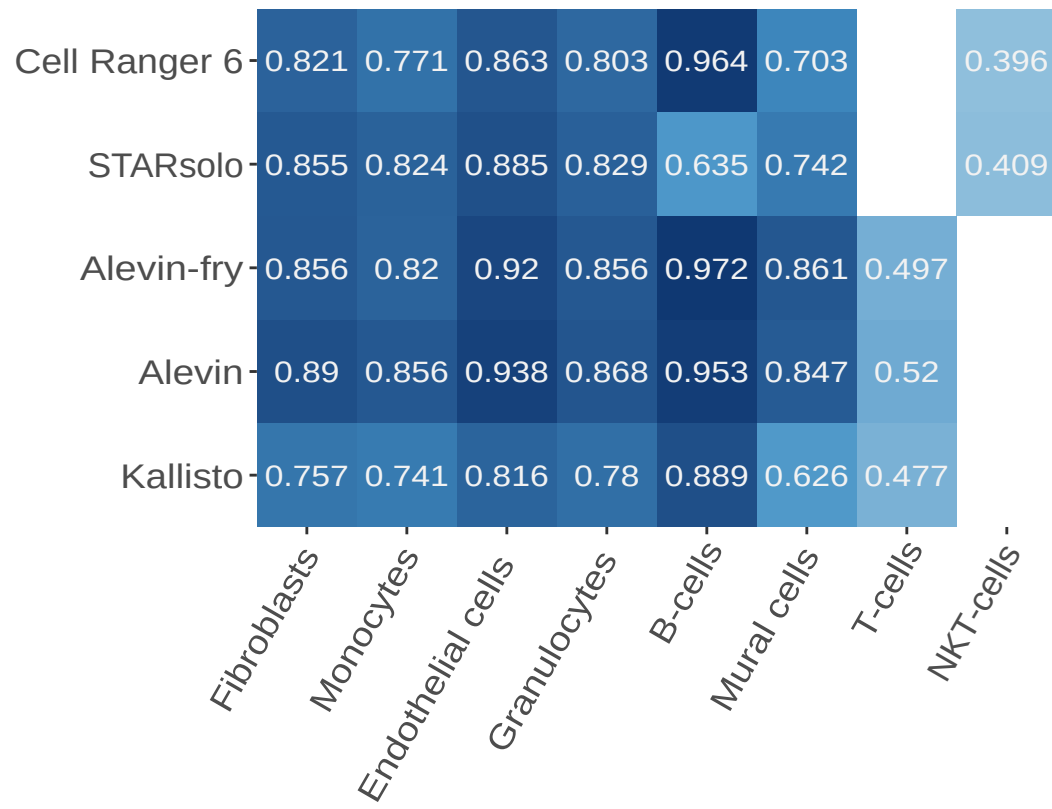

Supplement: giac001_Supplemental_Files [file giac001_supplemental_files.zip › Suppl_figure_6_supplementary_material.pdf]
