# Supplementary material for: Comparative analysis of common alignment tools for single-cell RNA sequencing
Source: Gigascience. 2022 Jan 27;11:giac001. doi: 10.1093/gigascience/giac001 (PMC8848315; doi:10.1093/gigascience/giac001)
Supplement: giac001_Response_to_Reviewer_Comments_Revision_2 [file giac001_response_to_reviewer_comments_revision_2.pdf]

Author's Response To Reviewer Comments

Close

Responds on Reviewer3

Reviewer 3

1.) I have one more comment about this version of the manuscript. I think that since alevin exposes multiple ways to run the alignment phase upstream of alevin-fry, including the sketch mode, a more apt performance comparison is either to include the alevin-fry pipeline in sketch mode, or if this isn't done, to make clear in the manuscript that this configuration is not included.

I would like to refer authors to Figure 6A of STAR-solo manuscript (https://www.biorxiv.org/content/10.1101/2021.05.05.442755v1.full.pdf) which exposes such a comparison, and which finds alevin-fry with sketch based alignment to be the fastest configuration overall. As such, drawing conclusion about the speed of the tools presented, without mentioning that certain configurations that optimize for speed have not been included, might be misleading to general readers who are not otherwise aware of the configurability of these tools.

We thank the reviewer for this valuable comment. As suggested we stated in the discussion section that the Alevin sketch mode was not used for the mapping processes performed with Alevin-fry. We changed Figure 5 accordingly and added the following sentence to the discussion section of the manuscript:

"With the option for selective alignment, which was used throughout this paper, Alevin-fry had a similar runtime than STARsolo. However Kaminov et. al. showed that the runtime decreases when using the pseudo-alignment algorithm (sketch mode) for Alevin-fry, yet this leads to a reduction in accuracy [https://www.biorxiv.org/content/10.1101/2021.05.05.442755v1.full.pdf] as mapping positions are not validated via alignment scoring [https://www.biorxiv.org/content/biorxiv/early/2021/08/26/2021.06.29.450377.full.pdf]."

Close
