# Supplementary material for: Comparative analysis of common alignment tools for single-cell RNA sequencing
Source: Gigascience. 2022 Jan 27;11:giac001. doi: 10.1093/gigascience/giac001 (PMC8848315; doi:10.1093/gigascience/giac001)
Supplement: giac001_Reviewer_1_Report_Original_Submission — Bo Li, Ph.D. -- 6/7/2021 Reviewed [file giac001_reviewer_1_report_original_submission.pdf]

Reviewer Report

Title: Comparative Analysis of common alignment tools for single cell RNA sequencing

Version: Original Submission **Date:** 6/7/2021

Reviewer name: Bo Li, Ph.D.

Reviewer Comments to Author:

Single-cell RNA-seq has revolutionized our abilities of investigating cell heterogeneity in complex tissue. Generating a high-quality gene count matrix is a critical first step for single-cell RNA-seq data analysis. Thus, a detailed comparison and benchmarking of available gene-count matrix generation tools, such as the work described in this manuscript, is a pressing need and has the potential to benefit the general community.

Although this work has a great potential, the benchmarking efforts described in the manuscript are not comprehensive enough to justify its publication at GigaScience unless the authors address my following major and minor concerns.

Major concerns:

1) The authors should discuss related benchmarking efforts and the differences between previous work and this manuscript in the Background section instead of the Discussion section. For example, Du et al. 2020 G3: Genes, Genomics, Genetics. and Booeshaghi & Pacther bioRxiv 2021 should be mentioned and discussed in the Background section. In addition, STARsolo manuscript (https://www.biorxiv.org/content/10.1101/2021.05.05.442755v1), which contains a comprehensive comparison of CellRanger, STARsolo, Alevin and Kallisto-Bustools should be cited and discussed. Zakeri et al. 2021 bioRxiv (https://www.biorxiv.org/content/10.1101/2021.02.10.430656v1) should also be included and discussed in the Background section.

2) Benchmark with latest versions of the software. The choice of Cell Ranger, STARsolo, Alevin and Kallisto-BUStools is good because they are four major gene count matrix generation tools. However, I urge the authors also include CellRanger v6 and Alevin-fry (Alevin_sketch/Alevin_partial-decoy/Alevin_full-decoy, see STARsolo manuscript), which are currently lacking, into their benchmarking efforts. The authors may also consider add STARsolo_sparseSA into the benchmark. Since single-cell RNA-seq tool development is a fast-evolving field, benchmarking of the up-to-date versions of tools is super critical for a benchmarking paper.

3) Conclusions. The authors summarized the observed differences between tools based on the benchmarking results. This is good but very helpful for end-users. I recommend the authors to emphasize their recommendations for end-users more clearly in the discussion/results section. For example, do the authors recommend one tool over the others under certain circumstances? If so, which tool and which circumstance and why? I like Figure 5 a lot and hope the authors can summarize this figure better in the manuscript.

4) This manuscript concluded that differential expression (DEG) results showed no major differences among the alignment tools (Figure 4). However, the STARsolo manuscript suggested DEG results are strongly influenced by quantification tools (Sec. 2.6, Figure 5). Please explain this discrepancy.

5) This manuscript suggested simulated data is not as helpful as real data. However, the STARsolo manuscript reported drastic differences between tools using simulated data. Please comment on this discrepancy.
6) I have big concerns regarding the filtered vs. unfiltered annotation comparison. In particular for pseudogenes, we know that many of them are merely transcribed or lowly transcribed. As a result, many of these pseudogenes would not be captured by the single-cell RNA-seq protocol. At the same time, because these pseudogenes share sequence similarities with functional genes, they would bring trouble for read mapping. This is one of the main reasons for using a carefully filtered annotation. Actually, whether and how to filter annotation is in active debate in big cell atlas consortia such as Human Cell Atlas. Thus, I would be super careful about describing results comparing filtered vs. unfiltered annotation. For example, in Suppl. Figure 8D, there are 6 mitochondrial genes that have 100% sequence similarity to their corresponding pseudogenes. It is impossible to distinguish if a read comes from a gene or a pseudogene for these 6 genes and it is also not necessary --- the transcribed RNA should also be exactly the same. Thus, I encourage the authors remove their pseudogenes from the annotation and I suspect the mouse data results should look similar to the human data in the Suppl. Figure 8A.
7) The endothelial dataset was only run on CellRanger 3 because the UMI sequence is one base shorter. Could the authors augment the UMI sequence with one constant base and run this dataset through CellRanger 4/5/6?
8) I think it is more appropriate to call the tools benchmarked as "gene count matrix generation tools" instead of "alignment tools".
Minor concerns:
1) The Suppl Table 2 mentioned in the main text corresponds to Suppl. Table 3 in the attachment. In addition, there is no reference to Suppl Table 2.
2) Suppl Table 3 PBMC, why do I see endothelial cell markers in PBMC dataset?
3) Suppl Figure 7 is never referenced in the main text.
4) Suppl Figure 8D is never referenced in the main text.

Methods

Are the methods appropriate to the aims of the study, are they well described, and are necessary controls included? Choose an item.

Conclusions

Are the conclusions adequately supported by the data shown? Choose an item.

Reporting Standards

Does the manuscript adhere to the journal’s guidelines on [minimum standards of reporting?]() Choose an item.

Choose an item.

Statistics

Are you able to assess all statistics in the manuscript, including the appropriateness of statistical tests used? Choose an item.

Quality of Written English

Please indicate the quality of language in the manuscript: Choose an item.

Declaration of Competing Interests

Please complete a declaration of competing interests, considering the following questions:

- Have you in the past five years received reimbursements, fees, funding, or salary from an organisation that may in any way gain or lose financially from the publication of this manuscript, either now or in the future?
- Do you hold any stocks or shares in an organisation that may in any way gain or lose financially from the publication of this manuscript, either now or in the future?
- Do you hold or are you currently applying for any patents relating to the content of the manuscript?
- Have you received reimbursements, fees, funding, or salary from an organization that holds or has applied for patents relating to the content of the manuscript?
- Do you have any other financial competing interests?
- Do you have any non-financial competing interests in relation to this paper?

If you can answer no to all of the above, write 'I declare that I have no competing interests' below. If your reply is yes to any, please give details below.

N/A

I agree to the open peer review policy of the journal. I understand that my name will be included on my report to the authors and, if the manuscript is accepted for publication, my named report including any attachments I upload will be posted on the website along with the authors' responses. I agree for my report to be made available under an Open Access Creative Commons CC-BY license (http://creativecommons.org/licenses/by/4.0/). I understand that any comments which I do not wish to be included in my named report can be included as confidential comments to the editors, which will not be published.

Choose an item.

To further support our reviewers, we have joined with Publons, where you can gain additional credit to further highlight your hard work (see: https://publons.com/journal/530/gigascience). On publication of this paper, your review will be automatically added to Publons, you can then choose whether or not to claim your Publons credit. I understand this statement.

Yes Choose an item.
