# Supplementary material for: Comparative analysis of common alignment tools for single-cell RNA sequencing
Source: Gigascience. 2022 Jan 27;11:giac001. doi: 10.1093/gigascience/giac001 (PMC8848315; doi:10.1093/gigascience/giac001)
Supplement: giac001_Reviewer_1_Report_Revision_1 — Bo Li, Ph.D. -- 10/25/2021 Reviewed [file giac001_reviewer_1_report_revision_1.pdf]

Reviewer Report

Title: Comparative Analysis of common alignment tools for single cell RNA sequencing

Version: Revision 1 **Date:** 10/25/2021

Reviewer name: Bo Li, Ph.D.

Reviewer Comments to Author:

The authors did a very great job addressing my concerns. I can foresee this manuscript as a great benchmarking paper for the community. However, It is still important for the authors to clarify on the following questions before a publication:

1. In last paragraph of discussion, we have "If high-quality cell counts need to be obtained, Alevin appears to be the most suitable method, as average gene counts 23 are high- and poor-quality barcodes are seldom reported."
This statement is super confusing because in Figure 5, the authors recommended Alevin-Fry over Alevin for all cases? In addition, I do not see why if high-quality cell counts needed, Alevin is necessarily the best tool.
2. The last sentence in the same paragraph - "For very large projects with a high number of samples, pseudo-alignment tools such as Alevin-fry or Kallisto can be advantageous in terms of runtime and storage efficiency, at the cost of a slight reduction in accuracy."
This statement is not well-supported by the data. Based on Figure 1, the runtime of STARsolo and Alevin-fry are very similar. If the authors want to claim pseudoalignment methods are advantageous in terms of runtime, I think only Kallisto should be mentioned. However, the authors also raised concerns regarding Kallisto's accuracy. So in this case, I am not sure if it is "at the cost of a slight reduction in accuracy".
3. STARsolo also provide multi-mapping reads assignment: https://github.com/alexdobin/STAR/blob/master/docs/STARsolo.md#multi-gene-reads. Please adjust Figure 5 accordingly.

Methods

Are the methods appropriate to the aims of the study, are they well described, and are necessary controls included? Choose an item.

Conclusions

Are the conclusions adequately supported by the data shown? Choose an item.

Reporting Standards

Does the manuscript adhere to the journal's guidelines on [minimum standards of reporting?]() Choose an item.

Choose an item.

Statistics

Are you able to assess all statistics in the manuscript, including the appropriateness of statistical tests used? Choose an item.

Quality of Written English

Please indicate the quality of language in the manuscript: Choose an item.

Declaration of Competing Interests

Please complete a declaration of competing interests, considering the following questions:

- Have you in the past five years received reimbursements, fees, funding, or salary from an organisation that may in any way gain or lose financially from the publication of this manuscript, either now or in the future?
- Do you hold any stocks or shares in an organisation that may in any way gain or lose financially from the publication of this manuscript, either now or in the future?
- Do you hold or are you currently applying for any patents relating to the content of the manuscript?
- Have you received reimbursements, fees, funding, or salary from an organization that holds or has applied for patents relating to the content of the manuscript?
- Do you have any other financial competing interests?
- Do you have any non-financial competing interests in relation to this paper?

If you can answer no to all of the above, write 'I declare that I have no competing interests' below. If your reply is yes to any, please give details below.

I declare that I have no competing interests.

I agree to the open peer review policy of the journal. I understand that my name will be included on my report to the authors and, if the manuscript is accepted for publication, my named report including any attachments I upload will be posted on the website along with the authors' responses. I agree for my report to be made available under an Open Access Creative Commons CC-BY license (http://creativecommons.org/licenses/by/4.0/). I understand that any comments which I do not wish to be included in my named report can be included as confidential comments to the editors, which will not be published.

Choose an item.

To further support our reviewers, we have joined with Publons, where you can gain additional credit to further highlight your hard work (see: https://publons.com/journal/530/gigascience). On publication of this paper, your review will be automatically added to Publons, you can then choose whether or not to claim your Publons credit. I understand this statement.

Yes Choose an item.
