# Supplementary material for: Comparative analysis of common alignment tools for single-cell RNA sequencing
Source: Gigascience. 2022 Jan 27;11:giac001. doi: 10.1093/gigascience/giac001 (PMC8848315; doi:10.1093/gigascience/giac001)
Supplement: giac001_Reviewer_2_Report_Original_Submission — Serghei Mangul -- 6/12/2021 Reviewed [file giac001_reviewer_2_report_original_submission.pdf]

Reviewer Report

Title: Comparative Analysis of common alignment tools for single cell RNA sequencing

Version: Original Submission **Date:** 6/12/2021

Reviewer name: Serghei Mangul

Reviewer Comments to Author:

1 -- Abstract contains. Confusing terminology, for example became available can be replaced by developed.
2 -- Also analyzed several data sets, can be replaced by benchmarking to clear indicate that that refers to benchmarking rather than analysis. Some terminology needs to be explained. For example, white listing should be defined
3 -- KALISTO is not alignment tool in a proper sense, as it doesn't report position of the read insteadonly the transcriptof origin. Instead, this is pseudo alignment. Alignment needs to be defined, or word pseudoalignment used
4 -- How the ground truth or gold standard was defined ? Is the assumption of the paper that the tool with the highest number of mapped reads perform the best?
This needs to be explained in the introduction.
5 -- In general. I read alignment is artificial rather than biological problem, so that molecular gold standard cannot be defined. See for example
https://www.nature.com/articles/s41467-019-09406-4. It would be helpful to explain this upfront when talking about gold standard and cite this.
6 -- It is unclear how the tools were selected. What was the reasoning to select only 4 tools and how do offer know that those tools are common? For the complete list of RNA-based alignment tools author can refer to
https://arxiv.org/abs/2003.00110
A reasonable criteria to select would be to take the tools, which are available, for example, in bioconda, which will make installing those tools easy. However, randomly selecting tools is not acceptable. For example, why the SALMON was not included. However, KALISTO was included.
7 --
Language of the paper needs to be improved, for example, in the background section the word great was used, which can be replaced by a more appropriate scientific wording.
8--
More explanation needs to be provided for cell ranger. Is it essentially the wrapper around the star? Does it have any novel Algorithms or software development involved?
9-- Needs me to explain why they chose only 10x genomics among the available single cell platforms.
10--
And the annotations indeed may influence, the alignment when they are provided for alignment tools. is every alignment tool able to take custom annotations?The paper is lacking the Figure providing results on which annotation performs the best for a given data sent.

11--
Datasets and reference genomes section
Gold standard data sets are not reported. It was not clear if the paper is having such data set or such data set is missing in case such data set, is missing. How the authors are able to say which read alignment tool performs the best ?
12--
The paper contains a single human sample. Any particular reason for that? The paper would benefit from having multiple human samples as a as it was done for the mouse. Did the authors performed a systematic search to identify as many single cell sample as possible. If not, that will be desirable.
13 --
Was that 10x data human data only available on 10x website, and not available on SRA or Geo
14 --
Paper provides a GitHub link with data sets and the code used for this analysis. Does the GitHub has also the BAM files?
If not, those needs to be uploaded. Additionally is the code and summary data behind the figures provided?
15 --
Results section, the beginning of results section would benefit with the short description of the datasets, for example.
How many samples were in total? What was the read length for each sample? what was the number of reads for each sample? Was a different. So providing the mean and the variance can be helpful.
16 --
In general, figures needs to be improved in terms of visualization. It's very hard to understand what are the figures are trying to convey. For example, figure 2 is absolutely impossible to understand. And also, what is the purpose of that figure is also unclear? The same for the figure 3 It's very busy, figure. However, what it is trying to convey? It's hard to know.
17 --
Figure 4 is also very hard to understand. So maybe making the log scale can improve.
What is the X axis, for example, that's unclear those details. And in general figures needs to be improved.
18 -- in general figures needs to be visually understandable and and more effective.

Methods

Are the methods appropriate to the aims of the study, are they well described, and are necessary controls included? Choose an item.

Conclusions

Are the conclusions adequately supported by the data shown? Choose an item.

Reporting Standards

Does the manuscript adhere to the journal's guidelines on [minimum standards of reporting?] Choose an item.

Choose an item.

Statistics

Are you able to assess all statistics in the manuscript, including the appropriateness of statistical tests used? Choose an item.

Quality of Written English

Please indicate the quality of language in the manuscript: Choose an item.

Declaration of Competing Interests

Please complete a declaration of competing interests, considering the following questions:

- Have you in the past five years received reimbursements, fees, funding, or salary from an organisation that may in any way gain or lose financially from the publication of this manuscript, either now or in the future?
- Do you hold any stocks or shares in an organisation that may in any way gain or lose financially from the publication of this manuscript, either now or in the future?
- Do you hold or are you currently applying for any patents relating to the content of the manuscript?
- Have you received reimbursements, fees, funding, or salary from an organization that holds or has applied for patents relating to the content of the manuscript?
- Do you have any other financial competing interests?
- Do you have any non-financial competing interests in relation to this paper?

If you can answer no to all of the above, write 'I declare that I have no competing interests' below. If your reply is yes to any, please give details below.

I declare that I have no competing interests

I agree to the open peer review policy of the journal. I understand that my name will be included on my report to the authors and, if the manuscript is accepted for publication, my named report including any attachments I upload will be posted on the website along with the authors' responses. I agree for my report to be made available under an Open Access Creative Commons CC-BY license (http://creativecommons.org/licenses/by/4.0/). I understand that any comments which I do not wish to be included in my named report can be included as confidential comments to the editors, which will not be published.

Choose an item.

To further support our reviewers, we have joined with Publons, where you can gain additional credit to further highlight your hard work (see: https://publons.com/journal/530/gigascience). On publication of this paper, your review will be automatically added to Publons, you can then choose whether or not to claim your Publons credit. I understand this statement.

Yes Choose an item.
