# Supplementary material for: Comparative analysis of common alignment tools for single-cell RNA sequencing
Source: Gigascience. 2022 Jan 27;11:giac001. doi: 10.1093/gigascience/giac001 (PMC8848315; doi:10.1093/gigascience/giac001)
Supplement: giac001_Reviewer_3_Report_Original_Submission — Hirak Sarkar -- 7/7/2021 Reviewed [file giac001_reviewer_3_report_original_submission.pdf]

Reviewer Report

Title: Comparative Analysis of common alignment tools for single cell RNA sequencing

Version: Original Submission **Date:** 7/7/2021

Reviewer name: Hirak Sarkar

Reviewer Comments to Author:

Producing single-cell count matrix from the raw barcoded read sequences consists of several contributing steps such as whitelisting, correcting cell barcodes, resolving multi-mapped reads, etc. Each step can potentially introduce variability in the resulting count matrix depending on the specific algorithm adapted by the tool used. Bruning et al. attempted to disentangle these effects using the most popular scRNA-seq quantification tools such as Cell Ranger 5, STARsolo, Kallisto, and Alevin. The manuscript is well-written and would add considerable value to the broad single-cell research community. I have a few concerns about the current draft of the manuscript that can be addressed in a revision.

- The `scina` tool is used to construct an "artificial ground truth". The consensus of two or more mappers are used to arrive at this reference annotation. In my opinion, the consensus can lead to a biased reference, especially since STARSolo and Cell Ranger5 follow a very similar pipeline; it is expected, by design, that those tools would have highly-overlapping results.

I suggest that the simulated datasets from the pre-decided clusters might be more appropriate for an unbiased evaluation (The recent paper from Kaminow et al. https://www.biorxiv.org/content/10.1101/2021.05.05.442755v1.full has similar simulations). Having said that, the current consensus-based analysis in my opinion should give a reasonable reference for most of the cells, but a more principled simulation is required to identify the extreme cases where each of the tools might show variable assignments.

-The Sankey plots (Supp Figure 5) and the heatmaps (Supp Figure 6) represent the mutual agreement from different tools. As the `scina` clusters are used as ground truth, a more direct qualitative measure such as precision/recall would be more helpful.

To be more specific, the resolution parameter of `FindCluster` could be tuned (now set to 0.12/0.15) to produce the same number of clusters present in the ground truth. Each predicted cluster can then be assigned to a ground truth cluster greedily. The number of `mismapped` cells can be further categorized as `false-positive` or `false-negative`.

- The variability of different tools on the three real datasets is worth exploring in depth. For example, quoting from the paper, "Alevin detected more cells with less genes per cell in the PBMC and Endothelial dataset. However, it detected less cells with more genes per cell in the Cardiac dataset." It would be interesting to understand the origin of these variations and what authors hypothesize, e.g. apart from mapping/alignment there are other additional steps in the quantification pipeline that could potentially lead to variation in the detected cells and respective gene count. The tools can also have underlying algorithmic biases that are worth exploring.

- "We could show that Alevin often detects unique barcodes, which were not identified by the other

tools. These barcodes had very low UMI content and were not listed in the 10X whitelist.", the alevin --whitelist option (https://salmon.readthedocs.io/en/develop/alevin.html#whitelist) enables use of any external filtered whitelist while running alevin. I wonder if using this option would change the behavior mentioned in the manuscript.
- The manuscript raises the important question of multi-mapped reads across cell-types, it would be interesting to quantify the percentage of reads that are discarded as multi-mapped by different tools (those which discard). If that percentage is substantial, then the difference in handling such ambiguous reads through EM-like algorithm might be promising.

Plots and Figures

-Intersection Plots

The minor differences in the y axis of the intersection plots (Fig. 4, supp fig. 3 etc.) are not pronounced. (log-scale might help)

Overview Figure

The manuscript correctly pointed out how different intermediate steps contribute to the general variance in the downstream results. An overview figure with a flow chart of a typical scRNA-seq quantification pipeline will be beneficial.

Minor Concerns

There is a spelling mistake in the abstract `celtype` -> `cell-type`

Possible incomplete sentence : "The recommended annotation from 10X, which only contains genes with the biotypes protein coding and long non-coding, might lead to an overestimation of mitochondrial gene expression respectively the absence of other gene types."

Methods

Are the methods appropriate to the aims of the study, are they well described, and are necessary controls included? Choose an item.

Conclusions

Are the conclusions adequately supported by the data shown? Choose an item.

Reporting Standards

Does the manuscript adhere to the journal's guidelines on minimum standards of reporting? Choose an item.

Choose an item.

Statistics

Are you able to assess all statistics in the manuscript, including the appropriateness of statistical tests used? Choose an item.

Quality of Written English

Please indicate the quality of language in the manuscript: Choose an item.

Declaration of Competing Interests

Please complete a declaration of competing interests, considering the following questions:

- Have you in the past five years received reimbursements, fees, funding, or salary from an organisation that may in any way gain or lose financially from the publication of this manuscript, either now or in the future?
- Do you hold any stocks or shares in an organisation that may in any way gain or lose financially from the publication of this manuscript, either now or in the future?
- Do you hold or are you currently applying for any patents relating to the content of the manuscript?
- Have you received reimbursements, fees, funding, or salary from an organization that holds or has applied for patents relating to the content of the manuscript?
- Do you have any other financial competing interests?
- Do you have any non-financial competing interests in relation to this paper?

If you can answer no to all of the above, write 'I declare that I have no competing interests' below. If your reply is yes to any, please give details below.

I declare that I have no competing interests

I agree to the open peer review policy of the journal. I understand that my name will be included on my report to the authors and, if the manuscript is accepted for publication, my named report including any attachments I upload will be posted on the website along with the authors' responses. I agree for my report to be made available under an Open Access Creative Commons CC-BY license (http://creativecommons.org/licenses/by/4.0/). I understand that any comments which I do not wish to be included in my named report can be included as confidential comments to the editors, which will not be published.

Choose an item.

To further support our reviewers, we have joined with Publons, where you can gain additional credit to further highlight your hard work (see: https://publons.com/journal/530/gigascience). On publication of this paper, your review will be automatically added to Publons, you can then choose whether or not to claim your Publons credit. I understand this statement.

Yes Choose an item.
