# Supplementary material for: Comparative analysis of common alignment tools for single-cell RNA sequencing
Source: Gigascience. 2022 Jan 27;11:giac001. doi: 10.1093/gigascience/giac001 (PMC8848315; doi:10.1093/gigascience/giac001)
Supplement: giac001_Reviewer_3_Report_Revision_1 — Hirak Sarkar -- 10/27/2021 Reviewed [file giac001_reviewer_3_report_revision_1.pdf]

Reviewer Report

Title: Comparative Analysis of common alignment tools for single cell RNA sequencing

Version: Revision 1 **Date:** 10/27/2021

Reviewer name: Hirak Sarkar

Reviewer Comments to Author:

The authors had resolved most of the issues in their revision. There are still some crucial issues with the current manuscript which in my opinion need to be addressed
Major concerns
1. alevin-fry is added to the benchmarks, but I am not sure what is the exact mode the alevin fry is run in. From https://github.com/rahmsen/BenchmarkAlignment/blob/main/mapping/MapAlevin-fry.sh#L63 it seems that alevin-fry is run in sketch mode but the actual execution of the the https://github.com/rahmsen/BenchmarkAlignment/blob/main/mapping/MapAlevin-fry.sh#L85 does not seem to run sketch-mode. I did not find any mention of the `sketch-mode` in the manuscript. According to this preprint (https://www.biorxiv.org/content/10.1101/2021.06.29.450377v2) the runtime plot in Figure 1 in the current manuscript are quite different from the preprint.
2. I am afraid the github repo in its current format is not reproducible.
- I tried running the commands from https://github.com/rahmsen/BenchmarkAlignment/blob/main/mapping/commands_mapping.txt, I could not find `Homo_sapiens.GRCh38.97.cellranger_filtered.gtf` as non of the commands create/download this file. Only one version of cell ranger was downloaded.
- If download scripts for the data can also be added to reproduction script that would be great.
3. When attempting to examine and understand some of the mapping commands used in https://github.com/rahmsen/BenchmarkAlignment/blob/main/mapping/commands_mapping.txt, the values being passed to some of the commands seem a bit confusing. For example, when `STARsolo` is being run on the filtered index, it is being given the path to a human index `-i ${main_outpath}references/starsolo/human/index_filtered` (line 286). But when the unfiltered index is being provided (on line 305) it is seemingly being given the path to a mouse index `-i ${main_outpath}references/starsolo/mouse/index_unfiltered`. The similar naming convention can found in the same script https://github.com/rahmsen/BenchmarkAlignment/blob/main/mapping/commands_mapping.txt#L325-L330 and https://github.com/rahmsen/BenchmarkAlignment/blob/main/mapping/commands_mapping.txt#L345-L350, but this isn't the case for all methods (some e.g. are always passed a "human" index for the PBMC dataset). I would request authors refine the naming convention and explain if this was, indeed, the intended way of running PBMC.
4. It would be great to have some more details about exactly how the tool was run. For example, looking through the repository, it seemed that a spliced and intronic reference was prepared, but the resulting count file looks to be read in using the standard mtx loading procedure and it is not clear if the unspliced

/ spliced / ambiguous status of UMIs is accounted for.
Currently the scripts are dependent on assumed directory structure with downloaded datasets. While in some cases obtaining the data might not be straight forward, I would suggest authors to provide an end-to-end reproduction script for at least one well-known dataset such as PBMC.
The recommended way of running `alevin-fry` can be obtained from https://github.com/COMBINE-lab/alevin-fry#a-quick-start-run-through-on-sample-data. It would be interesting to see the results from such a run.
Minor concerns
1. Please mention the salmon version in the manuscript.
2. "Alevin-fry seems to have improved its barcode correction as here the decrease is not present." this sentence is not clear to me, it could be made more comprehensible.

Methods

Are the methods appropriate to the aims of the study, are they well described, and are necessary controls included? Choose an item.

Conclusions

Are the conclusions adequately supported by the data shown? Choose an item.

Reporting Standards

Does the manuscript adhere to the journal's guidelines on minimum standards of reporting? Choose an item.

Choose an item.

Statistics

Are you able to assess all statistics in the manuscript, including the appropriateness of statistical tests used? Choose an item.

Quality of Written English

Please indicate the quality of language in the manuscript: Choose an item.

Declaration of Competing Interests

Please complete a declaration of competing interests, considering the following questions:

- Have you in the past five years received reimbursements, fees, funding, or salary from an organisation that may in any way gain or lose financially from the publication of this manuscript, either now or in the future?

- Do you hold any stocks or shares in an organisation that may in any way gain or lose financially from the publication of this manuscript, either now or in the future?
- Do you hold or are you currently applying for any patents relating to the content of the manuscript?
- Have you received reimbursements, fees, funding, or salary from an organization that holds or has applied for patents relating to the content of the manuscript?
- Do you have any other financial competing interests?
- Do you have any non-financial competing interests in relation to this paper?

If you can answer no to all of the above, write 'I declare that I have no competing interests' below. If your reply is yes to any, please give details below.

I declare that I have no competing interests

I agree to the open peer review policy of the journal. I understand that my name will be included on my report to the authors and, if the manuscript is accepted for publication, my named report including any attachments I upload will be posted on the website along with the authors' responses. I agree for my report to be made available under an Open Access Creative Commons CC-BY license (http://creativecommons.org/licenses/by/4.0/). I understand that any comments which I do not wish to be included in my named report can be included as confidential comments to the editors, which will not be published.

Choose an item.

To further support our reviewers, we have joined with Publons, where you can gain additional credit to further highlight your hard work (see: https://publons.com/journal/530/gigascience). On publication of this paper, your review will be automatically added to Publons, you can then choose whether or not to claim your Publons credit. I understand this statement.

Yes Choose an item.
